# Supplementary material for: Physiological and Transcriptomic Analyses Reveal the Mechanisms Underlying Methyl Jasmonate-Induced Mannitol Stress Resistance in Banana
Source: Plants (Basel). 2024 Mar 3;13(5):712. doi: 10.3390/plants13050712 (PMC10935223; doi:10.3390/plants13050712)
Supplement: Supplementary file 1 [file plants-13-00712-s001.zip › plants-2724498-supplementary.pdf]

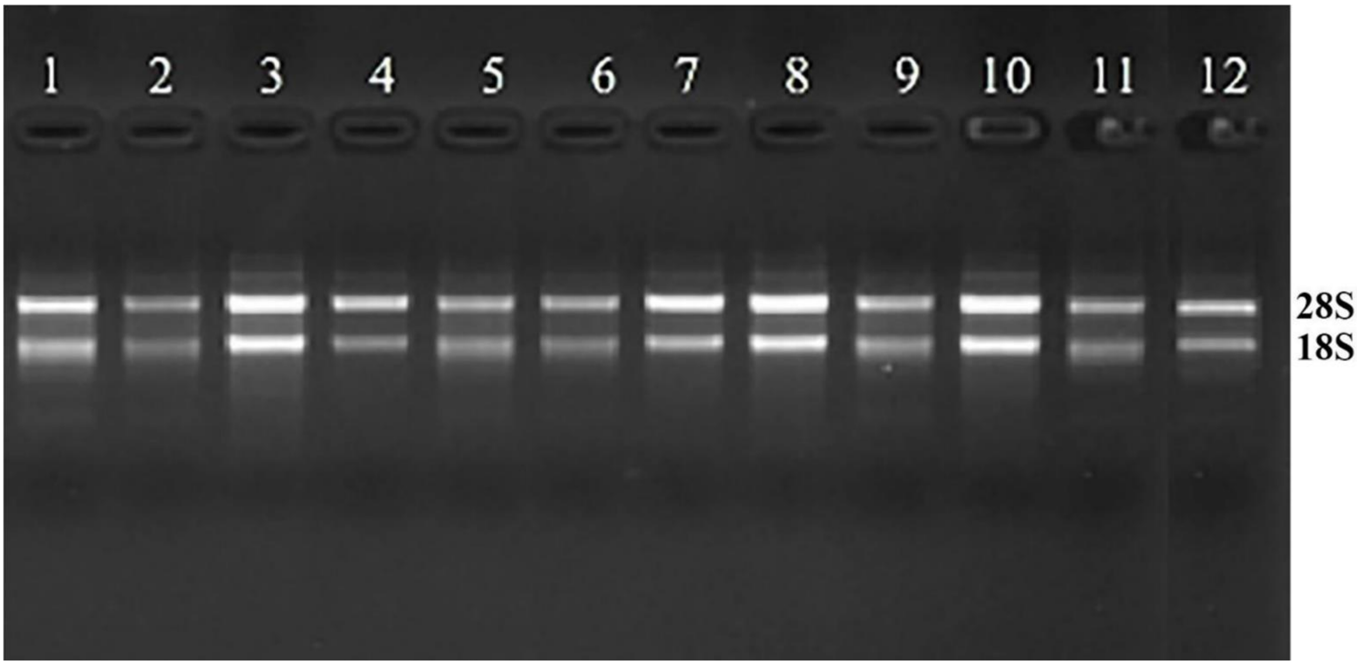

Figure S1. Agarose gel electrophoresis of total RNA. The RNA samples contained two bands: 28S RNA (upper band) and 18S RNA (lower band).

Table S1. Results of RNA analysis using Nanodrop2100, Qubit2.0, and Agilent Bioanalyzer 2100

Sample name	Concentration	OD260/280	OD260/230	25S/18S	RIN
A0_1	180	2.25	1.324	1.5	7.8
A0_2	120	2.308	0.632	1.4	7.7
A0_3	112	2.24	2.154	1.2	7.6
B0_1	164	2.05	1.39	1.3	8.2
B0_2	326	2.296	2.296	2.1	8.6
B0_3	148	2.643	1.644	1.4	9
A8_1	130	2.407	1.512	1.1	7
A8_2	142	2.29	1.479	1.5	8
A8_3	160	2.222	1.702	1.2	7.5
B8_1	192	2.182	2.087	1.7	8.4
B8_2	194	2.205	2.021	1.8	8.6
B8_3	248	2.214	1.771	1.5	9.1

Table S2. Statistics of sequencing data

Sample name	Raw reads	Clean reads	Clean bases	Error rate (%)	Q20 (%)	Q30 (%)	GC content (%)
AO_1	57009144	55742402	8.36G	0.03	95.19	88.41	54.27
AO_2	52224516	51189386	7.68G	0.03	97.33	92.91	53.78
AO_3	52699190	51453798	7.72G	0.03	96.8	91.81	55.71
BO_1	49539480	48587416	7.29G	0.03	97.1	92.39	52.89
BO_2	48632594	47326184	7.1G	0.03	97.34	92.88	52.02
BO_3	54240738	53180196	7.98G	0.03	97.35	92.85	51.96
A8_1	51267750	50186822	7.53G	0.03	96.8	91.8	54.92
A8_2	40393158	39442658	5.92G	0.03	97.33	92.84	53.69
A8_3	53700548	52810980	7.92G	0.03	95.05	88.1	53.13
B8_1	52305864	50887470	7.63G	0.03	97.67	93.54	52.56
B8_2	50097078	48965870	7.34G	0.03	97.57	93.39	53.38
B8_3	55754868	54681188	8.2G	0.03	95.28	88.54	53.45

Table S3. Alignment of gene sequences with banana A genome

Sample name	Total reads	Total mapped	Multiple mapped	Uniquely mapped	Exon	Intron	Intergenic
A0_1	55742402	49356837 (88.54%)	683927 (1.23%)	48672910 (87.32%)	94.70%	2.70%	2.50%
A0_2	51189386	46660088 (91.15%)	645955 (1.26%)	46014133 (89.89%)	94.70%	2.90%	2.40%
A0_3	51453798	46846266 (91.05%)	608535 (1.18%)	46237731 (89.86%)	96.10%	1.90%	1.90%
B0_1	48587416	43814296 (90.18%)	679097 (1.4%)	43135199 (88.78%)	95.90%	2.20%	1.95%
B0_2	47326184	42929483 (90.71%)	656228 (1.39%)	42273255 (89.32%)	95.30%	2.60%	2.10%
B0_3	53180196	48275817 (90.78%)	719892 (1.35%)	47555925 (89.42%)	95.60%	2.50%	1.90%
A8_1	50186822	45120327 (89.9%)	1928615 (3.84%)	43191712 (86.06%)	96.80%	1.60%	1.60%
A8_2	39442658	35809020 (90.79%)	1515021 (3.84%)	34293999 (86.95%)	95.70%	2.50%	1.80%
A8_3	52810980	46253336 (87.58%)	1842882 (3.49%)	44410454 (84.09%)	95.20%	2.70%	2.10%
B8_1	50887470	46343103 (91.07%)	1589207 (3.12%)	44753896 (87.95%)	95.20%	2.20%	2.60%
B8_2	48965870	44319372 (90.51%)	1362100 (2.78%)	42957272 (87.73%)	95.50%	1.80%	2.70%
B8_3	54681188	47868042 (87.54%)	1458909 (2.67%)	46409133 (84.87%)	96.90%	1.30%	1.80%

Table S4. Number of differentially expressed genes

FPKM Interval	0~1	1~3	3~15	15~60	>60
AO_1	12776(35.14%)	5033(13.84%)	11033(30.35%)	5671(15.60%)	1842(5.07%)
AO_2	12538(34.49%)	4899(13.48%)	10798(29.70%)	6078(16.72%)	2042(5.62%)
AO_3	13070(35.95%)	5071(13.95%)	11167(30.72%)	5311(14.61%)	1736(4.78%)
BO_1	10229(28.14%)	5366(14.76%)	11778(32.40%)	6763(18.60%)	2219(6.10%)
BO_2	9993(27.49%)	5160(14.19%)	11715(32.22%)	7200(19.80%)	2287(6.29%)
BO_3	9816(27.00%)	5234(14.40%)	11744(32.30%)	7284(20.04%)	2277(6.26%)
A8_1	12928(35.56%)	5056(13.91%)	10589(29.13%)	5684(15.63%)	2098(5.77%)
A8_2	12543(34.50%)	4785(13.16%)	10742(29.55%)	6240(17.16%)	2045(5.63%)

A8_3	12717(34.98%)	4855(13.35%)	10494(28.87%)	6220(17.11%)	2069(5.69%)
B8_1	12623(34.72%)	5255(14.45%)	10290(28.30%)	5895(16.22%)	2292(6.30%)
B8_2	13524(37.20%)	5248(14.44%)	9837(27.06%)	5486(15.09%)	2260(6.22%)
B8_3	13264(36.48%)	5411(14.88%)	9904(27.24%)	5395(14.84%)	2381(6.55%)

Table S5. Promoter sequences of *LOX* genes

Gene name	Sequence
Ma06_g26840	TGCCATGACCGGTCTCTAACAATATCTAGTGGTAACGACAATTTATAATAGCAAAATTTATGA<br>AAGAGATGAAAAACAATATTAAGAATGGAACCTATATATATTGACAGTATCAGAAAATGAA<br>ATGTCCTATATTCATATCATGCCATTCAAGTTTCATGCATTGGATTTTGAAGTAGTGTACTTAT<br>CCACATCTAATGACTGCTAAGAAAACAAATCATATCAGGTAGATCATTTAATATTGTAAATA<br>TAATGATAAAAAGAACACAACGATCATTGTACCCAATTGGGACCAGGATGAATTATGACCG<br>ACCTTCTGAACTATTCCCAATTGGGACCAGGATGAATTGTGCACTTGGAAAGCATGAGTTGTA<br>TACACATCACTGTTGTGAAGCCTATCCTTGGCACATCATTTTTGTGTGATCGATCTACCAACTG<br>AACCCTCATTTAACACCTTTTTTAATAACATGAGAATAAGACAGTGATATTTGTCATTTTTTAT<br>AACAAATATGACCAATAATTGATCATAATAATAATAAAGACAATAAAGATAGCCTATGGGA<br>CCATAATAATAATATTATTATTATGGTCTATAATAATTTGTGTGCCATCTGAATCAATCAAGA<br>CAATAAAGATAGCCTAGGGGATAATAATAATAATAATAATTATTATTAGTGTAAACAACTTT<br>ATGTCGTGGCCTCGGGGTCGACGCGACTTGGTTGAGGTCCGAATGACGGGGATCTGGTACGG<br>CGTTCCTCGAGATTGCCCAGGCGGCCGATCACGGTGGTCCGGCCGGGACGTTACATCGAGAG<br>GAAAGCTTTTCCACAGCGCCGGGAAGAAGCAACCCCGTCCTTACACCTGCACACAAGTCGA<br>GTCGGAAGCTCGGCCCGACCCCTCCGACGATCAAGTTAGTGGATGTGGAGGGGGTTTCGAAG<br>TGCTCTCCCCCCTCGTTTAGAACTCGGGGGTATTTATAGGGCAGTTTAATGTTACTTGATGTGC<br>CTACCTGCGGGAGCAGGATCGTACCTCTGATGGCGTCTGACATCGCCATTGGCATTGCGTGG<br>AGAACTGAACTGCCGTAGGGCATGGGCGAGCCTCGGTCGTCGTTCTCCTCTGCCTCGGCCAA<br>GCATGTTGGGTCAGACAACGATGAAGTCGTTGTTTGAGAGCGGGTGACGTCAGCGCACATCG<br>CGTCAGCATTATTGCCCTCTTTGGGGCAGAGTGTCGTCCAAGCGTGGCTAATGTCGTGGCGCG<br>TCATGTCGTCATTATTACCCTCATTAATAATAATAATAATAATAATAATATAATCCATATGAT<br>AATTATACTAAGGAGGGAGATGGATAAAGATTAGAATTTATATTATATAATAATATCTTTATT<br>ATTGGTATTATTACTTACGTTCGAGTTTGGAATCGACAGTTCAACCTCCACACAAGATGGAAT<br>CAGAACCATCATTTTTTTTTTAATTTTATTTAATTTAATTTTTATTATAGTGTTTAATGTTTATTT<br>CTTTTTATTTTCATCTGGTTCTGATTTTGATTTCAACGTGAAATGGAACCCCCCATCCGATTCCG<br>ATTCGATTCAGATCTATGAAATCATTGATCGTTTTCGTTGTAATTTTCACCGGTTCGTTCAAAT<br>ATCACTATTTGTTTGTATCATTGTTATATTTATTTTGTGTGGAAAAGATATACAACTAAGTCTC<br>GCGCAGATGACACATAAATCAGAGTTCACATTGGACCGCGATTGGCGGCCATGTGAAACGC<br>ATGGATGCTCAATTATCAGGGAGTGGGTCCCAACAAAACACCTATTTAATTCAGTGGACCCA<br>ACCACGTAAAATTATTTGGGGAAGCATTTGTGGACACGAGAAACCCGTGCTTGTTCGACACA<br>GGCGGATGCTCTGCATCAATAAATACCACCTCCGCTGCAATCGATCTCACTGCAACGTTCACT<br>CTCTAAGCTATTAGTCGTCGCTATCCACTGTGTTACAGGAAAAAGGGAACCGAAGAT
Ma06_g26890	TTACCCTTCATTGGAGGGGTTTTCCACGTATATCTTGGTATTTATTTGATTGTGATTTCATTTAA<br>TTCCACTGTATATCATGGCCTGCTAGTATTTGTTCATATACAAAGGTTATTCCGCTTTTTATCCC

CATCAACTAGTATCAGAGTAAGGGTTTTGATGATTTAATTTTGTATTTGAACATGGAGGCCAG
TAATGTTTCTCACATGATTAGTTTAAATGGAAACAATTGGATGATATAGAAACCAAGAATGG
AAGATCTCTTGTATTGTAAAGATTTGTATGGACCTTTGCAGGGGGATAGTGCAAAACTCATAA
CTATGACAGATGATGAGTGGAAGAGGTTAGATTGAAAAATAATTAGATTTATTCGACAGTGG
CTTGATGATAGTGTCTTTCACTATGTTTCTACTGAAATTTCTGCATATTCTCTTTGGAAAAAATT
GAAAAGTCTCTATGAAAGAAAAATAGCTGGCAACAAAGCTTTTTTGATCAGAAAACTTGTGA
ACGTAAAATATAGAGAGGGTGCTTCTATTGCTGAACATTTGAATGAAATACAAAGTATTACT
AACCAGTTATCCTCTATAAAAATGTCTCTTGATGATGAGTTGCAGGCATTGTTACTTCTTAGTT
CATTACTAGAAAGTTGAGAGACACTAGTAGTTTCCCTCAGTAATTCTACGCCAGATGGTGTTG
TCACTATGAGTCAAGTAACAAGCAGTTTGTTGAATGAGGAGTTGAGAAGAAAGATTTCGGCA
ACATCTCAGAATAATTCACAGGCACTTATCTTAGAGAACAAAGGAAGGTCAAAGTCTAGAA
GCAGTTCACGCATAAGTAGAAGCAAGTCAAGATCAAGAAAAGATATTGTTTGCTATAATTGT
AGTGAGAAAGGATATTACAAGAACCAATGTAAGCAACCTAAGAAGAGCAAGAAAAAGGGA
AAAAAAGTGGAGTCTATAGAGTCAAAAGATAATATCAACTAGACCTAAGAGGTATACACCA
CGGGGCAAAATCAATTAAATGAAATAACAATCAAATAAATATCAAGATATATGTGGAAAAC
CCCTCCAATGAAGGGTAAAAACCACGGGGCAAACTAGAGATAATCCACTATGAGAATAATG
AATATACAAATCTCAATCTCTTACCCTAAACCCTAGCAACAATCACAAGAGAATAACTAGGA
TACAAGGATCACATCACTGCCTACAATAATTGAGAACGTAGCCACCACACCCCTCTTAATGT
TAATTAGGGTTAGGTTAAGAGGGGATGAGCTATGGGCTGCCCAAGCCTACTATGGGCTGAAT
TTGTGGACTGCCAATCTAAGAATATGAGCCTAAGAATTTAATAAATTTAAATATGCAAATAA
TTTTGTACCATTAAAGCATGTAGATTATTTTTATGTCTTAAAAGAGCATGGTTTCAATGCAACA
TATGTGCATTAAGACAATCCAGACAAATGTAATGCACAATGTATTAAGACAAAAAAATCATC
TCGATGATAAGTAATTTTTCTACTAATTTAGAAATGTAGTTAAAAGTCTAAGTTAACATAACA
CTTACTAAAATCGCTGCAATTAGTCATTTATAAAACTAAGTCTAAGTATTTGTTTGTATCATTG
TTATATTTATTTTGTGTGGAAAAGATATACAACTAAGTCTCGCGCAGATGACACATAAATCAG
AGTTCACATTGGACCGCGATTGGCGGCCATGTGAAACGCATGGATGCTCAATTATTTGCGTTA
TCCGCACATTGAAACAGTGATCAGGGAGTGGGTCCCGACAAAACACCTATTTAATTCAGTGG
ACCCAACCACGTAAAATTATTTGGGGAAGCATTTATGGACACGAGAAACCCGTGCTTGTTCG
ACACGCAGGCGGATGCTCTGCATCAATAAATACCACCTCCGCTGCAATCGATCTCACTGCAA
CGTTCACTCTCTTAGCTATTAGTCGTCGCTATCCACTGTGTTACAGGAAAAAGAGAACCGAAG
A

Ma01_g180
60

TAGGGTCGCCGACGGTGGCGCTGATGAGCTGGAAGGAGATGCATCGGCCGAGGAGCTCGAG
CACGTTGTCGATGACGGTGCCGCCGAAATCGTTGAAGCCGAGGACGTTCTTCCGGATCAACA
CCACCGTTCCTCTCACCTTCACCCCATGCGACCCGCCGCCGACGATGTGACTCAGCATGTCTC
CAAGTAGCGTCATCGTACAACAGGAGCCAGAGAGATGAGGAATGGTGTGCTGAGGTGTGTG
GTGGAAGGGGCTACTTATAGATGTTCTTGGATCGAAGAGTGCAGATTACTTATGTACGTAATG
CAGTCTCTCCCTTATCTTAAACCGAGCATGCCAAGAGAACCCATATAAATCTAATTCTAAAAC
ACTATATGAGATTATACCCATGTGACATTTATTTGTCATATATCATCGACCGGAGAATACAGC
ATAAAGTTACATGTCTCGTAAGTCAAAACGCACCGAAGTTTCAGAACACGTGTCCGTTCACG
AGGCTAGATTCGATCGTCCGAATATTCAATCACTATCACAATTGGATCGTATTAAGGCGGTAA
AAAGATGAAAAGGGTGAATCCAACACATGAGATCGAATCACATGTAAGGAGGTATCGGCCA
TAAAGAAATAATCTTGGATCATTGTCGAGCTGTTTGTCCCATTTACAAACGCATCTATGACCA

AGAATATGAAGTCAAAAGTAACTCTTTGGATGCACGCCTCGCCAAACCCACTAAGGCAATTG
AGGAGGTTAAATTTTATGCAAACAAATAAAAAAATACATGGCTGGATTCACGTTTCAATCAC
GTGCTTTGTTTATTTTCTGATAGGCTGGCAAGGGGACCATGAAGAGACAGTGGCCATTGAGA
AGATAAGAGTCCACATCATAAGCTGCAATAAAAAGGTACATGTGGGAATAATGTAGCAAAG
TGGCCAATTCACGGTGGTGGTCCAAGTAATGACCAACCCTTGAAATATCATTCATGTAGGGTC
AAGATAGAAAAGGATTTAATAATTATATTTAACATAAAATGGGAAAAAATACTTTAAATTTG
TTTTAATATCAATGAATAATATTTTTTGTGATATAGAGAATTTGTCGGGTCACGTCTTATTCTA
TTATTTTTGGTTATATATGATAAGTTAAATTTTTTTCTTTCTTTCTCATCCTTATTTATATTTCTGA
TCCTCAATTTGTGGTCATGTCTACCTCATTGTCTATCGCTACTCTCAATAACGAGATAAATAAA
TGATAAATCGATGTAAAAATATTATTATTTCTTTCATAAATGAGTATATATGATAATACATAA
TGGAAGGATTTAATAATTATATTTAATATAAAATAAAAATGTATTTTAAATTCACTTTAATAT
CAATGAGTAATTATTTTTTGTGATGTAGAGACCCTAATAACACATAATAAAAAGCAGTAATA
TCCTTTTTTTTTCTCTATGACTCTACGTAAATAATCTATGAAAATATATTTACTTTATAAAATCC
TAGTGATGCGTCTCGTTCCTCCATCTTTAAGATACTTGTACGTAAAAATCGATTACGTGAAGA
AATAAAAAAAAATCCTTTAGATTTAAAAAAAAAAATGAAAATTCCTCGTTCGAGTATGAAAGA
AATCTTTTATGATTTAATATTTATTTAATTTGTATATATTGGGTCAAAGAGGAAGATTGAGATA
AAAGATGAAAAAAATTCTTTGATATTGATGTGAGAGGTGTATAAAGATATTTAGGTTAAAAA
TAAGTGAACCTTTAATTTGATCCCATTGTTGAGTTACGGTGTTTCACGTTTCAAGTCTTCTCTG
GCCAACTTATTATAAGACCTCCAAGAATCCATAAACTATGTTGCATGTGACCTGATATTATTA
TAGGAGCAGGCTTCTCGGACTCATAAAATTAATTATTATTATGAAAATATAAAATTTAATTTA
ATTTAATGTTCCGATACTATTGTCTTGATTCCCACGCCGTGGTATTGAGTCCAAGG
CACGAGTTTCTAAATATTGTTGGGTATATCTAATATGATCGTTCTAATGTTCAAGTCTGTAAAT
AGTCAAAGATGAGAATTAGGAAAAAAGATTGGTATTTGTAATCATTTATGAAACAAAGAATA
ATCTTTCCTCTCACATTATGTATTGGATTGATTAGGTTCTATATCTTGACTTATGATTGATTTGA
AATATTGATAGGTATTGCTTGTTATATGTTATGAATGAGGGTTAAGTTAGAGAGAGATGTGTT
TGTCCAAAATTTAATGAAAGTTGTTAATGCAAGAGATGAAGAGCAGAGAGATGGATAAGAA
CATCATCCCCTCTCAAGCCAGGATGGAGAAAGATGGAAGAAGATATCTCAACACCTCATCAT
CGAGCAATACATATAAAAGAATCATATCAGAGACAAGTGGTGCCAAAAGGGTCACTGTCTA
TACGTCACATTGTGTACTGCAACACAAGATTCTCATGATAAGGCATTTTATATCTAATTTAATT
GGATAGTAATTTTAATTATATTAAAACTCTTAAAAATAATCTTTTTATTTGAATATAGAGTTTA
ATTTAATATTACAATAAGAGATACTAAATCTTTAGTCTAGAAATACTTTTATCATGACCCGGT
Ma01_g16400
ATATTCGTTGAGTTTGAATCGAGACCTTCGTACTAAATCGAGAAGATAATTAATCTATTAATT
TATTGATCAAAATATTTAAGTTGGGATTGATAATAATAATATTCTTATCAAATTTTTATAGATC
GATCTTAACTTTATAATAATCGAAATGTTACAATCTCATCTACCCCATTGACGGGAGATAATA
TTTTAGGAAACTACTGATTGAGATTTGAAAAAGATCTTCCCAAATATTAATTTTGTTCCTCTCA
TACTGGTCAATTATTGTTTAGTGTCATCCTCACCTAATATTGCCCTCATCAACTTTTCTTAAAG
AATATGTGAGCCATTGCAATAATTGCTAAGGGACCAACTTGTAGTACATTATTCCTTGTCTCC
TTATTTAATCTCCTCCTGCCTATCCCATATGACAATCTCCATTTATTTTTATGAATTTTCTATAA
AAAATAAACTACATTTAATGTTTTCTTAGTTTAGAAATAGAGTTTATGGTGTTGAGTGTTTGGA
GGAAAACTATATTACATGGAAAAAATATATATATAATTATGTATGACGAAAGATGTCTTTAA
TCTCTCTTTCAATAAAATCCCATCACTCTTCCTCTCTCTTTCAAAACTTATGGGGTCATCATCCT
ACATGAGCTGTCAGATTAAAAAAAGAGGCACAGGCAGTCTAGTGGTGCAACTTTTTCCACAT

CTTCTTTTATATTCCAAGCAAACTTGCACACTTGAGCTTTCATTTTTTTTTGTTTTGCAACCTTTC
TTATCAAGACATTGGAAGATCATTACATCCTTTTCCACTCAAATCCTATGAGATATTGTGTGTT
TGGTACTGAGAAAGAGATGATTACTTCCCTGTTTTTTGGTCCAAATGATAAAGCAAATTCATC
AGAATCATGAAATATAATTCAGAAATGTAAACCAAAATCTGAAAGCAAATCATGTCCATCA
GCTGAATCAAGAAAGACCAAGAAGCAAACAGTACTCGCAACAGCTCTCTCCCCATTCAATTC
AGGGACCATCTACTGGGCTTGGGAACGAGAACAGTAGAGGAGCGATGCAGGAAGCTACGA
AGCTCTGAAAGCTCATAGCAGGTTGAAGAAGACCCAGCAGGAGACGACGTCACGTGGTTCA
CATGGTAGAAGTGGGTCCACCATGGGGTCCATATGATGTGGCCATCTGATGCGTCGCCTCCA
ACATGGCATGTGACTTGGGACAGAAATATAGATCGTTCTACGCTTTGGCTCCCCATTTGCCTC
TATAAGTACCTCCCTATATCCTCCAACACATCCTCATCTCAGGCAGTGCATCTGCTACTTGTTA
CTTCCTCTCTTCCTCTCTCGCTTGGGTTGGTGTATCGGGAGGCAAGA
TGGTAACGACAATTTATAATAGCAAAATTTATGAAAGAGATGAAAAACAATATTAAGAATG
GAACCTATATATATTGACAGTATCAGAAAATGAAATGTCCTATATTCATATCATGCCATTCAA
GTTTCATGCATTGGATTTTGAAGTAGTGTACTTATCCACATCTAATGATTGCTAAGAAAACAA
ATCATATCAGGTAGATCATTTAATATTGTAAATATAATGATAAAAAGAACACAACGATCATT
GTACCCAATTGGGACCAGGATGAATTATGACCGAGAGTTCATACCTTCTGAACTATTCCCAAT
TGGGACCAGGATGAATTGTGCACTTGGAAAGCATGAGTTGTATACACATCACTGTTGTGAAG
CCTATCCTTGGCACTTCATTTTTGTGTGATCGATCTACCAACTGAACCCTCATTTAACACCTTTT
TTAATAACATGAGAATAAGACAGTGATATTTGTCATTTTTTATAACAAATATGACCAATAATT
GATCATTATAATAATAAAGACAATAAAGATAGCCTATGGGACCATAATAATAATAATATTAT
TATGGTCCATAATAATTTGTGTGCCATCTGAATCAATCAAGACAATAAAGATAGCCTAGGGG
ATAATTATTATTATTATTATTATTATTAGTGTAAACAACTTTATGTCGTGGCCTCGGGGCCGAC
GCGACTTGGTTGGGGTCCGAATGACGGGGATCTGGTACGACGTTCCTCGAGATTGCCCAGGC
GACCGATCACGGTGGTCCGGCCGGGACGTTACATCGAGAGGAAAGCTTTTCCACAGCGCCGA
GAAGAAGCAACCCCGTCCTTACACCTGCACACAAGTCGAGTCGGAAGCTCGGCCCAACCCC
Ma06_g268 TCCGACGATCAAGTTAGTGGATGTGGAGGGGGTTTCGAAGTGCTCTCCCCCCTCGTTTAGAAC
50 TCGGGGGTATTTATAGGGCAGTTTAGTGTTACTTGATGTGCTTGCCTGCGGGAGCAGGATCGT
ACCTCTGATGGCGTCTGACATCGTCATTGGCATTGCGTGGAGAACTGAACTGCCGTAGGGCA
TGGGCGAGCCTCGGTCGTCGTTCTCCTCTGCCTCGGCCAAGCATGTTGGGTCAGACAACGATG
AAGTCGTTGTCTGAGAGCGGGTGACGTCAGTGCACATCGTGTCAGTATTATTGTCCTCTTTGG
GGTAGAGTGTCATCCAAGCATGACTGACGTCGTGGCGCGTCATGTCGTCATTATTACCCTCAT
CAATAATAATAATAATAATAATAATATAGTCCATATGATAATTATACTAAAGAGGGAGATGG
ATAAAGATTAGAATTTATATTATATAATAATATCTTTATTATTGGTATTATTACTTACGTTCGA
GTTTGGAATCGACAGTTCAACCTCCACACAAGATGGAATCAGAACCATAAATTTTTTTTAATT
TAATTTTTATTATAGTGTTTAATGTTTATTTCTTTTTATTTTCATCTGGTTCTGATTTTGATTTCAA
CGTGAAATCGAACCCCCCATCCGATTCAGATTCGATTAAGATCTATGAAATCATTGATCGTTG
TCGTTCTAATTTTCACCGGTTCGTTCAAATATCACTATTTGTTTGTATCATTGTTATATTTATTTT
GTGTGGAAAAGATATACAACTAAGTCTCGCGCAGATGACACATAAATCAGAGTTCACATTGG
ACCGCGATTGGCGGCCATGTGAAACGCATGGATGCTCAATTATTTGTGTTATCCGCACATTGA
AACAGTGATCAGGGAGTGGGTCCCGAGAAAACACCTATTTAATTCAGTGGACCCAACCACGT
AAAATTATTTGGGGAAGCATTTGTGGACACGAGAAACCCGTGCTTGTTCGACACGCAGGCGG

Ma03_g11520	ATGCTCTGCGTCAATAAATACCACCTCCGCTGCAATCGATCTCACTGCAACGTTCACTCTCTT<br>AGCTATTAGTCGTCGCTATCCACTGTGTTACAGGAAAAAGAGAACCGAAGAT<br>GCGGAGGAGGAACGCTCGAGAGTGCTGTGATGGACGAAGAGGGCTACGGGTCGGCTAGTCC<br>TTATAGAGGGCTCGCCAGCCGCAGCGGTGACTACAGTCTGCAGCAATCATAGATGAATATTG<br>TATTGTTCATCCATGGCTGCATGATTCACGTGGGAACTGACTGCTCTCTCTATCGTTCATTCTG<br>CTACAAACGATGACTCCTTAGGTAGCAGATTTGCGTCCATTTTTATTCCTCTGTCGATAGCATC<br>TGCGATATTTTACCGATTACGCCGCCGACGACGTGGACAGGGAGATGCAGCGTGTCATGAGG<br>CCCTTCTTCCCCAACTAGCGACTGGTTCCATCAAGATGAAGAGGCACAAGAAAACGAAGAG<br>ACCGAACCGTAGTGCAAGCTGGTCTGCATAGTTCTAACCCCAGCTAAGTTTCAATTGGAATC<br>GAATCTAAATTATTTGGTTATGGAATCTAAATCGACCCTACATGGTTTGGTTTCGATTCTCAAA<br>ATTTAAGAACCATGATCATTGGATCCGCAATCGGGGTTCGGTCTACGTCCTTATGCTTTATGCT<br>AAGCTGTCGATCAACAAGGATGGGGAAGAGGAAAGAAAATATCAATAAGGATACCTCTCCT<br>CTCCTCTACACCACACACGCTTAATCCATGCGAGCGTCCCCCTAAAGAACACATGGAGAAGA<br>AGCTTTCGGGTCCAACATCACCGAATCATGGAGACAATAACTATTAGTCTGTCGATTGCAATC<br>AATTCATCTAAACGAATGATCATTAAGTAGAGACCGATTGACGTGTATGCAATTATTGATTGC<br>AATCAATCTATGTCAAATTAAGTTATAAGAGTGACGAACGAGGTGAAGGAGTACAGTAGATT<br>TGGTGAGCTAAACAAGATTAAGAATCTCAAGAATAATAATAATAAATAATGATGACGAGTTT<br>CTCATGCCACAATCCTTAAGATTTGTGTCACATCAAACTCTAAAATAAGATTGAAGTACAAG<br>CATAATAGAGAGAGAATAAAAGAGAGAGAGATAATTGAGTCACGCATAAATAGAAAAAGA<br>TATTAGAAAATATTTATGTGTGAAAGACATCAAGATAATAATGTCGTATGGATAAAAGTGGA<br>AAGGGCCAAATTATCGATAGGGATTTATTCATAATTTAAAAGATTGATCTTTTGAGTTGGATC<br>GGATCACATTATCGATTTGTAATGGCGGAGTTCGATCTAACCATCACCTTAAAAAAAAAAAA<br>GGAACTTCACGTTGACCATAAATACCTAAAAAATAGGAGGAGGAAATGGTGATCACGGAAG<br>CCCTCGACGTTCTCCTCCACTTATATGCTACGAGCATGAGGAAAAAATGGCGTTTCCCTCCTC<br>CACATATCACCTTTACCCAGACAACACCGGCGTCGCAACCAAGTGGACCACCAAGGAATCA<br>GCTTGTAGTTCCTCGTCCTTCGTCCCCTCCTTTACTTCCATTAAAATGTCACCTCTTTCAATCGA<br>TGTTCGAGAACGATTCCAAGCGCATCTCAATCTCCATCCATCGAAACCGATGCAGATGGCTT<br>GGCGAGTAGCTTCGTTAGTCTACCGATCTCCATCAAAAGTTGTTTTACGTGTGGCCTAAAAGA<br>TGCCATCTGATGTGTCATTTCAAGGAATGAAAGAGATAAAGTTATGAAAAAACTCGATTGAC<br>GTCGGACAAACAATGACGGATTTTGGTTTTTAATGACAGGAGTAGAAATATAAGAAGAGATA<br>AATTAATAGAAAAATATAACTCTACGGAATGAATGAATGCTCTCATGAATGTCTCGCACCTTT<br>CACATGTCTTGTGACAGTGAGAAAAATCCAATCCGTTGATTATCTCTCGATTGATGCATCATA<br>ATATTTTATCATCTTCTATAGACATAGATAAAATTATGAAAAAAATATATGATTTTATCAAAT<br>AAAATGCATGATGTCATTTCTTGGAACAAAGCAATAATTCTAATTTGGTTTGAAAAATAATCT<br>TCCT
Ma08_g14590	AATAATATTTTCTATGAAAATCTGGGCAATACTAGCATTAGTGTAGGGATTTCGCATAGTACA<br>AAAATGAGCATATTTCGTAAGTCGATCAACCACCTTCTTAAATTAAGTTATTATTTTAAGTGG<br>TAGAAAATGAATGTTCTCTTATAAAATATTAAGTGTAAAAGTTCTCTCCTAATTCTAGAAAAA<br>AAATTATGATTGTTGATCTTCACCTATTGGAAGAAAAATGTTAGTAAAAGCCAATGGCTAGG<br>ATAAAGGAATCGAGAGTAGTCGACGATCATAAAGACCAAACCACTATAATTTTATTTTTTGT<br>AGGTGTCTTTCTCGATATTTGTTAACTTTGTTTCTGAATAACTTAAATAAGAACTTATTTTAAGT<br>TGAACTTAAGTAAAACTTGAGCTAGCTCAATAATCCTTGAGTTCAAATTCGAGCTTCAGCATG

	GTTCGATTGAATTAGGATCGACTTGGAATTAAACTTGAATTATGATTGAGCTAAATTAAAGTT<br>CGATTCAAACTTAGCCAGATCTTCCTATATCAAGAAACTTATTCCAAGTAGGGATATATATTT<br>CACAAAGAAAGAATATATTAAACGTTCTTGATTATAAGGGTCTCCTTGGTCCTTACATAGAGT<br>AAAATATGAAGCTTACTCCATAAGATATTGAACTCCTCAAAGATCCATCGTAAGATATAAGA<br>CTTATTGGGAGATTTATTTATCATACAATCACAAGGCCCGATATAATCTACTTCGGGCATCTTT<br>TTAGGAAACAACATCTTAGAATGCTATAACAAAAACTATTTTTTCAGCAAAGAATAATCTAA<br>ATATAACAAGGTATTATGATGCTAATTGAAGAATTTGTTCAACAATAAGAAGATCAATATAC<br>AAGTATTGCATTTTTTTTCTTAAGAATTCCCTATCTCTTGGTAGATAAAAATAATAATTAACTA<br>TTTCTCATTCTTCAGCCAAAGCATAACATTGCCAAGTTTTTTTTTTTTGTTTTTTACACCGCACT<br>TACAAACCCTTAAATAAAAGAGCTTGTTAGACATAAACTAACAAGTGGCAACTAAAAGAAA<br>ATGCTATGGGTTGCTCACCCCATTATGAAGCTAAAACTTTAGATTTATCAAGCTAATGAATGA<br>ATCTTGGATCTTTAACTTGCCAATATGGTCTTAGAAGTTACTGTCTCTGTTCTCTGTCAAAAAT<br>AAATATCCAAACCAGAGAACATGACTTTTGAATCATCAGCAAAGTACAGAAGAACCTGCAA<br>TTTCGCAACGATGCTATGCGCTGTAATAAGTTGACATCATGCTTGCTTATGCTCTGCAAGGTG<br>GACCAGCGGCATTTTTTTTAATTTTTCTCTTGAATCTGGGAATCACGTTAGGAGTAAGCAATA<br>ATAGCCAGATTGTGCCACGTGCCCAAATGATGGTTCATAAACGTATAAACCAACAAGCTATT<br>GTTATTAATTTAGAAACTGTTGTCGGTGGTAGCATGATTTACCAAATACATCAAGCTGAAGCT<br>TTTTCGCTGTGATGTCTGAGCTCACGTTCAGTGATATTAGAACTGCCGTGACTTATAAAAGCA<br>CAACCTAATAATAAAGAAACCAGGATAGGTGCGGTCTTCATCTGAGGCCACAGAGTCGTAA<br>GATATTTTGGTGTACTACTGTTTCCTTTATCACAAGAAAAAATCCACTTAAGTTGGTTGGATGT<br>GTAGATATTCTTCCATGATGACCCCACGTCATCTGATCCTGAGTCATCCAGCCGTCTGATGAG<br>ATAAAGATTGGGAGATATTTGATCGCTACCCATGAGCTACAGCACAAAAGTTAGTCACGGGA<br>CAAATAGGACATCGGAACGGCATGCCTCCTCAGTTCTCCCTTCCCCACATCGCCGTTCCCCCT<br>CCCTTCTCCTCGCTATAAATCCAGTGATTGAGCAAAACCAAACTCATCAAAGCTGCTTCTTCC<br>TTTCTGTCTGCATCGACATATAGTGAGAGTGCGAGAAATAGAGAGATCAGAAT
Ma01_g18020	ATGTCTCCAATTAGCTTCACCGTATAACAGGAGAGAGAGACAGAAGTAATGGTGTGCAGAG<br>GTGTGTGGTCGATTGGCTATTTATAGATGTCCTTGGATCGAAGAGTGCAGATTATTTATTGACT<br>TTGCAAAGAGAAGTCATTTAAATCTAATTCTAAAAGACCGGAATGACGGAGTCACGTCAATA<br>AATAATCTCACACACGTGATATTTTTTAAGTTATCCGTCTCGTAAGGCCGCAAAAGAGGACG<br>ATGCATCAAATGATTGAAATTAGGCCAATTTAATTAGGGGCCCATTAGATCTAAGTTAGGAC<br>CGAGTTGGGCCATCACTTGGAGTACTACCAGGGTGGGCGATAGAATAATTCAGAACTCCGAC<br>CATTTACCAAAAGTTCATATCATCGTAAACTAAACTTAACATTCCGAAATTTCAAACGAGAG<br>AGGTCTTTTAATACCTTTACACAATATATCAATTTTAAATCTGTCGATAACATTTCATTACATA<br>CAAAATATGCTTATACAACAACTACATAATAACCAATAAAACTTACCCAAAATCTGAATAGC<br>TCTCCACTACCTTAGCCCAATTCAAACATCCATATGAGTGATAAGCTAACCTGAAAGATTTAT<br>ATAACAACGGAGTGAGCTAAAAAGCTCAACAAGTGATAAAACATATCCAGAACGAAAAGG<br>AACAGTTTCAAACAAATAAGGTATCAAAATGCAAGGTAGGATATAATAATTTATAGATATAA<br>TCTTATGAGATACAGAATCGCAAATGTCATTTCAATTAAAACATATTTGGTTCATAATAAATG<br>GAATAAAGAGTAATTGGAACATATCAATGGCGTATAAAATGTTCCGGAGCATATCAACGAC<br>ATATGGAACATTTCGGAGCATATCAATGGCAAATAAAGCATTTTGGAGCATATCAATAGCGT<br>ATGAAATGTTTTAGAATATATCAATAACAATGAAACATTTCGGAATATATTAATGGTATATGA<br>AATATTTCAAAGCGTATCAATGGTGTATGAACATTTCAGAGCATATCAAAGAATAAATACAA

AACATAAGCTCAAACATCGTATTTGACGTTGCATTCATATCATTCTTACCCAAAGCACATATA
GAAGCACATAATCAAAGCTCTAGATATCATATCAAATACATAGAAGGTGTAGTGGGATCAC
AAACGGAATATAGTTCATCCATTTCTGGATGACCACTAGACAAAAATCTCCCATATTTGGGA
GCTCCATCCACCCACATCTGGGTGAAGTTAGAGGGGGGCCAACAGAGCATCGCAGACTCTA
AGCATAATCCCTTCATTTTTGGCAAAGGTCCAAATACTCTCACAAACACTAGAGTACAAAAG
GGCATTATGAATAATAAATTGACACATATAGGAGGCAAATGCAAAATAATGAAACATACAT
GTGCCTTTATAAGTCAATACAATGCAAACATAATCTTTCACAAATGCATTCAAATTTGAAAAG
AAATAAAGCAAGAGCATACAAGAATTTTGGCACATATCAGAAGCACATGCGAAACAACAA
AACATATATATATGCCTATACGAAACAATATGATACAACCCAAACTTTACATAATAAGTTTC
AAGAATATAAGTAATTTAAGAAAAAGATCATTAAAGAATACATGCTAGATGAAATAAATTTC
TAAAGGGAATGAAATAAGAACGGGCTAAATCGATCAAAGCTTGATTTTTGACAGAATTCGA
GAGACATATTGAACAAATAATTTAAACTATCAATTATGCTCCAATTCACTCAAGAAAGATAT
CATTGGAAAGATATTTTAATCTACTTTTAGATAAAATAAGTTTCATATGAATTAAAATTTCTA
ACATAGAGTTATAGATAATTTGATAACCAAAGAACAAGGAACAAAATCTCAGTTTTATAAAA
TTCATAGGGTCTGTTTCAATATATAACTAGGCATGCATTTAGAATCTAAATCTTTCAATCCATA
TATCAAAAGAAAGAG

CCAAATATTCCTCTTTATATTGCTTTGAAAATAAGTACAACTTCTATTGCACCACATGGCACTT
GCTACATGTTTGCATGTGACATGGGCATATCTCGATGTCTTTCCGATAAGGATGCACTTCCAT
GTGACATGCGCATATATCGATGTCATTAGATAAGGACTAAAATGCACAAACCAAATTATAAA
ATAAAAAAGGACCCACTTGGGGATTCAATCCAATAATTCACTTAGTTTACATGCATTACAAG
AATACACCATTTTAATTGCATTTGCATCTCACTTTGCATGACAAGATATCAAGCTGTGTTTGAC
ATCTGAATAGCTTTATTTTTTTTCTCTCCTATGTCGGAATGTCCCATGCAATTAGTTTATTGTTG
GTTTGATTGGGGAATCATTCAATATTGTAGAAGCTTGCACTGCGAGTTGTTCTTAGACAAACG
ATTATGATAAAAGATTGTCCTACCTATCTGCATGTTTGGTATGGGAATAGACTTCACATGGTT
CACAAGACATTGTACTAACCCATCTTTCTGGAATAGTATTCTCTGGAAAATTGTGTTCATAAG
AAAACAGTATTGGAATTTTTTCTGTACAATTACAGAGATTATGATTACCCAAAAACAAGACA
TCCATTTTCTAAAACAATATCTTTCTTGTTTATAAATTGTGAAAATAATTTTTCTGAGCTTTTGA
AGCATCTTACCAGATTGAATTTCTCAATAGAGACTCTCAATGTATTTCTTATGAATGGCTAAG
Ma06_g268 TATAAGTTAATTTCATCATGTATATTGATTACTCGATTGATCAACTATGGCTATAGCTCTATAT
70 TGTTGTGTTTGGCAAGATGGCTGCATAATTATCGACATAGCTTAAAACCATAGCAACTATACA
TTCTTTGATCTCTTGAAGACCTTAAGACTTTGTTGATACTATTCTTGTAAGGTAATTTCTTTTTT
ACTGAAATAATAGGAAAATTAAATTTTTTTATTTTTTAGGATTTTTTATATTTTTATAGTGAATT
AAATTATCTGATCAATGATGACCAGATGTGAGCCTAAGAATTTAAGTAATTTAAATATGCAA
ACAATTTTGTACCATTAAAGCATGTAGATTATTTTTGTCTTAAAAGAGCATGGTTTCAATGCA
ACATATGTGCGTTAAGACAGTCCAGACAAAAAATGTAATGCACAATGTATTAAGACAAAAA
AATCATCCCGATGATAAGTAATTTTTCTACCAATTTAGAAATGTGGTTAAAAGTCTAAGTTAA
CATAACACTTACTAAAATCGTTGCAATTAGTCATTTATAATAACAGTTTTATAAAGCGACAAT
CCCTTATCTTGTCTAAGATGAGTTTTGTTATAGTGACTACTCGAGCATATGCCAATAACATCGC
ATGTGTTCTCCTCAACTCGATCACTCTAGGTTTCTTTTAAGTGATATGATTACTATAATAGATA
GATAAAAATATTAACTTAAACTTTGTCAACTATCACAGCCAAACAAGCAGAAATAAAAAAA
GAAAATGTTCCTCATCACATTAGCTTAATGTGCCAAATAATTGATACATAGTCAGATGATAAC
AAAGGTGATAGCTGATAAAAAACAATAACAAAATAACAAAAGTGCATCCTCAGATCTGAT

CTATCTTCAAACTTCATTTACTTGTCCTCATTGAATTCTAAAAGTGCGTAGCAATCCGTGCTTT
CACATTAATTAAATCATACACTCCTATAAATCCGTTCAGGTCTTGGCTAAATCCACATTTCATT
AAATCAACGCAGCTTCTCCTCGTTCTTATCCTAATCTTTTATACACAGACGCTTCTTATATTAT
CCAAGTCTCTCTCATGGTGGGTAAGTTGAAACCCGTGCTTGTTCGACGCAGGCGGCTGCTCTG
CATCAATAAATACCACCTCCGCTGCAATCGATCTCACTGCAACGTTCACTCTCCAAGCTATTG
GTAGTCGCTATCCACTGTGTTCCAGGAAAAAGAGAACCGAAG
ATGTCTCCAATTAGCTTCACCGTATAACAGGAGAGAGAGACAGAAGGAATGGTGTGCTGAG
GTGTGTGGTGGATGGGCTATTTATAGATGTTCTTGGATCTAAGAGTTCAGATTATTTATTGACG
TGATGCAGTCTCTCCCTTTTCTTAAATCGATAATGCAAAGAGAAGTCATCTAAATCTAATTTTA
AAAGACCGAATGAGATGATACCCATGTGACATTTATTTGTCATATTACATCTACTGGAGAGA
CTGCGTCACGTCAATAAATAATCTGCACTCTTCGATCCAAGAACATCTATAAATTAAATCGAT
AATGCAAAGAGAACCCATCTAAATCTAATTCTAAAAGACTGGATGAGATGATACCCATATGA
CATTTATTTGTCTTATATCATCGACCGGTGGATTCAACATAAAGAAAGTTATCTGTCTCATAA
GTCAAAACGCATCGAAGTTTCGGAGCATGTGTCTGTTCACAAGGCTAGATTCGATTTTCCAAA
TATGCAATCACTATCACCAGTGGGTCATATTAAGGTCGTAAAAAGATAAAATGGGTGAATCC
AACACATGAGATGGAATCACACGTTAGGTATCGGCCATAGAGAAATAATCTTAACTCATTCT
CGAGCTATTTATCTCATTTACAAATGCATCTACAGAAAAATACGGTCATCATATATATATATA
TATATATATATATATATATATATATATATATATATATATATATATCATCCAAATCAAGAATATG
AAGTCAAAGTAACTCGTGGGATGCACGCCTTGCCAAACAAATACAAATGCATGAATGGAGT
CACGTTTCAACCACGTGCTTTGTTTATTTTCTGATTGGCTGGCATGTGGACCATGTAGAGACAC
TGGCTAATGAGAAGATAAATAGTCCACATTATCAGCTGCAATAAGTAGGTGCACCTGGGAGT
Ma01_g180 AATGCAGCACATAGGCAAATTATGGTGATGGTCCAAGAATGATGAACCCTTGATATATAAAA
40 GAAATGTCATTCATATACGATGAAGTCCAAAAGAATCAGATGATACATATATGTATATTACG
TTGATACAATAATTTAAATTTTTTGAGTTGTATTGATATTCAATCTCATGTATATTATATCTAAT
AGTATGACTGAAACTCACATGAAGATGATGTCCTCAAAATTATATGATCTCAATATTTTATAT
CTCAAAATGAGTTTTGATTGAGGTTCGTAAAATAAGTATATTAGATTAGTTCTTATCTATTATT
TTTTGTTATATGTGGTAAGATGAATTCTTTTTTTCTTTCTTCATCCTCTGGCTCTACTCTCAATGA
GATAAATAAATGATAAATCGATGCAAAAATATTATTTCTTTCCTAAATGGGTATATATATATA
TATATATGACGATGCATATGGAAGGATTTAATAATTATATTTAATATGAAATAAAAAAAAAA
GTTACTTTAAATTCGTTTTAATATCAATGAGTAATATTTTTTGGTGATGTAGAGAATTTGTCGG
GTCCCATTGACTAGCCATTCGAACGACACATTTCATTAGGGATACATGGATAGCATCCAACA
CATACCTCAATGACCGTTCATATTATTGTTTCGGTTTGTTCAACATATTTATACGAAGGCCACA
AAACGTATTGTATCTACGGGTTATCGTTTAATTATCATAATGCATAAATGATCTAACGGATCA
TATATTCAAGATTGATTACTCGACTTTATCATGATTTAGATTGACCACTTTCGTTAATGGCCTT
TTTAATTTACGCATATGATTTTTCTAATCACCAGATAGCAAAGTGTCATCCTGTATTATATTAT
TAGATTAAATAACACATAATAAAAAGTGGTAATATCCATTTTTTTTCCTCTATGACTACGTA
AATAATCTATCAAAATATATTACTTTATAAAACCCTAGTGATGCCATCTCGTTCCTCCATCCTT
AAGATACTTGTATGCAAAAAGATCGATTATGTGAAGAAACAAAAGA
TCCCGGAGGCCAAAGTCCACAACCACAAGTGAAGAAAGGAGGGAACTTTACAAGATCTAGT
Ma06_g301 GAATGTATATATCAGTCTCAGATAAGAAAAATCAAAAAGGAGGAAGCAGAAGACGATGAA
70 GATGAAGATGAAGATGATGATGAGGAGGACCGAGGAACCGTCGAGCGGAACAGAGAGAGA
GATGGCGATCGCAGTGGGTGTTTGATGGAGGGGAATTATATATTTGTAGCAGGACAGTATGTT

GATGGAGAGTACACGCGAAGGTTCGCATGGAGATTAATGCATGTGAAGGTTTGTATATTTGC
AAGGTTTTGAATTTTCAAAAACTTTTATGTTAAACTGAATTATAAAGAATCACATACAAAAAA
TCATTTATTTTGGGGATGTATATATGAATTTTTGCATGCTTTTGCATGTAAGATTTTGAGTTAGG
CTAATTAGATTTTTATATATTTATAAAAATATTTTATTTTTTTTCTCATTGATTTTCTTAATATAA
ATACATATATTCTAATATAATTTTTAAAAATATAAATATCGTAACATTAAAATAATTAATTAC
AGAAATTAATACATAATTAGCCCTGTTGAGTCGGCATCAGCTTCGAGCAGCTCCAATGTGTCG
GTCGAGTGACCAAACACGTAGTAGGGAACTCTCATTGGCCTCACAGCAGCGCCGTTGGGTCT
TTCCATGGGACCCGCGACTGCTGACCAATGATAAGTGCCACGTGGCATATATGGTGGCTTGC
CTGGCCCATAGGATCCGTTTCCCACGTGACGTTTCAAATCGAAAAAGGTCCTTAGAAAGCTT
ATTTGTCGATGATATATATATATATATATAACGCACAGAAATTATTATTTATAGGTGGAAAAA
GGCACGTGCGTAAGACTATTTGCACGTGTAGACACATCTAATTAAGGGAAAAAAAGGCTATG
ATTAGGTGTATTTCTTAGAATTTTACTTAGGAAGGACTTAAACAACGAGATACTTGTCATATT
AAAAAGGAATAAAGGTAGGACTCAGATATAACAATAATAGGTAGGAAATCCAAATGGTATT
TTCGATATCTTCATAAAAATGTTTCTTTTAGCCACTTGGTTTCCTGGGAATATCTATATTTGCCC
TTTTCATCAGTACTTACCCCATGCAAATGCTTTGGCTGACCCACTTGATTGCAGAACAAACTT
GACCGCCTGGTGCAAGTCATCTCCGAGCTTTCCCAGTCCTTTCAAGATGACTAGTGCCACTCA
TTTTTCTGTTCTTGAGATTGTTCATTGTGGTCATCATCTCACCTTTTCCTCTCAGAAATGATGCA
GGAGAAATAAATACTGGAATTCACTGATTGAACAACGACCTAAGTTCATACTTGGATATGCT
CCTTTTATTTTAGCATGAAATAATGATATCTAATGCAAACTGTGTGGGGAAATGATTGTGCGT
ATTATTCACAGAGAAGGCTGTGAGATTGCATTGCTCCGATCATCTACAGTAAGTATTCCTCAA
GTACTCAGACCCCCTTCTACGTGAACAGCACCTGCAGTTGGGACAGATAACTTTAAGCTCTCA
GGCCTATTGCAGCACTACCTTTCTGCAGCCAAAGCCATGGAGGCTTAAAGGAGTCTGATGCT
AATTGCTCAGCCGAGGATGGATGGATGGATAGAAGCATGATGTGGTGAGAAAGCAAATGAC
GCAAAACTATGTTACATCAATTGGAAGATGTCTTACTTTATGGTGAGATCATTTCAGAAAAAT
GATGGCCAGCAGAACAATGTCAGATGTGGTCTGAGTTATCTTTACATGTCTAATGATTGCAAG
CAATGGACCCACAATCAGGTATAGAACTCATAATTCAGCATGCCATTTGTCAATGCTGATAA
TGTTAATAACAGTAATATTATACGAAACTTTTGCAAGTGACGCATGATGTGTAGTACAAGAA
CAGCAACATAACCTGTTATAGTCCTTCCTGATCATTAAATAATGGAAATAAGAGTTGTCG
TATTCTAAAGATAAGAGAGGGCGGTGAAGGTTTCGCAACTGCGTCGAAGACGATTGGCTTAC
GGTGTATATAAAGACGAACTTGCGTGGTGGAACAGTGGGCTTAGCTTAACCCATACGACATA
TGTGCGTGTCCGAAGTCAAACAGGGATTTCGTACGAAAGTAAGGTTGGGGAACACATTTAAA
ATGCAAATATATTTGAAACATGTGTCGAGGGTTTGTCCGGTGAGAATACATTTCAAAGGTGTA
TCGACGTCTAATATATATTTTAAATATGAATTGATATTTAGTATTTTTTTAAAAATCAAACTTG
CTTCGATGTTATATTTATTATCTAAAATTATAAGTTTGTCAAATTTAGTAAGAAACAAATAAA
Ma09_g120 ATTCATGTCTTTGTAGAATTTTATTTATTTATTTTTAAAATTATTTATATTCATTTATATTATTTT
90 AATTTATATTTATTATATATCTTGTCCATACAAATTTTTTTTATAAGATTATATTTATTTATTAG
AAGGATAAATTTATCATATTATATTTAATATATTTTTAAAATATAATTTTATCAAATAATACTT
ACATATATATTTCTATTATTTATCGAATGCTCAAAACATTTCATACTTACATATTCACTCAAAC
TTATTTTCAGCCTAACATCATCATCTTATCCCATCATTTCTAATGTGGATCTTCGGAAAAAAAG
AAAAAAAAAAAGTCAGGTTTGTTCAACTATGTAATGTGGCTCAACAATAATATTATAGTAAA
AAAACCTAATCATGCAGAGGAATTTAATGACAGAAAATTCAAATAGAGATTGGATAGTTGGT
ATGGTTGCCCCAAAAACCACGTAAACGGTTGACAATTTGGCTTCAAGCAGACCGACACCGAT

AGATTTCGGTTCCCATTGCTATTCCTAAACGTGAAGTCCAAATCCTCCATCAACACAGGAAA
ACGAAAATGGCGGTAGGAGTCCAAATCCTCCATCAACATATGTCACGTGTTCTCAAACTTATT
GAGACTGCTGAGTTGTCGTGCCACGTCTTATCGAAGCTCCAGTGATTTCAACGATCAATCATC
ATAACGTGTAAGACTACTGAGTGTTATTTTTGGGTTGGGACTCCGTACGGTTCACTAACAAAG
CAAATTCCACGTGTAAAAGCCTTCAGCCTTCAGCCTTGGTGTTTGACTCGTCGTAGATGTGAT
GATCCATTTCGAGCGCGTTACTATTTCCTTAAACGGATATATATATATATATATATATATATAT
ATATATATATATATATATATATATATATATATCAATTCCATGCGAGGTTACGTAAAGATATAT
CGAAAACTCAAAGTCTCCACCTCAAGCATGCTACTGTTTCTCCAGACTTGTTGGCACAAACTA
CGTGGCATGATGGAAACCATACTGACTTCCACCAAACGTGAGGATATCTTTTACGTTACATCC
TTAATTTATGATGATTGTGAACCAATCACCGCAAAAGCAATTTCAAGTGTTCATCTTTTTTTTT
AATACAATAACTACTTCCTTATACACTTATGCACGAGATATTCTTTCGATGTTGCCTTCTCCGA
TCTAAGATATGTGATAGCTTGACTTAAAACAAACTATAGTAACACATGCAGATAAATAAATC
TTAAAAAATTTTGACATGCCATAAAATAACACTCAATACATGAATATTAACCCCAGTTTCAA
ACGAAATATGAGTTAAGAAGCACTACAAAAATCGAGTCCATAATGGATATCGTTTTTATATTT
CGACTTTCTTTGTTATGAATAAACTTCTATCAGCCTAGTAGATGAGCTAAAAAATAAGTTAAT
TATCTTTCTCCGATTATAATCTTATTAAATTATTACGATTATATTAAGTCATGCTCATATCATCA
AATAATAGAGAATGGATCGATCTTTTTCTATTGATATTATTATTTACTTGTATTATAAAAAAAT
CATTAAAACACCAACAAATACAAATTAATTTTTTCTAATTTT
ATTGCATGACGATCGTTGGGAGATCTTGACAACCATAATGTCAACTAGAATAATTAATTATTT
ATGTGCACCGTACAAGGAAATTAAACTATTTCGTTTAAATCCACAGGCAAATGTAAAATAGA
AAGAAAAGTCTTTGTTTATATCGCGTTTTGGTGGAAAAGTCTTCGTTCCTATTTGACATGAGCC
ATGCTATGCTCTCGTTTGTTTATCGTAGACAATGCCGTGATGTCATTGTAATTGTTCTCCAGTCT
TTGGTTTTGACTTTTTCTCTAGCAAGAATCAAGTTATGTTAATCCAATGTAATATATAGCCAAG
TTTATGCTGTCATAGATGGATTCGTTGAAAATGCTTTATGTTTCTCGCTTGATGTTAAAGAATT
TGTTCTATTGAAATCATCTTAAAAAATTTATATTCCTCTGAAAGAATTGGCTACGTGTGCATGT
TCATGTGGTGATTAACAAAAATGCTGATAATTAATGTATATATATATATGAACAATTAACACG
AAATTATCGTTAAAAATATTGATTGATTTGTGAAAGATGAAGAATGATGGACTAAGAGAATA
GTATCACATTGTTATGGATTGGAAAAGTCCCATTGGAATTTGATCCAAAATCTAAAAGTTTAA
ATGTTCTAGTTGAATTTTTATCACACTTGATGTATATTATTGGTCTTTGAATCAACTAAAGCTTT
Ma08_g234 ATAGGAACAAACTATATTTTAATATCAAGAGAATCGGACTTTGTCTTCCATGGCCTCCCATGG
00 AAGAAGATTCAACAGCCAGCTATGGAAGAATGGAAAGTTTCCTCTGAAATGACCTCAACTCC
ATTATCATTTTACCTACCTAAGGATATCTATTCACATCAACATCTCTCTCTCTCTCTCTCTCT
CTCTCTCTCTCTCTCTATATATATATATATATATATATATATATATAGTGACAGTGGAAGGA
GTACAATAATAAGAGGATAGTGGCGCGGCCATTAGTGGGGATGGTTGAAAGAGACATGATG
TATGCAACCAAATAATAAGTTGATCCATTTCTTGCAAGTAATGTGCAAATTATATGGAATTCA
ATCATCATTGTTGGGATATTTTATTTTTTGCTTTAATAATTTATCAACTTAATATGGGAGGGAC
CATTGCATGCTCCTACCATAAGTTCCAAACGAGTTGGAAGTATGTACGTGTTTGGATTCTTAG
ATGATTCCCTTTTGGAAAAATATGGATGGGATCCGATCAAAATTAATATTGATAAATATTTAT
ATATTTTTTATTATTAAATAAATCACCTTTGCATAAATGGTAGAAAATGATATAAATATTCATT
TTCCCATTGGCGTCGGATTGGGCTGTGCTCTTATTCCGGTCAACGCGGATCTCCGTCCAAATTA
ACGAATGGCTTCGAGCGCAAAGCATTTGCACGTTAAACAGGCGGCTAATCAACGACCTCAA
ACTTCCATGCCCATTCTACCCTCCCTTTCCCCCATAATGACACGACATCAAACAGAGGCAACC

	AATGGTGGATGATCAATGTCTCGGTCAATTCCTCCGTAGACAGTTGCCAAAAGGATAATTCTT<br>TCTTAGTATCAGTCGTAAAATATTCTTTGAATTGACAAAAAAGCCACATTTTATATGTCTTTAT<br>GTATTAGTCGTAGAGGGACCGAATTGGTATTTTGTTCGCAGCGAGGGCATCGGCGGTCATTTT<br>AACCGATTACAACGTCCATCATTGCCGCGAAGCAGCCCATCTCGCCTTCTTTATGGCAACCGG<br>GAAAAACGTGTGGCCGGCGCCCTAAAAGCGCAAGGATTTCAAGCCCCTGTTTTACCACACCC<br>CCCTCCCCCCCTTCTATCGTCCTATAAGAAGCAACGACTCATCCCTCTTCTCCCCCCAAAAGG<br>AAGGATAGAAGACCCCCAGCCCTCCTCTGCTTCTCCAAATTCCCTTTGTTTTCGTAACCAAGC<br>TCGAGTTTGGAGATAGACGAAGACGCACTCGCTCGCTTCCA
Ma02_g07800	ATTTTCTCGATCTTTTTGCTTGATTTCTACCGATTGGTCTCTTTGCAGCTCTCCCAAGTAATCCT<br>CAGCTTATATGGACGCAATGAGCTTTAAGGATCGATTGTTCTTCAAGAAAGATGAACACATG<br>ACTCCCAAGGATAAAGCCAAAGAAGGATTATGCTACCTTTATCTTTACTCTTGCATCAGATTC<br>TTAGACTTGCTGTGGTTCGAGTTCAGGAATAAAGACATTGCGTTCTTAATCTGCAGACAGTGA<br>GATTAGTACTGCAAATGGACGTATAACACAGAAAACTGATTGCTTGATTAGGTCTGCATGGC<br>TGATGCAGATCATGTGGTCCAACATGCAATTAGACGAAGACTCAGAAATCTCACCTATTTGC<br>ATTGAGACTGATACAAAAGGGAGAATAAACAGTTGGAGGGAACAAGAAACGCTGCAGCGTT<br>CATCAACAGATCAAAGATTCCTCCGACTTTTCGAATATTAAAGCACCGAAAGGTACAAGCTT<br>GCTTGCAGTGAGAGCTTCTGAGAAATATAGTTTCATAGAAAAATAGACAAAATTGAAGAAA<br>AATAAGCATGCAACGCATTGATTGTGATAATTTAAATCAGTTAATTAGGATCAAGAAGGTTG<br>CTTCCATTGCAGCAAAAATCTACTATTCTGGTGGAAATTAAATTAAGCTAAAAAAAAAGGGA<br>TGATGCCAAAATTCGGCTGTTCATCTCTCGCTCTCTTTTTTTTTTTTTTTGATAAAAGGTAATT<br>ATGATATTCGAACCTAAAATCTTATGATAAATTGTTAAAAATTTTATTAACTAAATTAATTAG<br>AATCTTCTATAATATAAAATAATATTATAAAAAATAATATTGTAAATAGGTGGTATGGGTCGC<br>GCGGATGATGCGGGCCCCACCATGATATTTTATCCACGCGTGCAGGTGGAGTGGGTTGAGTC<br>AGCTTTAAATGCTGTGGTTTATGATATCTTTGGTAGTCAATTTTGGAGCTCATTTCTCGCCGAA<br>ATCTTATTGCTTCTCCTTCTTCTTCTTCTTCTTCTTCTTCTTCTTCTTCTTCTTCTTCTTCTTCTTCTT<br>CTTCTTCTTCTTCTTCTTCTTCTTCTTCTTCTTCTTCTTCTTCTTCTTCTTCTTCTTCTTCTTCTTCTT<br>CTTCTTCTTCTTCTTCTTCTTCTTCTTCTTCTTCTTCTTCTTCTTCTTCTTCTTCTTCTTCTTCTTCTT<br>CTTCTTCTTCTTCTTCTTCTTCTTCTTCTTCTTCTTCTTCTTCTTCTTCTTCTTCTTCTTCTTCTTCTT<br>CTTCTTCTTCTTCTTCTTCTTCTTCTTCTTCTTCTTCTTCTTCTTCTTCTTCTTCTTCTTCTTCTTCTT<br>CTTCTTCTTCTTCTTCTTCTTCTTCTTCTTCTTCTTCTTCTTCTTCTTCTTCTTCTTCTTCTTCTTCTT<br>CTTCTTCTTCTTCTTCTTCTTCTTCTTCTTCTTCTTCTTCTTCTTCTTCTTCTTCTTCTTCTTCTTCTT<br>CTTCTTCTTCTTCTTCTTCTTCTTCTTCTTCTTCTTCTTCTTCTTCTTCTTCTTCTTCTTCTTCTTCTT<br>CTTCTTCTTCTTCTTCTTCTTCTTCTTCTTCTTCTTCTTCTTCTTCTTCTTCTTCTTCTTCTTCTTCTT<br>CTTCTTCTTCTTCTTCTTCTTCTTCTTCTTCTTCTTCTTCTTCTTCTTCTTCTTCTTCTTCTTCTTCTT<br>CTTCTTCTTCTTCTTCTTCTTCTTCTTCTTCTTCTTCTTCTTCTTCTTCTTCTTCTTCTTCTTCTTCTT<br>CTTCTTCTTCTTCTTCTTCTTCTTCTTCTTCTTCTTCTTCTTCTTCTTCTTCTTCTTCTTCTTCTTCTT<br>CTTCTTCTTCTTCTTCTTCTTCTTCTTCTTCTTCTTCTTCTTCTTCTTCTTCTTCTTCTTCTTCTTCTT<br>CTTCTTCTTCTTCTTCTTCTTCTTCTTCTTCTTCTTCTTCTTCTTCTTCTTCTTCTTCTTCTTCTTCTT<br>CTTCTTCTTCTTCTTCTTCTTCTTCTTCTT
Ma07_g09040	GAAGCACAGATATGTATGTATATGGAGAGAGAGAGTAGCGAGTGGACCGTGACGAAATGGG<br>AGAGCGATGGCTACACTTTTATAGAGGGGATCAAATGGGATGTGGCAGCAAAAGATGGCAA<br>AACACTGTGCATGATGGGGTCGACACCAGTGGATTTCTGCAATGATCCATTGGTCGGATTCA

TGGTGGAGTATAATCTGGAACAACTTCACACTATCAACAGCGAACATCCAAAGCACAAAGG
AAATTAAGATTCATTGACGCAAAAATCTTTGGTTCTTTTGGTAGGGTTCCTCGTTCCATGATGC
TGCGACTGTGGTTAGCTACGAGATGATTGGCCTATTCAAGAAAGTGGCATGGAAAACTCACA
TGTATCGTTCATAGTGGTGATGGAATGATTGAAAGATAGCACCTCCGAGTATCATGATTGAG
GAATCTTGAAACTAGTACAACTCTACCTATTTCTTCGATTGTAGGCAATGATTAACTCTAATTT
CAATAAGAAATTGATTATATTTTTGCAATGTCTATATCTAATTGGACTGATCTAATAAATTACT
TATTAATTTTATTAATCTAAGTTACTAGCATAACATATCTATTGGACTAAGTTTAAGAGTGTTA
ATCTCTCCTACTTATAACTTCAAGCATAAACACATTCTAATATAATACACATAGGTTGAGTGA
TGACTTCATGATGTGATGTTTCATCTGACTTGGTACATAGATAGTCAATTGATACTCAAACCTC
TCCTCACATCTAATGCTAGATCATTATGATATTATTTACCATGTTCTTACTTGATTCGATAATTG
ACTTATTAATTTTGTTAGCTTTGATTGATATTATTACTCTGATCAAACACTTATGGGCTAATTG
AAGTATTAATGAACTTTTGATATTATTATGTACCACTTTAACTAAACTAGGTTAGAGAAATAT
TCTTCCATTTTAATATGATCTCTATATCTATAACATTTCGATTAGTCTCATAGATAAACTAATA
TTGAGTTATAAGGGTTTGATAAGTATATTATTGTTAACTTTAGTTTAGATATCTTAAACTAATG
GTTTAGACTCAACAAGGTTAACAGAGCAATCATCCCAGGGTTAATATTTAGAAGTAGAGATG
ATAAAGGATAGGAGTTGACTATACTTGACTAAGTTCTAATTGGGTTAAAGAAGTTTCATTCAC
ATATTATTCATATATTTAAGATTATATTAGGTAAATTCAATGTTCAAAATATATACCATAAGA
AAAAGATAATTTTCCCGAAATAAATAGCAATATGTGATAGAAACCAAACAAAATGATGGTA
AGATTGGAGACGAGAGAGAGAGAGAGAGAGAGAGCAAAAAAAGATAATAGAAGGTATGTT
AAAGAGTGATCAAAACTTAATATCAATTGTGCGAGCATTTAGATTTAGATTGCGGTCATCAAT
GTTTCTTACTTCTTCCTTCCAAGCTTCTTGTTCATCTTCTAAGTTGGAATACTTCTGTAGAGAAG
CTTTGCTCACCATAAATCGACACTCTACAGCTACATGTTTTAGTAAGACTTATCATGCTTGGC
AACAGCAGTGGTATACACTTACCACTGTAGTAATGCTTGTCACTTGTAATCAAACAAACACA
AGTGTGAATTAAACAATTGCCACTTTGCGCACCTCCACTTCCATAGTTGAAACGTGACCCATT
GATTCTCCACTCTACAGCTGAGGCTGAGTGAGATCACAAAACATCCGCTGACAAAATTGAGA
GGATTCAGAATAGTTTTATGGTGCTACTAATTGGAAGAGTTTGGTGAAAGAATTCTGATGATG
TATATTGATTAGGATTAGGATTTAGAGACGTACATAAATTGCTGTTGTAGTATAATTCTGATGT
ATTAAATTACTGAGCGTGTTAAAGTGTTCATCGAATACCATTCGCTCCGAGTTTTGGTCAAGA
CGAAACTGGAATTGCCATTAATGCATCATCAAAGCGATGTACAGCAAAAAG

Ma10_g25490

CAAAAGATTTTCTCCAAACACAAACAAAACACTCAAATATCAGTATAAGACATTTACAGGTT
ACCGACATTATACCAATAATTAAACTCAATTCCTGCTGTACTACATGAAATGTTAATTAAGCA
TAATGGTTACGCAAACCATGGCCACGCATATATATATATAGATAGATCCTTTGGCTGTTTCAA
GGATCCATCTTCTCCAAGTTCAATCAGAAAGTAACTTGGGATACTTAACAAGTTATGCTTGAA
ACTTACATGAGCAAAAAATAAAAAAGAGAAAAAACTGGATGTAGCAACCGACATGTGCAA
CTTAGTGGAGCACAGACTTCAAATTATTAGAAGTGAAGAACAGATAAGACAATGAATAACC
TACTTTTTGTTCGCTGTTTATCCTACAACAGGTATCATGATATATGAATGTTATATATAACAAC
AATCTATCTTCTTTGAGACATATATAGGCGACACCATCATTTGTGTTCATGTAAAATCAATGTC
ATAGATTAGTTTAATTACTTTTATTTTAGAAGAAGAAGTTTAATTACTATGGGGCCAGAATCT
CTCATATTCATGCAGTCAAAGTTACAAATATATGACATTAGCTTCCATCACCTGTAATTGCAT
CTATCTGGGGGCTCACGTTAGATTACTAAATATCCAACATGTCCCTTCATTTTTGGTGGTCCTT
TATCGCTATGCTCCTACCCATCCCCACCCTTCTTCTGCCCTATCACTTAAGACTCTAACTTGCA
GGCTTGCAGCAGTTAAAGACTTCTTGACAAATCTTGTGGTTATTCATGTTTCAAGGAAGCTAT

GCCCATATCTGATAGCTCTGAGCCCCAAAAATTCTAGCAAAGCACATTACCACTTGGCTATC
ACGTGATGCATAAAAAGATTCCCCCGACTTGAGGAAACAGAGATTCAATGGCTGAAAGATT
AGGAGTTACTCCCGTCGCTTAGAACCAGATTGATGCGCTAATAGTATATGGCCATATTGCAG
AATTTGATTAGTCCACCCAAGGAACAATATGACAGGCTAAAGGCCAAGAATTTGGGGGAAA
AAAAAGGAACAAATTGTCCTATCAAACAAGCCCTAACGGCGAAGTAGAGCGAAGGAGACA
TTCTCGAGGATTTCCAAGTAATCGTCCATAAGCTGTTGATGAAATTATGAGAGAATAAACGA
TCAAGACAAGAAATCCCTTGTTTCCACTCTGTTGGTCTAAAACGATCGTAAAAGAAGAACGA
AAGACAAGAGGAACTTGGTAAAGTTGTAATACCTGGTTCTTGGGGAGGAAACGGAGGTGGA
AGAGGAGGAAGAACGACCATCCAATCCCTCATCTGGGTTAGAGCACTATTCAAGAACCAGA
AACCAATCAACAAAACGATGTTCCTTTGATCTGCTCATGATACTTCATGCAGGAACAATATGT
CAAGATCTCTCTCTCTCTCTCTCTCATGCTTTCTTGATTCCACCGCCGTTGATTTCCTACACC
TCTTTGGTGTTAGAGACCGATTCAAGAACCAGAGACCCTGCAATAAGAGTATGTTCTTTACGG
ATCGAAGAACAATTAGTAGGGCGACGCATCCTGTTTCCGTCGAAGAACGATTTCCGGCGATC
GCCACGGCGCCTGGTCTCCTCCTTGCTTTCTTTTATTCCACCGCCCACATGAATCGTTCCGCCT
ATTGTTTTGTCGTTTTCCTTCTTCCGTTTTTGAGTCGTAGCTTTCGTCGTGGGTAGGTTATATATA
TATATATGGAGGATCGAGTGCAAGTCTTTTCCTTGGAAGTTGATTCGGTGGCAGTAGGTGAAT
GCGCCACAAAAACCATGCTCATTCACGAGACAGATCGGACAAAATCCAGCACATCGTCGTC
AGTGTCCCCCGGCAAAATACAAAGCCAGCACAAGTCCTCCGAGTTCACCACTTGCCCTCCCA
TCCGCCGGACTCCTCATCTCTTGCCTTCTCGCGCGTGAGTTCGGGCGTCAAACCCAATCGGGA
CCAATATCGTCCCCTTCCCAAAATCTCAACTTCCCGACGGAAACAGTAGATTTTTGGGCAGA
AGCGCAAGACTTGGTTTCCCGTCCGCTTGAATTCCTCCCCTTCTTTGGTGACTCTCCTTGGTCG
AACAGGGGAAGGTGGGGCAAGTCCTATATAGGGGCTGGCGAGTGGGTAAAATGGGCATTGC
ACACGGACACGCTTTCTCCCTCGCGATAAATAAGGGCCAAATGGCAGCTTCGCGGCAACGAC
GTCCGTTTCAAAGGACCGCCGATGCCCACCACAAATCCTAAAATACCAATCCAGTCCCGTAA
GCATGACGGAACACAAAACAGAACATGTAGAAATCGTATGGGTTTCCATTATCCCAAGTGTA
AAAGGATACTATGATTTATTTAGCTCGGAAAAATCTTAGCTTATTTATTTACCTCCCAAAAAA
ATCAGATAGATCGGATTTCATCTACGTATTTGACCCCCACGGCTTGAACCGAAATTGAACCG
GTCCAAAATCAAAATTGAATCGAGTAGATAGAATGATTTCGAACCGAACCCAAACGGATTC
GATCTGATGCCAATTGGGTGAAATTGCCAGATATTCCTGAATATTCCACGTAAGCAGTAATCA
ATCATTGCTGGTGTTCCGCACAAGTAACTCGTTGTTCACACCGTGCTGACGTGGCCAAGAAAA

Ma09_g15420

AGAGGAAAAAGTATACATAACAAAGTAGCTTTGGGAGCGTGTGATGGTGGCCACGTCGCCTC
TCACCCACTGAGTTAATGGGGGAGACCACTGTCATATGGTGAAAGAATGAGGGCATGCGAA
CTTGGTGGGGTCCGAATGAATAGGGCGCCCCCACTGCTCCACGTGCGAATGGTTTTTTCCTTC
GTGCGATTCCTTCAATTTGAACGGAGATCCGTGTTGACCCACATCCGCCACACGTGTGGTCAA
ACACCTAAACGCATGTAACGCAGCCCAATTCGCCACACCGTCCTCCGACGCTCGGATGGGAT
TCCGACGTCATAATAATAATAATTAATTGATGGATATCAAATAACCGATGGGAGGACAACAA
TTTGGGTGGGTTTATAAAGGGCATAATAACCCACATGATTCCAAAGCTAAGGGAATAGGAAG
CCACGTGTATCGAACTTATTAGCTGTTGCGTAGGTATGTGAAAGCAGCAACAGTGGATCAAA
CAAAATAAGACAACGAGCTCTTCGTTGGAGGAATAGTTCGTCGCTAACGTTGACTTTAGAAC
TTGAGGTTTATTGGGATCCTTTCGTCCCTTACTGGAAGGAAAGAGATGGAAGTTCTGTGCTAT
CCTCACAAAAATGAAAGGACTGTCTTAATCGCCACACTACTTTTTTCTCTACGCTCTTACATT
ATTAATCTGAGCAGTACAGAAGAAGAAACAAAGTAAGAAAAGAAGGGAGATCGCGTGTTCT

ACCTGTCTTCTATGTATCTATGTGTATATAACAAAGAAGAGAGGAGGCGGAGGAGGAGGAG
GAGGAAGGAAAGCAGCTACTAATTTGGAGACCAATGCAGCAGCACCGCCCTCCCTTTTCAAG
GGCAAAAGAAGCTAAAAACCAGAATAAAAGGTTCGATTTCAATTTTAAGAAATTTAAACTG
GATTCGATTTTGATTCGGTTCTCGCTCGAATGAATCAACCATGAAACTCGAGTTTGAATTATG
GAGCTCTAGAAAGTCATGTGTTCACATAAAAACCTGCCTAACGAATTGACTGGTTAGTCGCG
TTGCTTTCATCCGCCATGGACGACCGGCAGCTGTGTACGAGTGTGGGATGCATACTTCGCCA
AGGCTAATATCAAACATAATTGATGTTGCACTTCGTTTTATGTATTAGTATGGTTTATGGGTTG
CATATATGTCTTCTTTAATGCCTTGTAAATAAACCCCTTGTTCTATGGTAGCGTAGAATTGTCA
AATCGGAACCAATTGGTCAACGCAACTGTTCGATAAAGCAGTCGGCAAAAGATATATGATAT
AAAGTCC

TTCCCTCCCTTCTTTTCTTCCTAAAGATCAAAGTAGTCACTCGAGCAAAGTTTCAGGAGAAAG
AAGGGGGGGGGGGGAAGCTTTCTTCTTCTTCCTGCTCTGTTTTGTTGCGACAACAATTATTGC
GGTGTGATCTAAGGATGGGTTGGCAAATATAAGAAGGGAGGGGTGGCAGGCGGCCCCTTGG
TTTCTACTTTTCTACCTCGTGCAGGTTGCTTGTTTGATTTCTTGGCGTTTATAGCTCATAAACAC
ACGCTGTATGGGTGTGATCGATTGCGTGATGGTAGAACAAAGTCAACCGTGAGGAAAAGAA
GGATGTTTCCAGGTAGTGGGGTTCATTTCTGCAGCTCGAAGGACCGGCATGCCCATCTGTGG
TGTTCAAATGGCATATCAATGGCAGAAGCAGTACTACTTTCGCACTGAATCCCGCAACTCGA
TCATTGGAACATGATGGGGATGATTTCCTCATCGTAGGTACTTCATAAGGACAGGAGGAGAT
ATCAAACTTGTTTGTTTGATTCTTGCTATCATATTTTTAGTTGCTCACAGGACAACATCAATAG
AATTCAATCTTGCTTGGTGTGATTGTTGAGTTCGATCTGCCAAGTCAATTTCTCCAATAAAGGG
ACATGGCGATCTCGTGACGTTCAAGTAAAAAAACGAATTCGTGGTGTTATTTTTTATGACATA
AAACTTATCATTTATTATCTCAACCACAAACTAATGAGTGGTGTAAATGCCACTTACTATCTT
CTTTTCAATCATTTAAATACCGAAATTTCTCTATAATAATTGAAGTCATCGGAAGTCTAATTCT
TTAATGACTCGCCTAATTAGCTTCCCGTTGTTGTCACCCAAATATTCGATTTAAAGGGAGAAG
ACTATGCACTCTAATCCTTTTTCATACATTTCTTGTATGACAATGAGTGATAGTTAATTTCGTG

Ma10_g17560

AGTAGTATTTGAAAATATGAAATTGAAAGTGAAACGAGGATATTCGTCTTCGTCTTCGTCTTC
GTCTTCGTCGATGCATAGCTAGCACAAATAATAAAAGCTTAACCAAAGGACGCATCATGTCG
ACATAATCATCGGAACAAAAAATATTCCATGGATATTTTTAATATAATTTTTTTTTATAATC
ATGAGTGATTTAAAATAATGATGAACTATAAAAGGTGAATGCAAATATAATTACAGAGGTCG
CACGTTAGGAACCCTTCTAGATGTTGATCTTCATGCGTGGGAAGAGTGCTCTCTTTGTTTTTTT
GTAAATATAAAGTGTTTTGGCTTAATTTATTAGTTTTGTTTAGACTGACTTTGATGGTTGATAG
AAAAATTATAGCTCAAATCTTGTCTTTACCACTTGTCACTTGCAAATTATTTTTTCTTATTCTGT
TCTTCTCTGGAATGTAACATTTCTAATGATTGATGCTCAGACAATCTCTTATCCTTTAAATATA
GTAACATCGGTAAAAAAAAACAGAAAATATTTTATTCATATTATAATCTATTTTTTTTTATCCA
ATCTATTATAAAATTGATTAATTTATCATTCTTAATAACCTTACTTTTCATATAATCCTAAATTA
AAAGAGAAATTGAATCGAACATCCGATTTTAGCAGAAACTTTTGATGGTGATAGATGATCTT
AGATCTTTTGTTATGTGACTGTTCAGAGTTTAAGAAATTTATTTTTATAATTTATATTATCTATC
AAGTGTTGACTAAAATAATTGTTCATAGAATCTCGAGTTAAACTCGTCCGGTGCTAAAGGATC
ATAAAATATTTTGAGAAAATTAATCGCTGAACATAAATGATGCTGTACGATTCAGACAAAAT
TAACTAGATCTATGACTGACGTGTAATTATTTTCAGATTGTTACACATGCAATCTATGATTAA
GACGGATCGATCGAGTCGTCCCTGACAAACACACCTTCAAATGTGTCTCTATGTCAAAGGAA
ACAAAGCTGTAGAGACGACCGCCCTCGCTTTTGTTCTTGAT

TTCCGAGATGAACACAAGGGAGGGGATGGAGAGGGAAGAGGCTCTCTCAGAGACGCTCTCA
GAAGTGAGTGCTGGCTTTGGATTTTGCTGGGAGATGGGTTCTGATATGGGGGAAAGCAGAGA
TGCGAGGGCAGTGTGCTCTAGTTTTGTCCGATTTGTCTCGTGACTCTCCCGCATTCAGTGGCTC
ACCTACTGCCACCCTATCCTATCCTTTGTTTTGTCTGCTATAGGGGAGACGTGGATGCACCGG
CATTTGTTGATGCATCAAAATCAGTCTGCCGGTGGGTCAAAATCTCATCTTCTACCAGCACGT
GAAACACAGTGGTTTGGTTTCAACGATTCATCTATTTCAAATGGAATCAGAATCAAAATATA
GATCCAATGATTTATCAATTTCAAATTTAATGATTTAATTACTACTTCTAATTAAAAAAAATTA
AAATCGATAATTCTAATTTCCATTCGATCCGATGATTTACGATTAAAAAATATATATATTATAT
AATTTTTTTAATTTTAAATTATATATATATATATATATTAAATTTAAATTATTTTTAATTAAAAA
ACCAATGGTTCAAGCCAGAAGAACCGACCAAATTTGGTCCGGTTCTAATTCCTGATTTTGAGA
AATCATAATCATCGATTCCAAAACCGAAACCTTCATGTTTCGCAATTGAGGTCATGCAACTA
ATTCAATACTCAACTTCCAGCATGATCCAAGATAAACCTTGACTCTGTTCATCTCAATTACAC
GCTATATATGGAGAAGTCGAATTTATACATGAACGATTGACAACACGACCTAGTTTAGAATG
TCGATACAAGAATTGAAGAAGTTTACATGAATTGCACACATGCCTCGGTTTCGGTTTAGGTAT
CAGCACGATTTCGGAAGAACGTAATGCCGAAAAGAGGAAATGAGAACAGCTTCAGTTGTAT
Ma07_g116 CAGTTTAATCTCCCCTCGTGTGGAAAACTTCGACTGTAGAGAGGGAACTCGAAGAGGAAAGC
70 TACCTTGAACCATGCAGGCAATCTTCCGTACACGGCAGGTGGTAATGGCACCAGTGGGCTGC
GAGGCCGATCATCGAGTAGATGTGAGTGGGGGACACACTAGAAGGTCAGAATCTACAGCAA
AGAGAACTGACCTAGTTATGTTCTTGTAGATTCGGTATTCTGCCATGCTACCAAATCGTTTGTC
GGCCGATTCCTGATTCCACATGTGATGAATCAGAATATCACCATTAGCACAGTGAAATCCAA
TGTCAAGAGAACTAACATCTTGATTTATGTCTAGAACTGCCTACCTCAAGAAGTGGAATGCC
GCTGACAAAAAGAAGCAGGAGAGTCAGGAAGATGGGTCCAAACACCACAAGCCATTCTGC
ACCCTCCAAGACTGAAGTAGAGGCCACAAATATTCCCCACCAAAGGAAAATCTGTGCAAGC
ATACAATAAATTGAATCGACAAATGCAACATGATTGTTCATCAATAAAAGTGAGATTTGGGA
ATAAAACCAAAGTGAGACGTTTGTAAAACCAAAATTGTATGACAACATCGGATGCTTGTTGA
AGAAGCAACATTCAAACTCAAGATCTAACATCTTAAAAGTTTTGAACGATCTAGGACCCTAT
TACCATGCTTCTTACTGCATATTAACTCTAAATTAGTTCTGTAGAATGATCTTCTTGCCCAATA
CAGACAAGCAAGATGATTAAAGGGAACTGCTACTGAACGAGGTACCAAAAGAATGTATTTT
CTTCGGTTCGCTTGGGCAGCACAAGTTGCCAAAGCACATAAACAAAATTGGATGAAAAAGA
AAGCAGCCAGAAGATAAAATTTGACTGACCTCACCAAAGTAATTTGGATGACGCGAATATTT
CCAGAGCCCTGCATTACACCACTTTCCCTTGTTCGCAGGAGAGTTCTTGAATAGCAACTTCTG
CTGATCGGCAGTGGCTTCGATAGATATGCCTATGGACCACATTATCCA
TCGTTCTTTTTCCAAAGTATGGGTCTTCCTCTACCTTCTTCAAGGATGGAGCTCCTACCGATTG
ATAGAACGGAAAGAAGTTGTAACAGAGAACGCCGATCGCGTGCAAGAGAGACCAAAAAAA
GTCTTTTATGTTGTATGACAGAACACGATTACTTGAGATCATAAAGCCGAGGAGGTGGAAGT
AAACGTCACGGAAGACCCCCCACCGCTGTTAGAGAAAGAAAGGTCAAAAAAGGAAGGGAG
Ma03_g155 AAGACCTGGATACCAATCAGCGGGAGGAAAGAGACATGATTGTTATTATCGTTTCTCGACAG
80 TGATGATAATTTCAAGAGCGTATAACAGAGAGGTTGTCAGGGGAAGGTGCCGATGGCAGAA
GAAGCGGAGACCCGGTCACCGACCCAATCTGATCTCATCTTCCTTCTCAGGAATAGGATAAA
AGAGGTAATAAATAAATGAAAATTTGTGAGGATGACGGAGGTGGGCCGCCGATCAACCATA
GACCAACCTTTCTTAATTGAAAGTAAATCCGAATCTAGAAATTCGTTGGCGGAATTTCGATAT
AAAAATATGTCAGCTAATTTAATTAGAAAGTATTAATTTCATCTTCGCATGTAAATTTTTCTTT

TTTCATCTTTGCATCAATATCTTCATGATTTTTAATATGACATGTTAAGAATTTGTTGATTTATT
CTTATTTATTTTTATGATTATTTATAATTAGTTATACATATTTAACATTAACTAGATAAGCTTTG
TTTCTTAGAATAAATGGCTATACTAAAAATGATAACCAGATTTAAAAGGGATTTAGCAGGAC
TACCGGTATGTCACGCATATCATATTTATGATCTAATGGTGGCATCTATCATGATCAATGTGG
AGAAAATTTTTAATGATCTTGATCTTGATCCATCTTTTGAGCATATTCATACTTTGTAAAAGGA
TTGTGAAGACAAGAGAAAGAGAAAAAGAAACATAAATTGCATCTTGATAAAAAGGAATATA
TCATCACACACTTGCAAACAAACTTCTTGAGCAATAAGCATAGACTTTTTAAGGCTTTCCTTT
AGTCATTACCTTTAGTCATTCACTTCTCTCTCTAAGGTGCTACTACCTCTTTTCTATTCTGTGTTT
CTTAGGGCTACTGATCCCTTCTATTTGTAGTTTTTGTTGGAAAATCTTTGGGGGGATGACATCA
CATGGACAGTGAAAAAATAAAAATAAAAATAAAATCTCCTATTCCCAAATAGATGTTTGTCA
TCGTGCGAATATTGGTACGTAAAATTCATGAAATAGAAAAATGTGTATAGTAAAGATTGTGT
TACCTAAAGAGTTCGTATATCCCTGAATTCTTGCAGATCTCTAGGAAAGGGTGAAGGAGGTC
AAGCGCCCTCCTCTTTGGTGGTGATCCACACAACAGGCCTGCGACGATGCTTCACCAAACTC
CATGCCTGCTCTGAGGTGGAGAGGGGGAGGAGAATAAGATAGGCAAGCAAAAGCTCTATGA
ACCATTGAATCTCTCATATTTATAGTGGTCTCCTATCAATCTTAACGCTAATGGATCATTCCCT
ACTGGGTATTGGATCTTTATCAAATTATCCAAGCCTCTTATATTAGTGGATCTCCATCTAATAA
TCTCTCATTAGCTTTTATTGAACGAGCTCGTCTAAGGGATCCAATAATTCATGGGCTTATTGGA
TATCCAATAAGATAAGGGTTTTGGCGGATATCTCATATCTAAATCTCTACTCATCGCAATGCC
TACCATATGTGTGTGACCTTCTAGGCCCAATATCAAGTTGGCCGTGAGTTATACCTGTCAGAA
CTCCTTCTAACTTAGTGAATTATTATCTTCATAATAATTCACTCGACTCATCGACTAGGGACGT
ACTATGCCACTACGCTGTAGTCCCTAGACGATACAGGGGAATCTAATCTATTGGTCATATCTA
TCCTTAGTTATCATGTACCTATAGTCCCTCATCTATCTAATATCCTAGAGAACGT
TTTAATGTCTACCTTATCGACGGGAGCAGTGCAGGATTCGGCACTCCTTTTTATCTCTTTACCT
CCTCTCCGTTTCTGTCAGAGACGCCTGAGGAAGGACAGGGGAGGTGAGAAGAGAACATTTAT
AAGGGGAGGAAAGGGTACAACAGGGAATTCAACATAGTTTGCACGCTAGCGCGTCGCCCAC
ATTATTCTGCGCTCACAATAAAGACGGGGAAATGGCAGCTTCGCGGCATCGTCGTCCGTTGA
AATCGTTGGGGGCAACTGCTCATGCCCCTCGAAATACCAATTTCGCCCCCCTACCATAAAAA
CAAAAGGAGGAAAACAATATCAGCATTGCATTTTCCACCGTCCATTCCATCAGTACAACCCC
AAAACCAGCTTTATAATAAAAAAATTAGTCAAAAAATCATTTTTTTTCACAGAAAAATAAAT
TTATTATTTCAAAATCTTAAAAAACCCTCCCTTTATCTACTAATTACTAGAGTGGAACATGGTT
AATGAGGCTTCGTCTATGTCTTGCCTGAAACATAGTGACTCACATTATTGCGACGTGCAAGAT
Ma03_g077 CTAGAATAATACTAAGAAAAATAATAATATCCCAATGATTGGGGAACAGTACATGTGGTGTG
70 GCCGTGTTCTTCGCTGGCGGTCTTGCCTTGTATAATGGAAGAATTCGAAGCAAAATCTAGTGA
TGGCGATAGAGAGATTCCATGATTTTATGAGCTACCTTATCTATTAAATAGAAAATAATAAAT
TAATTAGCGGTAGAAAAATATCCAATGAAGTGTATGCTAACAACTTTAATATAATCATCGAT
GCTTGTCGAAGGAGAAGATGGCAATCTTTTATATCGAATGATCGTGAGGTGCTATGAAAGAT
TAGATATGATAGCGGATATTAAATTTATTTGATATGTTTAAATCAAGCTTAGTATCTGATTTGT
TAAAAAAATATAATAAAATCAAATCCGGGTTTACATTTTAGTATTTGAGTAGTTGACGGAAT
ATTAGTAGAGAGAAGTTGATAGGTATCTTTCCCTTGTTAAATAATAATGGATATATATTTTTAG
GGACATCACTAGTTGACTTGCTTGAGATTGGAATTGTACTTAGTAGTGAGATTTGATTTTGAGT
TTAGAGACATTTTTAATTAAATTCAAATCTTAATATGATCATAATTATGAAAAAAATATTCAG
TCTGATCAACATATCTTAAAACTTTTATTAACATCATTGCATATTGTAAGTGTCCAATCCTAAG

CCACTTTAGAGAATACTTTTTGTTTGGTTCGTAAATGTCATAGATTTAATTAAAATTATTTAAG
ATGTAAATAACAGTAGTCCGCAAAGGATTAGTCAAATTATTATATGTGTATATACAAGAAAG
AATTTGAAAGAGAAAATATACCATCATAATCTAGCAACCATCATTTAGTTGAGCGTCGAATC
ATTTGACAAGTAATTAGAAAACTAAAATATTAGTTATATCACATATAAAAGATTAAACTAAT
AAATAAAACATGATTTATATGAATATGAGGAAATAAATAAAAATAATTTAACTAAAATATTA
GTTATATCTCATCTTAATTTTTTTTATCAATAACATAATTTATACTTTTACATCTTAATAAAAAT
ATGATGTTGATGATGAATATGCCTAAATTTACAGTCAGTGTGAAATTATTCCTGAGATATAAA
TGTGTTATCCCTTTAAATTGTAGACGAATTCATCTCTTACGAACCAAAGCATACTACCGACCA
TACTTTTGGGCCACGTGCCACGATTAACTCGTTCCTCACAACATGTTATTATTGGCCAACCAA
ATGTAAGAAAACGATTATATACAGTAGCTTTACAAGCACGTGGCGGCGGCGGTGTTCCATTC
ACACGTGCTGAGATGGTGGAAGGTTCGTCGCCAAATGGTTCGGATCGGCGGCGGCCGGTTTA
ACGCGTACAAACGGTTCGACACCGTGTTATCCGTTTTCGACGGAGATCCGCGTTGA
TGCCATGACCGGTCTCTAACAATATCTAGTGGTAACGACAATTTATAATAGCAAAATTTATGA
AAGAGATGAAAAACAATATTAAGAATGGAACCTATATATATTGACAGTATCAGAAAATGAA
ATGTCCTATATTCATATCATGCCATTCAAGTTTCATGCATTGGATTTTGAAGTAGTGTACTTAT
CCACATCTAATGACTGCTAAGAAAACAAATCATATCAGGTAGATCATTTAATATTGTAAATA
TAATGATAAAAAGAACACAACGATCATTGTACCCAATTGGGACCAGGATGAATTATGACCG
ACCTTCTGAACTATTCCCAATTGGGACCAGGATGAATTGTGCACTTGGAAAGCATGAGTTGTA
TACACATCACTGTTGTGAAGCCTATCCTTGGCACATCATTTTTGTGTGATCGATCTACCAACTG
AACCCTCATTTAACACCTTTTTTAATAACATGAGAATAAGACAGTGATATTTGTCATTTTTTAT
AACAAATATGACCAATAATTGATCATAATAATAATAAAGACAATAAAGATAGCCTATGGGA
CCATAATAATAATATTATTATTATGGTCTATAATAATTTGTGTGCCATCTGAATCAATCAAGA
CAATAAAGATAGCCTAGGGGATAATAATAATAATAATAATTATTATTAGTGTAAACAACTTT
ATGTCGTGGCCTCGGGGTCGACGCGACTTGGTTGAGGTCCGAATGACGGGGATCTGGTACGG
CGTTCCTCGAGATTGCCCAGGCGGCCGATCACGGTGGTCCGGCCGGGACGTTACATCGAGAG
GAAAGCTTTTCCACAGCGCCGGGAAGAAGCAACCCCGTCCTTACACCTGCACACAAGTCGA
Ma06_g268 GTCGGAAGCTCGGCCCGACCCCTCCGACGATCAAGTTAGTGGATGTGGAGGGGGTTTCGAAG
40 TGCTCTCCCCCCTCGTTTAGAACTCGGGGGTATTTATAGGGCAGTTTAATGTTACTTGATGTGC
CTACCTGCGGGAGCAGGATCGTACCTCTGATGGCGTCTGACATCGCCATTGGCATTGCGTGG
AGAACTGAACTGCCGTAGGGCATGGGCGAGCCTCGGTCGTCGTTCTCCTCTGCCTCGGCCAA
GCATGTTGGGTCAGACAACGATGAAGTCGTTGTTTGAGAGCGGGTGACGTCAGCGCACATCG
CGTCAGCATTATTGCCCTCTTTGGGGCAGAGTGTCGTCCAAGCGTGGCTAATGTCGTGGCGCG
TCATGTCGTCATTATTACCCTCATTAATAATAATAATAATAATAATAATATAATCCATATGAT
AATTATACTAAGGAGGGAGATGGATAAAGATTAGAATTTATATTATATAATAATATCTTTATT
ATTGGTATTATTACTTACGTTCGAGTTTGGAATCGACAGTTCAACCTCCACACAAGATGGAAT
CAGAACCATCATTTTTTTTTTAATTTTATTTAATTTAATTTTTATTATAGTGTTTAATGTTTATTT
CTTTTTATTTTCATCTGGTTCTGATTTTGATTTCAACGTGAAATGGAACCCCCCATCCGATTCCG
ATTCGATTCAGATCTATGAAATCATTGATCGTTTTCGTTGTAATTTTCACCGGTTCGTTCAAAT
ATCACTATTTGTTTGTATCATTGTTATATTTATTTTGTGTGGAAAAGATATACAACTAAGTCTC
GCGCAGATGACACATAAATCAGAGTTCACATTGGACCGCGATTGGCGGCCATGTGAAACGC
ATGGATGCTCAATTATCAGGGAGTGGGTCCCAACAAAACACCTATTTAATTCAGTGGACCCA
ACCACGTAAAATTATTTGGGGAAGCATTTGTGGACACGAGAAACCCGTGCTTGTTCGACACA

Ma06_g26890	GGCGGATGCTCTGCATCAATAAATACCACCTCCGCTGCAATCGATCTCACTGCAACGTTCACT<br>CTCTAAGCTATTAGTCGTCGCTATCCACTGTGTTACAGGAAAAAGGGAACCGAAGAT<br>TTACCCTTCATTGGAGGGGTTTTCCACGTATATCTTGGTATTTATTTGATTGTGATTTCATTTAA<br>TTCCACTGTATATCATGGCCTGCTAGTATTTGTTCATATACAAAGGTTATTCCGCTTTTTATCCC<br>CATCAACTAGTATCAGAGTAAGGGTTTTGATGATTTAATTTTGTATTTGAACATGGAGGCCAG<br>TAATGTTTCTCACATGATTAGTTTAAATGGAAACAATTGGATGATATAGAAACCAAGAATGG<br>AAGATCTCTTGTATTGTAAAGATTTGTATGGACCTTTGCAGGGGGATAGTGCAAAACTCATAA<br>CTATGACAGATGATGAGTGGAAGAGGTTAGATTGAAAAATAATTAGATTTATTCGACAGTGG<br>CTTGATGATAGTGTCTTTCACTATGTTTCTACTGAAATTTCTGCATATTCTCTTTGGAAAAAATT<br>GAAAAGTCTCTATGAAAGAAAAATAGCTGGCAACAAAGCTTTTTTGATCAGAAAACTTGTGA<br>ACGTAAAATATAGAGAGGGTGCTTCTATTGCTGAACATTTGAATGAAATACAAAGTATTACT<br>AACCAGTTATCCTCTATAAAAATGTCTCTTGATGATGAGTTGCAGGCATTGTTACTTCTTAGTT<br>CATTACTAGAAAGTTGAGAGACACTAGTAGTTTCCCTCAGTAATTCTACGCCAGATGGTGTTG<br>TCACTATGAGTCAAGTAACAAGCAGTTTGTTGAATGAGGAGTTGAGAAGAAAGATTTCGGCA<br>ACATCTCAGAATAATTCACAGGCACTTATCTTAGAGAACAAAGGAAGGTCAAAGTCTAGAA<br>GCAGTTCACGCATAAGTAGAAGCAAGTCAAGATCAAGAAAAGATATTGTTTGCTATAATTGT<br>AGTGAGAAAGGATATTACAAGAACCAATGTAAGCAACCTAAGAAGAGCAAGAAAAAGGGA<br>AAAAAAGTGGAGTCTATAGAGTCAAAAGATAATATCAACTAGACCTAAGAGGTATACACCA<br>CGGGGCAAAATCAATTAAATGAAATAACAATCAAATAAATATCAAGATATATGTGGAAAAC<br>CCCTCCAATGAAGGGTAAAAACCACGGGGCAAACTAGAGATAATCCACTATGAGAATAATG<br>AATATACAAATCTCAATCTCTTACCCTAAACCCTAGCAACAATCACAAGAGAATAACTAGGA<br>TACAAGGATCACATCACTGCCTACAATAATTGAGAACGTAGCCACCACACCCCTCTTAATGT<br>TAATTAGGGTTAGGTTAAGAGGGGATGAGCTATGGGCTGCCCAAGCCTACTATGGGCTGAAT<br>TTGTGGACTGCCAATCTAAGAATATGAGCCTAAGAATTTAATAAATTTAAATATGCAAATAA<br>TTTTGTACCATTAAAGCATGTAGATTATTTTTATGTCTTAAAAGAGCATGGTTTCAATGCAACA<br>TATGTGCATTAAGACAATCCAGACAAATGTAATGCACAATGTATTAAGACAAAAAAATCATC<br>TCGATGATAAGTAATTTTTCTACTAATTTAGAAATGTAGTTAAAAGTCTAAGTTAACATAACA<br>CTTACTAAAATCGCTGCAATTAGTCATTTATAAAACTAAGTCTAAGTATTTGTTTGTATCATTG<br>TTATATTTATTTTGTGTGGAAAAGATATACAACTAAGTCTCGCGCAGATGACACATAAATCAG<br>AGTTCACATTGGACCGCGATTGGCGGCCATGTGAAACGCATGGATGCTCAATTATTTGCGTTA<br>TCCGCACATTGAAACAGTGATCAGGGAGTGGGTCCCGACAAAACACCTATTTAATTCAGTGG<br>ACCCAACCACGTAAAATTATTTGGGGAAGCATTTATGGACACGAGAAACCCGTGCTTGTTCG<br>ACACGCAGGCGGATGCTCTGCATCAATAAATACCACCTCCGCTGCAATCGATCTCACTGCAA<br>CGTTCACTCTCTTAGCTATTAGTCGTCGCTATCCACTGTGTTACAGGAAAAAGAGAACCGAAG<br>A
Ma01_g18060	TAGGGTCGCCGACGGTGGCGCTGATGAGCTGGAAGGAGATGCATCGGCCGAGGAGCTCGAG<br>CACGTTGTCGATGACGGTGCCGCCGAAATCGTTGAAGCCGAGGACGTTCTTCCGGATCAACA<br>CCACCGTTCCTCTCACCTTCACCCCATGCGACCCGCCGCCGACGATGTGACTCAGCATGTCTC<br>CAAGTAGCGTCATCGTACAACAGGAGCCAGAGAGATGAGGAATGGTGTGCTGAGGTGTGTG<br>GTGGAAGGGGCTACTTATAGATGTTCTTGGATCGAAGAGTGCAGATTACTTATGTACGTAATG<br>CAGTCTCTCCCTTATCTTAAACCGAGCATGCCAAGAGAACCCATATAAATCTAATTCTAAAAC<br>ACTATATGAGATTATACCCATGTGACATTTATTTGTCATATATCATCGACCGGAGAATACAGC

ATAAAGTTACATGTCTCGTAAGTCAAAACGCACCGAAGTTTCAGAACACGTGTCCGTTCACG
AGGCTAGATTCGATCGTCCGAATATTCAATCACTATCACAATTGGATCGTATTAAGGCGGTAA
AAAGATGAAAAGGGTGAATCCAACACATGAGATCGAATCACATGTAAGGAGGTATCGGCCA
TAAAGAAATAATCTTGGATCATTGTCGAGCTGTTTGTCCCATTTACAAACGCATCTATGACCA
AGAATATGAAGTCAAAAGTAACTCTTTGGATGCACGCCTCGCCAAACCCACTAAGGCAATTG
AGGAGGTTAAATTTTATGCAAACAAATAAAAAAATACATGGCTGGATTCACGTTTCAATCAC
GTGCTTTGTTTATTTTCTGATAGGCTGGCAAGGGGACCATGAAGAGACAGTGGCCATTGAGA
AGATAAGAGTCCACATCATAAGCTGCAATAAAAAGGTACATGTGGGAATAATGTAGCAAAG
TGGCCAATTCACGGTGGTGGTCCAAGTAATGACCAACCCTTGAAATATCATTCATGTAGGGTC
AAGATAGAAAAGGATTTAATAATTATATTTAACATAAAATGGGAAAAAATACTTTAAATTTG
TTTTAATATCAATGAATAATATTTTTTGTGATATAGAGAATTTGTCGGGTCACGTCTTATTCTA
TTATTTTTGGTTATATATGATAAGTTAAATTTTTTTCTTTCTTTCTCATCCTTATTTATATTTCTGA
TCCTCAATTTGTGGTCATGTCTACCTCATTGTCTATCGCTACTCTCAATAACGAGATAAATAAA
TGATAAATCGATGTAAAAATATTATTATTTCTTTCATAAATGAGTATATATGATAATACATAA
TGGAAGGATTTAATAATTATATTTAATATAAAATAAAAATGTATTTTAAATTCACTTTAATAT
CAATGAGTAATTATTTTTTGTGATGTAGAGACCCTAATAACACATAATAAAAAGCAGTAATA
TCCTTTTTTTTTCTCTATGACTCTACGTAAATAATCTATGAAAATATATTTACTTTATAAAATCC
TAGTGATGCGTCTCGTTCCTCCATCTTTAAGATACTTGTACGTAAAAATCGATTACGTGAAGA
AATAAAAAAAAATCCTTTAGATTTAAAAAAAAAAATGAAAATTCCTCGTTCGAGTATGAAAGA
AATCTTTTATGATTTAATATTTATTTAATTTGTATATATTGGGTCAAAGAGGAAGATTGAGATA
AAAGATGAAAAAAATTCTTTGATATTGATGTGAGAGGTGTATAAAGATATTTAGGTTAAAAA
TAAGTGAACCTTTAATTTGATCCCATTGTTGAGTTACGGTGTTTCACGTTTCAAGTCTTCTCTG
GCCAACTTATTATAAGACCTCCAAGAATCCATAAACTATGTTGCATGTGACCTGATATTATTA
TAGGAGCAGGCTTCTCGGACTCATAAAATTAATTATTATTATGAAAATATAAAATTTAATTTA
ATTTAATGTTCCGATACTATTGTCTTGATTCCCACGCCGTGGTATTGAGTCCAAGG

CACGAGTTTCTAAATATTGTTGGGTATATCTAATATGATCGTTCTAATGTTCAAGTCTGTAAAT
AGTCAAAGATGAGAATTAGGAAAAAAGATTGGTATTTGTAATCATTTATGAAACAAAGAATA
ATCTTTCCTCTCACATTATGTATTGGATTGATTAGGTTCTATATCTTGACTTATGATTGATTTGA
AATATTGATAGGTATTGCTTGTTATATGTTATGAATGAGGGTTAAGTTAGAGAGAGATGTGTT
TGTCCAAAATTTAATGAAAGTTGTTAATGCAAGAGATGAAGAGCAGAGAGATGGATAAGAA
CATCATCCCCTCTCAAGCCAGGATGGAGAAAGATGGAAGAAGATATCTCAACACCTCATCAT
CGAGCAATACATATAAAAGAATCATATCAGAGACAAGTGGTGCCAAAAGGGTCACTGTCTA
TACGTCACATTGTGTACTGCAACACAAGATTCTCATGATAAGGCATTTTATATCTAATTTAATT
Ma01_g164
00
GGATAGTAATTTTAATTATATTAAAACTCTTAAAAATAATCTTTTTATTTGAATATAGAGTTTA
ATTTAATATTACAATAAGAGATACTAAATCTTTAGTCTAGAAATACTTTTATCATGACCCGGT
ATATTCGTTGAGTTTGAATCGAGACCTTCGTACTAAATCGAGAAGATAATTAATCTATTAATT
TATTGATCAAAATATTTAAGTTGGGATTGATAATAATAATATTCTTATCAAATTTTTATAGATC
GATCTTAACTTTATAATAATCGAAATGTTACAATCTCATCTACCCCATTGACGGGAGATAATA
TTTTAGGAAACTACTGATTGAGATTTGAAAAAGATCTTCCCAAATATTAATTTTGTTCCTCTCA
TACTGGTCAATTATTGTTTAGTGTCATCCTCACCTAATATTGCCCTCATCAACTTTTCTTAAAG
AATATGTGAGCCATTGCAATAATTGCTAAGGGACCAACTTGTAGTACATTATTCCTTGTCTCC
TTATTTAATCTCCTCCTGCCTATCCCATATGACAATCTCCATTTATTTTTATGAATTTTCTATAA

AAAATAAACTACATTTAATGTTTTCTTAGTTTAGAAATAGAGTTTATGGTGTTGAGTGTTTGGA
GGAAAACTATATTACATGGAAAAAATATATATATAATTATGTATGACGAAAGATGTCTTTAA
TCTCTCTTTCAATAAAATCCCATCACTCTTCCTCTCTCTTTCAAAACTTATGGGGTCATCATCCT
ACATGAGCTGTCAGATTAAAAAAAGAGGCACAGGCAGTCTAGTGGTGCAACTTTTTCCACAT
CTTCTTTTATATTCCAAGCAAACTTGCACACTTGAGCTTTCATTTTTTTTTGTTTTGCAACCTTTC
TTATCAAGACATTGGAAGATCATTACATCCTTTTCCACTCAAATCCTATGAGATATTGTGTGTT
TGGTACTGAGAAAGAGATGATTACTTCCCTGTTTTTTGGTCCAAATGATAAAGCAAATTCATC
AGAATCATGAAATATAATTCAGAAATGTAAACCAAAATCTGAAAGCAAATCATGTCCATCA
GCTGAATCAAGAAAGACCAAGAAGCAAACAGTACTCGCAACAGCTCTCTCCCCATTCAATTC
AGGGACCATCTACTGGGCTTGGGAACGAGAACAGTAGAGGAGCGATGCAGGAAGCTACGA
AGCTCTGAAAGCTCATAGCAGGTTGAAGAAGACCCAGCAGGAGACGACGTCACGTGGTTCA
CATGGTAGAAGTGGGTCCACCATGGGGTCCATATGATGTGGCCATCTGATGCGTCGCCTCCA
ACATGGCATGTGACTTGGGACAGAAATATAGATCGTTCTACGCTTTGGCTCCCCATTTGCCTC
TATAAGTACCTCCCTATATCCTCCAACACATCCTCATCTCAGGCAGTGCATCTGCTACTTGTTA
CTTCCTCTCTTCCTCTCTCGCTTGGGTTGGTGTATCGGGAGGCAAGA

Ma06_g26850

TGGTAACGACAATTTATAATAGCAAAATTTATGAAAGAGATGAAAAACAATATTAAGAATG
GAACCTATATATATTGACAGTATCAGAAAATGAAATGTCCTATATTCATATCATGCCATTCAA
GTTTCATGCATTGGATTTTGAAGTAGTGTACTTATCCACATCTAATGATTGCTAAGAAAACAA
ATCATATCAGGTAGATCATTTAATATTGTAAATATAATGATAAAAAGAACACAACGATCATT
GTACCCAATTGGGACCAGGATGAATTATGACCGAGAGTTCATACCTTCTGAACTATTCCCAAT
TGGGACCAGGATGAATTGTGCACTTGGAAAGCATGAGTTGTATACACATCACTGTTGTGAAG
CCTATCCTTGGCACTTCATTTTTGTGTGATCGATCTACCAACTGAACCCTCATTTAACACCTTTT
TTAATAACATGAGAATAAGACAGTGATATTTGTCATTTTTTATAACAAATATGACCAATAATT
GATCATTATAATAATAAAGACAATAAAGATAGCCTATGGGACCATAATAATAATAATATTAT
TATGGTCCATAATAATTTGTGTGCCATCTGAATCAATCAAGACAATAAAGATAGCCTAGGGG
ATAATTATTATTATTATTATTATTATTAGTGTAAACAACTTTATGTCGTGGCCTCGGGGCCGAC
GCGACTTGGTTGGGGTCCGAATGACGGGGATCTGGTACGACGTTCCTCGAGATTGCCCAGGC
GACCGATCACGGTGGTCCGGCCGGGACGTTACATCGAGAGGAAAGCTTTTCCACAGCGCCGA
GAAGAAGCAACCCCGTCCTTACACCTGCACACAAGTCGAGTCGGAAGCTCGGCCCAACCCC
TCCGACGATCAAGTTAGTGGATGTGGAGGGGGTTTCGAAGTGCTCTCCCCCCTCGTTTAGAAC
TCGGGGGTATTTATAGGGCAGTTTAGTGTTACTTGATGTGCTTGCCTGCGGGAGCAGGATCGT
ACCTCTGATGGCGTCTGACATCGTCATTGGCATTGCGTGGAGAACTGAACTGCCGTAGGGCA
TGGGCGAGCCTCGGTCGTCGTTCTCCTCTGCCTCGGCCAAGCATGTTGGGTCAGACAACGATG
AAGTCGTTGTCTGAGAGCGGGTGACGTCAGTGCACATCGTGTCAGTATTATTGTCCTCTTTGG
GGTAGAGTGTCATCCAAGCATGACTGACGTCGTGGCGCGTCATGTCGTCATTATTACCCTCAT
CAATAATAATAATAATAATAATAATATAGTCCATATGATAATTATACTAAAGAGGGAGATGG
ATAAAGATTAGAATTTATATTATATAATAATATCTTTATTATTGGTATTATTACTTACGTTCGA
GTTTGGAATCGACAGTTCAACCTCCACACAAGATGGAATCAGAACCATAAATTTTTTTAATT
TAATTTTTATTATAGTGTTTAATGTTTATTTCTTTTTATTTTCATCTGGTTCTGATTTTGATTTCAA
CGTGAAATCGAACCCCCCATCCGATTCAGATTCGATTAAGATCTATGAAATCATTGATCGTTG
TCGTTCTAATTTTCACCGGTTCGTTCAAATATCACTATTTGTTTGTATCATTGTTATATTTATTTT
GTGTGGAAAAGATATACAACTAAGTCTCGCGCAGATGACACATAAATCAGAGTTCACATTGG

	ACCGCGATTGGCGGCCATGTGAAACGCATGGATGCTCAATTATTTGTGTTATCCGCACATTGA<br>AACAGTGATCAGGGAGTGGGTCCCGAGAAAACACCTATTTAATTCAGTGGACCCAACCACGT<br>AAAATTATTTGGGGAAGCATTTGTGGACACGAGAAACCCGTGCTTGTTCGACACGCAGGCGG<br>ATGCTCTGCGTCAATAAATACCACCTCCGCTGCAATCGATCTCACTGCAACGTTCACTCTCTT<br>AGCTATTAGTCGTCGCTATCCACTGTGTTACAGGAAAAAGAGAACCGAAGAT
Ma03_g11520	GCGGAGGAGGAACGCTCGAGAGTGCTGTGATGGACGAAGAGGGCTACGGGTCGGCTAGTCC<br>TTATAGAGGGCTCGCCAGCCGCAGCGGTGACTACAGTCTGCAGCAATCATAGATGAATATTG<br>TATTGTTCATCCATGGCTGCATGATTCACGTGGGAACTGACTGCTCTCTCTATCGTTCATTCTG<br>CTACAAACGATGACTCCTTAGGTAGCAGATTTGCGTCCATTTTTATTCCTCTGTCGATAGCATC<br>TGCGATATTTTACCGATTACGCCGCCGACGACGTGGACAGGGAGATGCAGCGTGTCATGAGG<br>CCCTTCTTCCCCAACTAGCGACTGGTTCCATCAAGATGAAGAGGCACAAGAAAACGAAGAG<br>ACCGAACCGTAGTGCAAGCTGGTCTGCATAGTTCTAACCCCAGCTAAGTTTCAATTGGAATC<br>GAATCTAAATTATTTGGTTATGGAATCTAAATCGACCCTACATGGTTTGGTTTCGATTCTCAAA<br>ATTTAAGAACCATGATCATTGGATCCGCAATCGGGGTTCGGTCTACGTCCTTATGCTTTATGCT<br>AAGCTGTCGATCAACAAGGATGGGGAAGAGGAAAGAAAATATCAATAAGGATACCTCTCCT<br>CTCCTCTACACCACACACGCTTAATCCATGCGAGCGTCCCCCTAAAGAACACATGGAGAAGA<br>AGCTTTCGGGTCCAACATCACCGAATCATGGAGACAATAACTATTAGTCTGTCGATTGCAATC<br>AATTCATCTAAACGAATGATCATTAAGTAGAGACCGATTGACGTGTATGCAATTATTGATTGC<br>AATCAATCTATGTCAAATTAAGTTATAAGAGTGACGAACGAGGTGAAGGAGTACAGTAGATT<br>TGGTGAGCTAAACAAGATTAAGAATCTCAAGAATAATAATAATAAATAATGATGACGAGTTT<br>CTCATGCCACAATCCTTAAGATTTGTGTCACATCAAACTCTAAAATAAGATTGAAGTACAAG<br>CATAATAGAGAGAGAATAAAAGAGAGAGAGATAATTGAGTCACGCATAAATAGAAAAAGA<br>TATTAGAAAATATTTATGTGTGAAAGACATCAAGATAATAATGTCGTATGGATAAAAGTGGA<br>AAGGGCCAAATTATCGATAGGGATTTATTCATAATTTAAAAGATTGATCTTTTGAGTTGGATC<br>GGATCACATTATCGATTTGTAATGGCGGAGTTCGATCTAACCATCACCTTAAAAAAAAAAAA<br>GGAACTTCACGTTGACCATAAATACCTAAAAAATAGGAGGAGGAAATGGTGATCACGGAAG<br>CCCTCGACGTTCTCCTCCACTTATATGCTACGAGCATGAGGAAAAAATGGCGTTTCCCTCCTC<br>CACATATCACCTTTACCCAGACAACACCGGCGTCGCAACCAAGTGGACCACCAAGGAATCA<br>GCTTGTAGTTCCTCGTCCTTCGTCCCCTCCTTTACTTCCATTAAAATGTCACCTCTTTCAATCGA<br>TGTTCGAGAACGATTCCAAGCGCATCTCAATCTCCATCCATCGAAACCGATGCAGATGGCTT<br>GGCGAGTAGCTTCGTTAGTCTACCGATCTCCATCAAAAGTTGTTTTACGTGTGGCCTAAAAGA<br>TGCCATCTGATGTGTCATTTCAAGGAATGAAAGAGATAAAGTTATGAAAAAACTCGATTGAC<br>GTCGGACAAACAATGACGGATTTTGGTTTTTAATGACAGGAGTAGAAATATAAGAAGAGATA<br>AATTAATAGAAAAATATAACTCTACGGAATGAATGAATGCTCTCATGAATGTCTCGCACCTTT<br>CACATGTCTTGTGACAGTGAGAAAAATCCAATCCGTTGATTATCTCTCGATTGATGCATCATA<br>ATATTTTATCATCTTCTATAGACATAGATAAAATTATGAAAAAAATATATGATTTTATCAAAT<br>AAAATGCATGATGTCATTTCTTGGAACAAAGCAATAATTCTAATTTGGTTTGAAAAATAATCT<br>TCCT
Ma08_g14590	AATAATATTTTCTATGAAAATCTGGGCAATACTAGCATTAGTGTAGGGATTTCGCATAGTACA<br>AAAATGAGCATATTTCGTAAGTCGATCAACCACCTTCTTAAATTAAGTTATTATTTTAAGTGG<br>TAGAAAATGAATGTTCTCTTATAAAATATTAAGTGTAAAAGTTCTCTCCTAATTCTAGAAAAA<br>AAATTATGATTGTTGATCTTCACCTATTGGAAGAAAAATGTTAGTAAAAGCCAATGGCTAGG

ATAAAGGAATCGAGAGTAGTCGACGATCATAAAGACCAAACCACTATAATTTTATTTTTTGT
AGGTGTCTTTCTCGATATTTGTTAACTTTGTTTCTGAATAACTTAAATAAGAACTTATTTTAAGT
TGAACTTAAGTAAAACTTGAGCTAGCTCAATAATCCTTGAGTTCAAATTCGAGCTTCAGCATG
GTTCGATTGAATTAGGATCGACTTGGAATTAAACTTGAATTATGATTGAGCTAAATTAAAGTT
CGATTCAAACTTAGCCAGATCTTCCTATATCAAGAAACTTATTCCAAGTAGGGATATATATTT
CACAAAGAAAGAATATATTAAACGTTCTTGATTATAAGGGTCTCCTTGGTCCTTACATAGAGT
AAAATATGAAGCTTACTCCATAAGATATTGAACTCCTCAAAGATCCATCGTAAGATATAAGA
CTTATTGGGAGATTTATTTATCATACAATCACAAGGCCCGATATAATCTACTTCGGGCATCTTT
TTAGGAAACAACATCTTAGAATGCTATAACAAAAACTATTTTTTCAGCAAAGAATAATCTAA
ATATAACAAGGTATTATGATGCTAATTGAAGAATTTGTTCAACAATAAGAAGATCAATATAC
AAGTATTGCATTTTTTTTCTTAAGAATTCCCTATCTCTTGGTAGATAAAAATAATAATTAACTA
TTTCTCATTCTTCAGCCAAAGCATAACATTGCCAAGTTTTTTTTTTTGTTTTTTACACCGCACT
TACAAACCCTTAAATAAAAGAGCTTGTTAGACATAAACTAACAAGTGGCAACTAAAAGAAA
ATGCTATGGGTTGCTCACCCCATTATGAAGCTAAAACTTTAGATTTATCAAGCTAATGAATGA
ATCTTGGATCTTTAACTTGCCAATATGGTCTTAGAAGTTACTGTCTCTGTTCTCTGTCAAAAAT
AAATATCCAAACCAGAGAACATGACTTTTGAATCATCAGCAAAGTACAGAAGAACCTGCAA
TTTCGCAACGATGCTATGCGCTGTAATAAGTTGACATCATGCTTGCTTATGCTCTGCAAGGTG
GACCAGCGGCATTTTTTTTAATTTTTCTCTTGAATCTGGGAATCACGTTAGGAGTAAGCAATA
ATAGCCAGATTGTGCCACGTGCCCAAATGATGGTTCATAAACGTATAAACCAACAAGCTATT
GTTATTAATTTAGAAACTGTTGTCGGTGGTAGCATGATTTACCAAATACATCAAGCTGAAGCT
TTTTCGCTGTGATGTCTGAGCTCACGTTCAGTGATATTAGAACTGCCGTGACTTATAAAAGCA
CAACCTAATAATAAAGAAACCAGGATAGGTGCGGTCTTCATCTGAGGCCACAGAGTCGTAA
GATATTTTGGTGTACTACTGTTTCCTTTATCACAAGAAAAAATCCACTTAAGTTGGTTGGATGT
GTAGATATTCTTCCATGATGACCCCACGTCATCTGATCCTGAGTCATCCAGCCGTCTGATGAG
ATAAAGATTGGGAGATATTTGATCGCTACCCATGAGCTACAGCACAAAAGTTAGTCACGGGA
CAAATAGGACATCGGAACGGCATGCCTCCTCAGTTCTCCCTTCCCCACATCGCCGTTCCCCCT
CCCTTCTCCTCGCTATAAATCCAGTGATTGAGCAAAACCAAACTCATCAAAGCTGCTTCTTCC
TTTCTGTCTGCATCGACATATAGTGAGAGTGCGAGAAATAGAGAGATCAGAAT
ATGTCTCCAATTAGCTTCACCGTATAACAGGAGAGAGAGACAGAAGTAATGGTGTGCAGAG
GTGTGTGGTCGATTGGCTATTTATAGATGTCCTTGGATCGAAGAGTGCAGATTATTTATTGACT
TTGCAAAGAGAAGTCATTTAAATCTAATTCTAAAAGACCGGAATGACGGAGTCACGTCAATA
AATAATCTCACACACGTGATATTTTTTAAGTTATCCGTCTCGTAAGGCCGCAAAAGAGGACG
ATGCATCAAATGATTGAAATTAGGCCAATTTAATTAGGGGCCCATTAGATCTAAGTTAGGAC
CGAGTTGGGCCATCACTTGGAGTACTACCAGGGTGGGCGATAGAATAATTCAGAACTCCGAC
Ma01_g180 CATTTACCAAAAGTTCATATCATCGTAAACTAAACTTAACATTCCGAAATTTCAAACGAGAG
20 AGGTCTTTTAATACCTTTACACAATATATCAATTTTAAATCTGTCGATAACATTTCATTACATA
CAAAATATGCTTATACAACAACTACATAATAACCAATAAAACTTACCCAAAATCTGAATAGC
TCTCCACTACCTTAGCCCAATTCAAACATCCATATGAGTGATAAGCTAACCTGAAAGATTTAT
ATAACAACGGAGTGAGCTAAAAAGCTCAACAAGTGATAAAACATATCCAGAACGAAAAGG
AACAGTTTCAAACAAATAAGGTATCAAAATGCAAGGTAGGATATAATAATTTATAGATATAA
TCTTATGAGATACAGAATCGCAAATGTCATTTCAATTAAAACATATTTGGTTCATAATAAATG
GAATAAAGAGTAATTGGAACATATCAATGGCGTATAAAATGTTCCGGAGCATATCAACGAC

ATATGGAACATTTCGGAGCATATCAATGGCAAATAAAGCATTTTGGAGCATATCAATAGCGT
ATGAAATGTTTTAGAATATATCAATAACAATGAAACATTTCGGAATATATTAATGGTATATGA
AATATTTCAAAGCGTATCAATGGTGTATGAACATTTCAGAGCATATCAAAGAATAAATACAA
AACATAAGCTCAAACATCGTATTTGACGTTGCATTCATATCATTCTTACCCAAAGCACATATA
GAAGCACATAATCAAAGCTCTAGATATCATATCAAATACATAGAAGGTGTAGTGGGATCAC
AAACGGAATATAGTTCATCCATTTCTGGATGACCACTAGACAAAAATCTCCCATATTTGGGA
GCTCCATCCACCCACATCTGGGTGAAGTTAGAGGGGGGCCAACAGAGCATCGCAGACTCTA
AGCATAATCCCTTCATTTTTGGCAAAGGTCCAAATACTCTCACAAACACTAGAGTACAAAAG
GGCATTATGAATAATAAATTGACACATATAGGAGGCAAATGCAAAATAATGAAACATACAT
GTGCCTTTATAAGTCAATACAATGCAAACATAATCTTTCACAAATGCATTCAAATTTGAAAAG
AAATAAAGCAAGAGCATACAAGAATTTTGGCACATATCAGAAGCACATGCGAAACAACAA
AACATATATATATGCCTATACGAAACAATATGATACAACCCAAACTTTACATAATAAGTTTC
AAGAATATAAGTAATTTAAGAAAAAGATCATTAAAGAATACATGCTAGATGAAATAAATTTC
TAAAGGGAATGAAATAAGAACGGGCTAAATCGATCAAAGCTTGATTTTTGACAGAATTCGA
GAGACATATTGAACAAATAATTTAAACTATCAATTATGCTCCAATTCACTCAAGAAAGATAT
CATTGGAAAGATATTTTAATCTACTTTTAGATAAAATAAGTTTCATATGAATTAAAATTTCTA
ACATAGAGTTATAGATAATTTGATAACCAAAGAACAAGGAACAAAATCTCAGTTTTATAAAA
TTCATAGGGTCTGTTTCAATATATAACTAGGCATGCATTTAGAATCTAAATCTTTCAATCCATA
TATCAAAAGAAAGAG

Ma06_g26870

CCAAATATTCCTCTTTATATTGCTTTGAAAATAAGTACAACTTCTATTGCACCACATGGCACTT
GCTACATGTTTGCATGTGACATGGGCATATCTCGATGTCTTTCCGATAAGGATGCACTTCCAT
GTGACATGCGCATATATCGATGTCATTAGATAAGGACTAAAATGCACAAACCAAATTATAAA
ATAAAAAAGGACCCACTTGGGGATTCAATCCAATAATTCACTTAGTTTACATGCATTACAAG
AATACACCATTTTAATTGCATTTGCATCTCACTTTGCATGACAAGATATCAAGCTGTGTTTGAC
ATCTGAATAGCTTTATTTTTTTTCTCTCCTATGTCGGAATGTCCCATGCAATTAGTTTATTGTTG
GTTTGATTGGGGAATCATTCAATATTGTAGAAGCTTGCACTGCGAGTTGTTCTTAGACAAACG
ATTATGATAAAAGATTGTCCTACCTATCTGCATGTTTGGTATGGGAATAGACTTCACATGGTT
CACAAGACATTGTACTAACCCATCTTTCTGGAATAGTATTCTCTGGAAAATTGTGTTCATAAG
AAAACAGTATTGGAATTTTTTCTGTACAATTACAGAGATTATGATTACCCAAAAACAAGACA
TCCATTTTCTAAAACAATATCTTTCTTGTTTATAAATTGTGAAAATAATTTTTCTGAGCTTTTGA
AGCATCTTACCAGATTGAATTTCTCAATAGAGACTCTCAATGTATTTCTTATGAATGGCTAAG
TATAAGTTAATTTCATCATGTATATTGATTACTCGATTGATCAACTATGGCTATAGCTCTATAT
TGTTGTGTTTGGCAAGATGGCTGCATAATTATCGACATAGCTTAAAACCATAGCAACTATACA
TTCTTTGATCTCTTGAAGACCTTAAGACTTTGTTGATACTATTCTTGTAAGGTAATTTCTTTTTT
ACTGAAATAATAGGAAAATTAAATTTTTTTATTTTTTAGGATTTTTTATATTTTTATAGTGAATT
AAATTATCTGATCAATGATGACCAGATGTGAGCCTAAGAATTTAAGTAATTTAAATATGCAA
ACAATTTTGTACCATTAAAGCATGTAGATTATTTTTGTCTTAAAAGAGCATGGTTTCAATGCA
ACATATGTGCGTTAAGACAGTCCAGACAAAAAATGTAATGCACAATGTATTAAGACAAAAA
AATCATCCCGATGATAAGTAATTTTTCTACCAATTTAGAAATGTGGTTAAAAGTCTAAGTTAA
CATAACACTTACTAAAATCGTTGCAATTAGTCATTTATAATAACAGTTTTATAAAGCGACAAT
CCCTTATCTTGTCTAAGATGAGTTTTGTTATAGTGACTACTCGAGCATATGCCAATAACATCGC
ATGTGTTCTCCTCAACTCGATCACTCTAGGTTTCTTTTAAGTGATATGATTACTATAATAGATA

GATAAAAATATTAACTTAAACTTTGTCAACTATCACAGCCAAACAAGCAGAAATAAAAAAA
GAAAATGTTCCTCATCACATTAGCTTAATGTGCCAAATAATTGATACATAGTCAGATGATAAC
AAAGGTGATAGCTGATAAAAAACAAATAACAAAATAACAAAAGTGCATCCTCAGATCTGAT
CTATCTTCAAACTTCATTTACTTGTCCTCATTGAATTCTAAAAGTGCGTAGCAATCCGTGCTTT
CACATTAATTAAATCATACACTCCTATAAATCCGTTCAGGTCTTGGCTAAATCCACATTTCATT
AAATCAACGCAGCTTCTCCTCGTTCTTATCCTAATCTTTTATACACAGACGCTTCTTATATTAT
CCAAGTCTCTCTCATGGTGGGTAAGTTGAAACCCGTGCTTGTTCGACGCAGGCGGCTGCTCTG
CATCAATAAATACCACCTCCGCTGCAATCGATCTCACTGCAACGTTCACTCTCCAAGCTATTG
GTAGTCGCTATCCACTGTGTTCCAGGAAAAAGAGAACCGAAG
ATGTCTCCAATTAGCTTCACCGTATAACAGGAGAGAGAGACAGAAGGAATGGTGTGCTGAG
GTGTGTGGTGGATGGGCTATTTATAGATGTTCTTGGATCTAAGAGTTCAGATTATTTATTGACG
TGATGCAGTCTCTCCCTTTTCTTAAATCGATAATGCAAAGAGAAGTCATCTAAATCTAATTTTA
AAAGACCGAATGAGATGATACCCATGTGACATTTATTTGTCATATTACATCTACTGGAGAGA
CTGCGTCACGTCAATAAATAATCTGCACTCTTCGATCCAAGAACATCTATAAATTAAATCGAT
AATGCAAAGAGAACCCATCTAAATCTAATTCTAAAAGACTGGATGAGATGATACCCATATGA
CATTTATTTGTCTTATATCATCGACCGGTGGATTCAACATAAAGAAAGTTATCTGTCTCATAA
GTCAAAACGCATCGAAGTTTCGGAGCATGTGTCTGTTCACAAGGCTAGATTCGATTTTCCAAA
TATGCAATCACTATCACCAGTGGGTCATATTAAGGTCGTAAAAAGATAAAATGGGTGAATCC
AACACATGAGATGGAATCACACGTTAGGTATCGGCCATAGAGAAATAATCTTAACTCATTCT
CGAGCTATTTATCTCATTTACAAATGCATCTACAGAAAAATACGGTCATCATATATATATATA
TATATATATATATATATATATATATATATATATATATATATATATCATCCAAATCAAGAATATG
AAGTCAAAGTAACTCGTGGGATGCACGCCTTGCCAAACAAATACAAATGCATGAATGGAGT
CACGTTTCAACCACGTGCTTTGTTTATTTCTGATTGGCTGGCATGTGGACCATGTAGAGACAC
TGGCTAATGAGAAGATAAATAGTCCACATTATCAGCTGCAATAAGTAGGTGCACCTGGGAGT
Ma01_g180 AATGCAGCACATAGGCAAATTATGGTGATGGTCCAAGAATGATGAACCCTTGATATATAAAA
40 GAAATGTCATTCATATACGATGAAGTCCAAAAGAATCAGATGATACATATATGTATATTACG
TTGATACAATAATTTAAATTTTTTGAGTTGTATTGATATTCAATCTCATGTATATTATATCTAAT
AGTATGACTGAAACTCACATGAAGATGATGTCCTCAAAATTATATGATCTCAATATTTTATAT
CTCAAAATGAGTTTTGATTGAGGTTCGTAAAATAAGTATATTAGATTAGTTCTTATCTATTATT
TTTTGTTATATGTGGTAAGATGAATTCTTTTTTTCTTTCTTCATCCTCTGGCTCTACTCTCAATGA
GATAAATAAATGATAAATCGATGCAAAAATATTATTTCTTTCCTAAATGGGTATATATATATA
TATATATGACGATGCATATGGAAGGATTTAATAATTATATTTAATATGAAATAAAAAAAAAA
GTTACTTTAAATTCGTTTTAATATCAATGAGTAATATTTTTTGGTGATGTAGAGAATTTGTCGG
GTCCCATTGACTAGCCATTCGAACGACACATTTCATTAGGGATACATGGATAGCATCCAACA
CATACCTCAATGACCGTTCATATTATTGTTTCGGTTTGTTCAACATATTTATACGAAGGCCACA
AAACGTATTGTATCTACGGGTTATCGTTTAATTATCATAATGCATAAATGATCTAACGGATCA
TATATTCAAGATTGATTACTCGACTTTATCATGATTTAGATTGACCACTTTCGTTAATGGCCTT
TTTAATTTACGCATATGATTTTTCTAATCACCAGATAGCAAAGTGTCATCCTGTATTATATTAT
TAGATTAAATAACACATAATAAAAAGTGGTAATATCCATTTTTTTTTCCTCTATGACTACGTA
AATAATCTATCAAAATATATTACTTTATAAAACCCTAGTGATGCCATCTCGTTCCTCCATCCTT
AAGATACTTGTATGCAAAAAGATCGATTATGTGAAGAAACAAAAGA

	TCCCGGAGGCCAAAGTCCACAACCACAAGTGAAGAAAGGAGGGAACTTTACAAGATCTAGT<br>GAATGTATATATCAGTCTCAGATAAGAAAAATCAAAAAGGAGGAAGCAGAAGACGATGAA<br>GATGAAGATGAAGATGATGATGAGGAGGACCGAGGAACCGTCGAGCGGAACAGAGAGAGA<br>GATGGCGATCGCAGTGGGTGTTTGATGGAGGGGAATTATATATTTGTAGCAGGACAGTATGTT<br>GATGGAGAGTACACGCGAAGGTTCGCATGGAGATTAATGCATGTGAAGGTTTGTATATTTGC<br>AAGGTTTTGAATTTTCAAAAACTTTTATGTTAAACTGAATTATAAAGAATCACATACAAAAAA<br>TCATTTATTTTGGGGATGTATATATGAATTTTTGCATGCTTTTGCATGTAAGATTTTGAGTTAGG<br>CTAATTAGATTTTTATATATTTATAAAAATATTTTATTTTTTTTCTCATTGATTTTCTTAATATAA<br>ATACATATATTCTAATATAATTTTTAAAAATATAAATATCGTAACATTAAAATAATTAATTAC<br>AGAAATTAATACATAATTAGCCCTGTTGAGTCGGCATCAGCTTCGAGCAGCTCCAATGTGTCG<br>GTCGAGTGACCAAACACGTAGTAGGGAACTCTCATTGGCCTCACAGCAGCGCCGTTGGGTCT<br>TTCCATGGGACCCGCGACTGCTGACCAATGATAAGTGCCACGTGGCATATATGGTGGCTTGC<br>CTGGCCCATAGGATCCGTTTCCCACGTGACGTTTCAAATCGAAAAAGGTCCTTAGAAAGCTT<br>ATTTGTCGATGATATATATATATATATATAACGCACAGAAATTATTATTTATAGGTGGAAAAA<br>GGCACGTGCGTAAGACTATTTGCACGTGTAGACACATCTAATTAAGGGAAAAAAAGGCTATG
Ma06_g30170	ATTAGGTGTATTTCTTAGAATTTTACTTAGGAAGGACTTAAACAACGAGATACTTGTCATATT<br>AAAAAGGAATAAAGGTAGGACTCAGATATAACAATAATAGGTAGGAAATCCAAATGGTATT<br>TTCGATATCTTCATAAAAATGTTTCTTTTAGCCACTTGGTTTCCTGGGAATATCTATATTTGCCC<br>TTTTCATCAGTACTTACCCCATGCAAATGCTTTGGCTGACCCACTTGATTGCAGAACAAACTT<br>GACCGCCTGGTGCAAGTCATCTCCGAGCTTTCCCAGTCCTTTCAAGATGACTAGTGCCACTCA<br>TTTTTCTGTTCTTGAGATTGTTCATTGTGGTCATCATCTCACCTTTTCCTCTCAGAAATGATGCA<br>GGAGAAATAAATACTGGAATTCACTGATTGAACAACGACCTAAGTTCATACTTGGATATGCT<br>CCTTTTATTTTAGCATGAAATAATGATATCTAATGCAAACTGTGTGGGGAAATGATTGTGCGT<br>ATTATTCACAGAGAAGGCTGTGAGATTGCATTGCTCCGATCATCTACAGTAAGTATTCCTCAA<br>GTACTCAGACCCCCTTCTACGTGAACAGCACCTGCAGTTGGGACAGATAACTTTAAGCTCTCA<br>GGCCTATTGCAGCACTACCTTTCTGCAGCCAAAGCCATGGAGGCTTAAAGGAGTCTGATGCT<br>AATTGCTCAGCCGAGGATGGATGGATGGATAGAAGCATGATGTGGTGAGAAAGCAAATGAC<br>GCAAAACTATGTTACATCAATTGGAAGATGTCTTACTTTATGGTGAGATCATTTCAGAAAAAT<br>GATGGCCAGCAGAACAATGTCAGATGTGGTCTGAGTTATCTTTACATGTCTAATGATTGCAAG<br>CAATGGACCCACAATCAGGTATAGAACTCATAATTCAGCATGCCATTTGTCAATGCTGATAA<br>TGTTAATAACAGTAATATTATACGAAACTTTTGCAAGTGACGCATGATGTGTAGTACAAGAA<br>CAGCAACATAACCTGTTATAGTCCTTCCTGATCATTAAATAATGGAAATAAGAGTTGTCG<br>TATTCTAAAGATAAGAGAGGGCGGTGAAGGTTTCGCAACTGCGTCGAAGACGATTGGCTTAC<br>GGTGTATATAAAGACGAACTTGCGTGGTGGAACAGTGGGCTTAGCTTAACCCATACGACATA<br>TGTGCGTGTCCGAAGTCAAACAGGGATTTCGTACGAAAGTAAGGTTGGGGAACACATTTAAA<br>ATGCAAATATATTTGAAACATGTGTCGAGGGTTTGTCCGGTGAGAATACATTTCAAAGGTGTA
Ma09_g12090	TCGACGTCTAATATATATTTTAAATATGAATTGATATTTAGTATTTTTTTAAAAATCAAACTTG<br>CTTCGATGTTATATTTATTATCTAAAATTATAAGTTTGTCAAATTTAGTAAGAAACAAATAAA<br>ATTCATGTCTTTGTAGAATTTTATTTATTTATTTTTAAAATTATTTATATTCATTTATATTATTTT<br>AATTTATATTTATTATATATCTTGTCCATACAAATTTTTTTTATAAGATTATATTTATTTATTAG<br>AAGGATAAATTTATCATATTATATTTAATATATTTTTAAAATATAATTTTATCAAATAATACTT<br>ACATATATATTTCTATTATTTATCGAATGCTCAAAACATTTCATACTTACATATTCACTCAAAC

TTATTTTCAGCCTAACATCATCATCTTATCCCATCATTTCTAATGTGGATCTTCGGAAAAAAAG
AAAAAAAAAAAGTCAGGTTTGTTCAACTATGTAATGTGGCTCAACAATAATATTATAGTAAA
AAAACCTAATCATGCAGAGGAATTTAATGACAGAAAATTCAAATAGAGATTGGATAGTTGGT
ATGGTTGCCCCAAAAACCACGTAAACGGTTGACAATTTGGCTTCAAGCAGACCGACACCGAT
AGATTTCGGTTCCCATTGCTATTCCTAAACGTGAAGTCCAAATCCTCCATCAACACAGGAAA
ACGAAAATGGCGGTAGGAGTCCAAATCCTCCATCAACATATGTCACGTGTTCTCAAACTTATT
GAGACTGCTGAGTTGTCGTGCCACGTCTTATCGAAGCTCCAGTGATTTCAACGATCAATCATC
ATAACGTGTAAGACTACTGAGTGTTATTTTTGGGTTGGGACTCCGTACGGTTCACTAACAAAG
CAAATTCCACGTGTAAAAGCCTTCAGCCTTCAGCCTTGGTGTTTGACTCGTCGTAGATGTGAT
GATCCATTTCGAGCGCGTTACTATTTCCTTAAACGGATATATATATATATATATATATATATAT
ATATATATATATATATATATATATATATATCAATTCCATGCGAGGTTACGTAAAGATATAT
CGAAAACTCAAAGTCTCCACCTCAAGCATGCTACTGTTTCTCCAGACTTGTTGGCACAAACTA
CGTGGCATGATGGAAACCATACTGACTTCCACCAAACGTGAGGATATCTTTTACGTTACATCC
TTAATTTATGATGATTGTGAACCAATCACCGCAAAAGCAATTTCAAGTGTTCATCTTTTTTTTT
AATACAATAACTACTTCCTTATACACTTATGCACGAGATATTCTTTCGATGTTGCCTTCTCCGA
TCTAAGATATGTGATAGCTTGACTTAAAACAAACTATAGTAACACATGCAGATAAATAAATC
TTAAAAAATTTTGACATGCCATAAAATAACACTCAATACATGAATATTAACCCCAGTTTCAA
ACGAAATATGAGTTAAGAAGCACTACAAAAATCGAGTCCATAATGGATATCGTTTTTATATTT
CGACTTTCTTTGTTATGAATAAACTTCTATCAGCCTAGTAGATGAGCTAAAAAATAAGTTAAT
TATCTTTCTCCGATTATAATCTTATTAAATTATTACGATTATATTAAGTCATGCTCATATCATCA
AATAATAGAGAATGGATCGATCTTTTTCTATTGATATTATTATTTACTTGTATTATAAAAAAAT
CATTAAAACACCAACAAATACAAATTAATTTTTTCTAATTTT

Table S6. *Cis*-elements in the promoter regions of *LOX* genes

Genes name	*Cis*-elements	Function
Ma01_g16400	ARE	cis-acting regulatory element essential for the anaerobic induction
Ma01_g16400	HD-Zip 1	element involved in differentiation of the palisade mesophyll cells
Ma01_g16400	HD-Zip 1	element involved in differentiation of the palisade mesophyll cells
Ma01_g16400	TATC-box	cis-acting element involved in gibberellin-responsiveness
Ma01_g16400	TC-rich repeats	cis-acting element involved in defense and stress responsiveness
Ma01_g16400	TGACG-motif	cis-acting regulatory element involved in the MeJA-responsiveness
Ma01_g16400	TGACG-motif	cis-acting regulatory element involved in the MeJA-responsiveness
Ma01_g16400	GT1-motif	light responsive element

Ma01_g16400	CGTCA-motif	cis-acting regulatory element involved in the MeJA-responsiveness
Ma01_g16400	CGTCA-motif	cis-acting regulatory element involved in the MeJA-responsiveness
Ma01_g16400	AuxRR-core	cis-acting regulatory element involved in auxin responsiveness
Ma01_g16400	O2-site	cis-acting regulatory element involved in zein metabolism regulation
Ma01_g18040	O2-site	cis-acting regulatory element involved in zein metabolism regulation
Ma01_g18060	TGACG-motif	cis-acting regulatory element involved in the MeJA-responsiveness
Ma01_g18060	TCCC-motif	part of a light responsive element
Ma01_g18060	LTR	cis-acting element involved in low-temperature responsiveness
Ma01_g18060	ATCT-motif	part of a conserved DNA module involved in light responsiveness
Ma01_g18060	GT1-motif	light responsive element
Ma01_g18060	GT1-motif	light responsive element
Ma01_g18060	GATA-motif	part of a light responsive element
Ma01_g18060	G-box	cis-acting regulatory element involved in light responsiveness
Ma01_g18060	G-box	cis-acting regulatory element involved in light responsiveness
Ma01_g18060	G-box	cis-acting regulatory element involved in light responsiveness
Ma01_g18060	G-box	cis-acting regulatory element involved in light responsiveness
Ma01_g18060	G-box	cis-acting regulatory element involved in light responsiveness
Ma01_g18060	CGTCA-motif	cis-acting regulatory element involved in the MeJA-responsiveness
Ma01_g18060	G-Box	cis-acting regulatory element involved in light responsiveness
Ma01_g18060	G-Box	cis-acting regulatory element involved in light responsiveness
Ma01_g18060	G-Box	cis-acting regulatory element involved in light responsiveness
Ma01_g18060	G-Box	cis-acting regulatory element involved in light responsiveness
Ma01_g18060	G-Box	cis-acting regulatory element involved in light responsiveness
Ma02_g07800	TCA-element	cis-acting element involved in salicylic acid responsiveness
Ma02_g07800	O2-site	cis-acting regulatory element involved in zein metabolism regulation
Ma02_g07800	O2-site	cis-acting regulatory element involved in zein metabolism regulation
Ma02_g07800	LTR	cis-acting element involved in low-temperature responsiveness
Ma02_g07800	LTR	cis-acting element involved in low-temperature responsiveness
Ma02_g07800	MRE	MYB binding site involved in light responsiveness
Ma02_g07800	GATA-motif	part of a light responsive element
Ma02_g07800	Box 4	part of a conserved DNA module involved in light responsiveness
Ma02_g07800	GCN4_motif	cis-regulatory element involved in endosperm expression
Ma03_g07770	ATCT-motif	part of a conserved DNA module involved in light responsiveness
Ma03_g07770	TATC-box	cis-acting element involved in gibberellin-responsiveness
Ma03_g07770	GT1-motif	light responsive element
Ma03_g07770	MBS	MYB binding site involved in drought-inducibility
Ma03_g07770	TGACG-motif	cis-acting regulatory element involved in the MeJA-responsiveness
Ma03_g07770	HD-Zip 1	element involved in differentiation of the palisade mesophyll cells
Ma03_g07770	TCT-motif	part of a light responsive element
Ma03_g07770	ARE	cis-acting regulatory element essential for the anaerobic induction
Ma03_g07770	Box 4	part of a conserved DNA module involved in light responsiveness
Ma03_g11520	GATA-motif	part of a light responsive element

Ma03_g11520	GATA-motif	part of a light responsive element
Ma03_g11520	GCN4_motif	cis-regulatory element involved in endosperm expression
Ma03_g11520	G-box	cis-acting regulatory element involved in light responsiveness
Ma03_g11520	G-box	cis-acting regulatory element involved in light responsiveness
Ma03_g11520	TGACG-motif	cis-acting regulatory element involved in the MeJA-responsiveness
Ma03_g11520	TGACG-motif	cis-acting regulatory element involved in the MeJA-responsiveness
Ma03_g11520	TGACG-motif	cis-acting regulatory element involved in the MeJA-responsiveness
Ma03_g11520	TGACG-motif	cis-acting regulatory element involved in the MeJA-responsiveness
Ma03_g11520	TGACG-motif	cis-acting regulatory element involved in the MeJA-responsiveness
Ma03_g11520	GA-motif	part of a light responsive element
Ma03_g11520	G-Box	cis-acting regulatory element involved in light responsiveness
Ma03_g11520	G-Box	cis-acting regulatory element involved in light responsiveness
Ma03_g11520	HD-Zip 1	element involved in differentiation of the palisade mesophyll cells
Ma03_g11520	ATCT-motif	part of a conserved DNA module involved in light responsiveness
Ma03_g15580	I-box	part of a light responsive element
Ma03_g15580	I-box	part of a light responsive element
Ma03_g15580	I-box	part of a light responsive element
Ma03_g15580	ATCT-motif	part of a conserved DNA module involved in light responsiveness
Ma03_g15580	CGTCA-motif	cis-acting regulatory element involved in the MeJA-responsiveness
Ma03_g15580	LAMP-element	part of a light responsive element
Ma03_g15580	TGACG-motif	cis-acting regulatory element involved in the MeJA-responsiveness
Ma03_g15580	TCA-element	cis-acting element involved in salicylic acid responsiveness
Ma03_g15580	Box 4	part of a conserved DNA module involved in light responsiveness
Ma06_g26840	G-box	cis-acting regulatory element involved in light responsiveness
Ma06_g26840	HD-Zip 1	element involved in differentiation of the palisade mesophyll cells
Ma06_g26840	MBS	MYB binding site involved in drought-inducibility
Ma06_g26840	TGA-element	auxin-responsive element
Ma06_g26840	ATCT-motif	part of a conserved DNA module involved in light responsiveness
Ma06_g26840	TGACG-motif	cis-acting regulatory element involved in the MeJA-responsiveness
Ma06_g26840	TGACG-motif	cis-acting regulatory element involved in the MeJA-responsiveness
Ma06_g26840	TCA-element	cis-acting element involved in salicylic acid responsiveness
Ma06_g26840	A-box	cis-acting regulatory element
Ma06_g26840	CGTCA-motif	cis-acting regulatory element involved in the MeJA-responsiveness
Ma06_g26840	CGTCA-motif	cis-acting regulatory element involved in the MeJA-responsiveness
Ma06_g26840	CGTCA-motif	cis-acting regulatory element involved in the MeJA-responsiveness
Ma06_g26840	CGTCA-motif	cis-acting regulatory element involved in the MeJA-responsiveness
Ma06_g26850	TGACG-motif	cis-acting regulatory element involved in the MeJA-responsiveness
Ma06_g26850	TGACG-motif	cis-acting regulatory element involved in the MeJA-responsiveness
Ma06_g26850	TGACG-motif	cis-acting regulatory element involved in the MeJA-responsiveness
Ma06_g26850	A-box	cis-acting regulatory element
Ma06_g26850	AuxRR-core	cis-acting regulatory element involved in auxin responsiveness
Ma06_g26850	MBS	MYB binding site involved in drought-inducibility

Ma06_g26850	ACA-motif	part of gapA in (gapA-CMA1) involved with light responsiveness
Ma06_g26850	G-box	cis-acting regulatory element involved in light responsiveness
Ma06_g26850	CGTCA-motif	cis-acting regulatory element involved in the MeJA-responsiveness
Ma06_g26850	CGTCA-motif	cis-acting regulatory element involved in the MeJA-responsiveness
Ma06_g26850	CGTCA-motif	cis-acting regulatory element involved in the MeJA-responsiveness
Ma06_g26850	CGTCA-motif	cis-acting regulatory element involved in the MeJA-responsiveness
Ma06_g26850	CGTCA-motif	cis-acting regulatory element involved in the MeJA-responsiveness
Ma06_g26850	HD-Zip 1	element involved in differentiation of the palisade mesophyll cells
Ma06_g26850	TGA-element	auxin-responsive element
Ma06_g26870	TCA-element	cis-acting element involved in salicylic acid responsiveness
Ma06_g26870	G-Box	cis-acting regulatory element involved in light responsiveness
Ma06_g26870	TCT-motif	part of a light responsive element
Ma06_g26870	GT1-motif	light responsive element
Ma06_g26870	GA-motif	part of a light responsive element
Ma06_g26870	ARE	cis-acting regulatory element essential for the anaerobic induction
Ma06_g26870	ARE	cis-acting regulatory element essential for the anaerobic induction
Ma06_g26870	I-box	part of a light responsive element
Ma06_g26890	AT-rich element	binding site of AT-rich DNA binding protein (ATBP-1)
Ma06_g26890	ARE	cis-acting regulatory element essential for the anaerobic induction
Ma06_g26890	ARE	cis-acting regulatory element essential for the anaerobic induction
Ma06_g26890	GCN4_motif	cis-regulatory element involved in endosperm expression
Ma06_g26890	TCT-motif	part of a light responsive element
Ma06_g26890	GT1-motif	light responsive element
Ma06_g26890	MRE	MYB binding site involved in light responsiveness
Ma06_g26890	G-box	cis-acting regulatory element involved in light responsiveness
Ma06_g30170	G-box	cis-acting regulatory element involved in light responsiveness
Ma06_g30170	G-box	cis-acting regulatory element involved in light responsiveness
Ma06_g30170	G-box	cis-acting regulatory element involved in light responsiveness
Ma06_g30170	G-box	cis-acting regulatory element involved in light responsiveness
Ma06_g30170	G-box	cis-acting regulatory element involved in light responsiveness
Ma06_g30170	G-box	cis-acting regulatory element involved in light responsiveness
Ma06_g30170	G-box	cis-acting regulatory element involved in light responsiveness
Ma06_g30170	G-box	cis-acting regulatory element involved in light responsiveness
Ma06_g30170	O2-site	cis-acting regulatory element involved in zein metabolism regulation
Ma06_g30170	O2-site	cis-acting regulatory element involved in zein metabolism regulation
Ma06_g30170	TGA-element	auxin-responsive element
Ma06_g30170	Box 4	part of a conserved DNA module involved in light responsiveness
Ma06_g30170	Box 4	part of a conserved DNA module involved in light responsiveness
Ma06_g30170	Box 4	part of a conserved DNA module involved in light responsiveness
Ma06_g30170	TCT-motif	part of a light responsive element
Ma06_g30170	TGACG-motif	cis-acting regulatory element involved in the MeJA-responsiveness
Ma06_g30170	TGACG-motif	cis-acting regulatory element involved in the MeJA-responsiveness

Ma06_g30170	TGACG-motif	cis-acting regulatory element involved in the MeJA-responsiveness
Ma06_g30170	I-box	part of a light responsive element
Ma06_g30170	Box II	part of a light responsive element
Ma06_g30170	G-Box	cis-acting regulatory element involved in light responsiveness
Ma06_g30170	G-Box	cis-acting regulatory element involved in light responsiveness
Ma06_g30170	G-Box	cis-acting regulatory element involved in light responsiveness
Ma06_g30170	G-Box	cis-acting regulatory element involved in light responsiveness
Ma07_g09040	GATA-motif	part of a light responsive element
Ma07_g09040	ARE	cis-acting regulatory element essential for the anaerobic induction
Ma07_g09040	LTR	cis-acting element involved in low-temperature responsiveness
Ma07_g09040	TGACG-motif	cis-acting regulatory element involved in the MeJA-responsiveness
Ma07_g09040	TGACG-motif	cis-acting regulatory element involved in the MeJA-responsiveness
Ma07_g09040	GT1-motif	light responsive element
Ma07_g09040	GT1-motif	light responsive element
Ma07_g09040	GT1-motif	light responsive element
Ma07_g09040	GA-motif	part of a light responsive element
Ma07_g09040	AT-rich element	binding site of AT-rich DNA binding protein (ATBP-1)
Ma07_g09040	TCT-motif	part of a light responsive element
Ma07_g09040	TCT-motif	part of a light responsive element
Ma07_g09040	Box 4	part of a conserved DNA module involved in light responsiveness
Ma07_g09040	Box 4	part of a conserved DNA module involved in light responsiveness
Ma07_g09040	Box 4	part of a conserved DNA module involved in light responsiveness
Ma07_g09040	Box 4	part of a conserved DNA module involved in light responsiveness
Ma07_g11670	LTR	cis-acting element involved in low-temperature responsiveness
Ma07_g11670	LTR	cis-acting element involved in low-temperature responsiveness
Ma07_g11670	TCT-motif	part of a light responsive element
Ma07_g11670	G-box	cis-acting regulatory element involved in light responsiveness
Ma07_g11670	G-box	cis-acting regulatory element involved in light responsiveness
Ma07_g11670	GATA-motif	part of a light responsive element
Ma07_g11670	ARE	cis-acting regulatory element essential for the anaerobic induction
Ma07_g11670	ARE	cis-acting regulatory element essential for the anaerobic induction
Ma07_g11670	ARE	cis-acting regulatory element essential for the anaerobic induction
Ma07_g11670	G-Box	cis-acting regulatory element involved in light responsiveness
Ma07_g11670	TGACG-motif	cis-acting regulatory element involved in the MeJA-responsiveness
Ma07_g11670	AT1-motif	part of a light responsive module
Ma08_g14590	ARE	cis-acting regulatory element essential for the anaerobic induction
Ma08_g14590	ARE	cis-acting regulatory element essential for the anaerobic induction
Ma08_g14590	ARE	cis-acting regulatory element essential for the anaerobic induction
Ma08_g14590	ARE	cis-acting regulatory element essential for the anaerobic induction
Ma08_g14590	ARE	cis-acting regulatory element essential for the anaerobic induction
Ma08_g14590	CGTCA-motif	cis-acting regulatory element involved in the MeJA-responsiveness
Ma08_g14590	TCCC-motif	part of a light responsive element

Ma08_g14590	AE-box	part of a module for light response
Ma08_g14590	G-box	cis-acting regulatory element involved in light responsiveness
Ma08_g14590	G-Box	cis-acting regulatory element involved in light responsiveness
Ma08_g14590	G-Box	cis-acting regulatory element involved in light responsiveness
Ma08_g14590	MRE	MYB binding site involved in light responsiveness
Ma08_g14590	LAMP-element	part of a light responsive element
Ma08_g14590	GCN4_motif	cis-regulatory element involved in endosperm expression
Ma08_g14590	TCA-element	cis-acting element involved in salicylic acid responsiveness
Ma08_g23400	TGACG-motif	cis-acting regulatory element involved in the MeJA-responsiveness
Ma08_g23400	P-box	gibberellin-responsive element
Ma08_g23400	G-box	cis-acting regulatory element involved in light responsiveness
Ma08_g23400	G-box	cis-acting regulatory element involved in light responsiveness
Ma08_g23400	G-box	cis-acting regulatory element involved in light responsiveness
Ma08_g23400	G-Box	cis-acting regulatory element involved in light responsiveness
Ma08_g23400	TGA-element	auxin-responsive element
Ma08_g23400	TGA-element	auxin-responsive element
Ma08_g23400	Box 4	part of a conserved DNA module involved in light responsiveness
Ma08_g23400	Box 4	part of a conserved DNA module involved in light responsiveness
Ma08_g23400	A-box	cis-acting regulatory element
Ma09_g12090	MBS	MYB binding site involved in drought-inducibility
Ma09_g12090	MRE	MYB binding site involved in light responsiveness
Ma09_g12090	ARE	cis-acting regulatory element essential for the anaerobic induction
Ma09_g12090	ARE	cis-acting regulatory element essential for the anaerobic induction
Ma09_g12090	G-box	cis-acting regulatory element involved in light responsiveness
Ma09_g12090	G-box	cis-acting regulatory element involved in light responsiveness
Ma09_g12090	G-box	cis-acting regulatory element involved in light responsiveness
Ma09_g12090	G-box	cis-acting regulatory element involved in light responsiveness
Ma09_g12090	G-box	cis-acting regulatory element involved in light responsiveness
Ma09_g12090	G-Box	cis-acting regulatory element involved in light responsiveness
Ma09_g12090	Sp1	light responsive element
Ma09_g12090	TATC-box	cis-acting element involved in gibberellin-responsiveness
Ma09_g12090	TCA-element	cis-acting element involved in salicylic acid responsiveness
Ma09_g12090	TCA-element	cis-acting element involved in salicylic acid responsiveness
Ma09_g12090	AE-box	part of a module for light response
Ma09_g15420	LTR	cis-acting element involved in low-temperature responsiveness
Ma09_g15420	A-box	cis-acting regulatory element
Ma09_g15420	A-box	cis-acting regulatory element
Ma09_g15420	G-Box	cis-acting regulatory element involved in light responsiveness
Ma09_g15420	G-Box	cis-acting regulatory element involved in light responsiveness
Ma09_g15420	ARE	cis-acting regulatory element essential for the anaerobic induction
Ma09_g15420	HD-Zip 1	element involved in differentiation of the palisade mesophyll cells
Ma09_g15420	MBS	MYB binding site involved in drought-inducibility

Ma09_g15420	G-box	cis-acting regulatory element involved in light responsiveness
Ma09_g15420	G-box	cis-acting regulatory element involved in light responsiveness
Ma09_g15420	G-box	cis-acting regulatory element involved in light responsiveness
Ma09_g15420	G-box	cis-acting regulatory element involved in light responsiveness
Ma09_g15420	G-box	cis-acting regulatory element involved in light responsiveness
Ma09_g15420	G-box	cis-acting regulatory element involved in light responsiveness
Ma09_g15420	TATC-box	cis-acting element involved in gibberellin-responsiveness
Ma09_g15420	CGTCA-motif	cis-acting regulatory element involved in the MeJA-responsiveness
Ma09_g15420	TCT-motif	part of a light responsive element
Ma09_g15420	TGA-element	auxin-responsive element
Ma09_g15420	TCCC-motif	part of a light responsive element
Ma09_g15420	ACE	cis-acting element involved in light responsiveness
Ma09_g15420	TGACG-motif	cis-acting regulatory element involved in the MeJA-responsiveness
Ma09_g15420	TGACG-motif	cis-acting regulatory element involved in the MeJA-responsiveness
Ma09_g15420	AE-box	part of a module for light response
Ma10_g17560	HD-Zip 1	element involved in differentiation of the palisade mesophyll cells
Ma10_g17560	AE-box	part of a module for light response
Ma10_g17560	G-Box	cis-acting regulatory element involved in light responsiveness
Ma10_g17560	ATCT-motif	part of a conserved DNA module involved in light responsiveness
Ma10_g17560	AT1-motif	part of a light responsive module
Ma10_g17560	AT-rich element	binding site of AT-rich DNA binding protein (ATBP-1)
Ma10_g17560	LTR	cis-acting element involved in low-temperature responsiveness
Ma10_g17560	TGACG-motif	cis-acting regulatory element involved in the MeJA-responsiveness
Ma10_g17560	TGACG-motif	cis-acting regulatory element involved in the MeJA-responsiveness
Ma10_g25490	TGA-element	auxin-responsive element
Ma10_g25490	G-box	cis-acting regulatory element involved in light responsiveness
Ma10_g25490	G-Box	cis-acting regulatory element involved in light responsiveness
Ma10_g25490	G-Box	cis-acting regulatory element involved in light responsiveness
Ma10_g25490	GC-motif	enhancer-like element involved in anoxic specific inducibility
Ma10_g25490	GC-motif	enhancer-like element involved in anoxic specific inducibility
Ma10_g25490	GARE-motif	gibberellin-responsive element
Ma10_g25490	ARE	cis-acting regulatory element essential for the anaerobic induction
Ma10_g25490	ARE	cis-acting regulatory element essential for the anaerobic induction
Ma10_g25490	ARE	cis-acting regulatory element essential for the anaerobic induction
Ma10_g25490	DRE	cis-acting element involved in dehydration, low-temp, salt stresses
Ma10_g25490	I-box	part of a light responsive element
Ma10_g25490	CGTCA-motif	cis-acting regulatory element involved in the MeJA-responsiveness
Ma10_g25490	CGTCA-motif	cis-acting regulatory element involved in the MeJA-responsiveness
Ma10_g25490	ABRE	cis-acting element involved in the abscisic acid responsiveness
Ma10_g25490	ABRE	cis-acting element involved in the abscisic acid responsiveness
Ma10_g25490	MSA-like	cis-acting element involved in cell cycle regulation
Ma10_g25490	GTGGC-motif	part of a light responsive element

Table S7. TF-binding motifs in the promoter regions of *LOX* genes.

Genes name	TFs	Sequence
Ma01_g16400	MIKC_MADS	TTTCATTTTTTTTTGTTTTGC
Ma01_g16400	G2-like	ATAATAATATTCTTA
Ma01_g16400	TALE	TCCTCTCTTCCTCTCTCGCT
Ma01_g16400	BBR-BPC	CTCTTCCTCTCTCTTTCAAAA
Ma01_g16400	BBR-BPC	CCATCACTCTTCCTCTCTCTT
Ma01_g16400	C2H2	CCTCCAACACATCCTCATC
Ma01_g16400	BBR-BPC	GATGGAGAAAGATGGAAGAAGATA
Ma01_g16400	BBR-BPC	AGGATGGAGAAAGATGGAAGAAGA
Ma01_g16400	BBR-BPC	CTTCCTCTCTTCCTCTCTCGC
Ma01_g16400	MYB	TTGGGTTGGTGTATC
Ma01_g16400	BBR-BPC	GTTACTTCCTCTCTTCCTCTC
Ma01_g16400	BBR-BPC	TCCTCTCTTCCTCTCTCGCTT
Ma01_g16400	C2H2	TATCCTCCAACACATCCTC
Ma01_g16400	BBR-BPC	TACTTCCTCTCTTCCTCTCTC
Ma01_g16400	bHLH	TCACGTGG
Ma01_g16400	TALE	CTTCCTCTCTTCCTCTCTCG
Ma01_g16400	YABBY	TAATAATAAT
Ma01_g16400	MYB	TTGGATTGATTAGGT
Ma01_g16400	bHLH	CACGTGGTTCACATG
Ma01_g16400	B3	GTTGAGTGTTTGGAGGAAAAC
Ma01_g16400	WRKY	AATAGTCAAAGAT
Ma01_g16400	TCP	AGTGGGTCCACCATGGGGTCCATATGATGT
Ma01_g16400	WRKY	AATAGTCAAAGAT
Ma01_g16400	HD-ZIP	TGTAATCATTT
Ma01_g16400	TALE	CTCTCTTCCTCTCTCGCTTG
Ma01_g16400	Dof	CAAATGATAAAGCAAATTCAT
Ma01_g16400	TCP	ATGGGGTCCATAT
Ma01_g16400	TCP	GGGACCAT
Ma01_g16400	TCP	AAGTGGGTCCACC
Ma01_g16400	BBR-BPC	TTGTTACTTCCTCTCTTCCTC
Ma01_g16400	C2H2	CTTTTTCCACATCTTCTTT
Ma01_g16400	bZIP	TCACGTGG
Ma01_g16400	WRKY	AATAGTCAAAGAT
Ma01_g16400	HD-ZIP	ACATTTAATGT
Ma01_g16400	BBR-BPC	CTCTCTTCCTCTCTCGCTTGG
Ma01_g16400	Dof	TTCATTTTTTTTTGTTTTGCA
Ma01_g16400	bZIP	GGAGACGACGTCACG
Ma01_g16400	MYB	TGGGTTGGTGT
Ma01_g16400	HSF	GAATTTTCTATA

Ma01_g16400	HSF	GAATTTTCTATA
Ma01_g16400	CPP	TATTTGAATA
Ma01_g16400	bZIP	GGAGACGACGTCACG
Ma01_g16400	HD-ZIP	GAAAGAGATGATTAC
Ma01_g16400	bZIP	ATGAGCTGTCA
Ma01_g16400	bZIP	ATGAGCTGTCA
Ma01_g16400	MYB	GAGATGGATAAGA
Ma01_g16400	CPP	AGTTTGAATC
Ma01_g16400	bZIP	ACGTCACGTGGT
Ma01_g16400	bZIP	ACGTCACGTGGT
Ma01_g16400	bZIP	ACGTCACGTGGT
Ma01_g16400	TCP	GTGGGTCCACC
Ma01_g16400	HD-ZIP	GCAATAATTGC
Ma01_g16400	YABBY	TTATAATAAT
Ma01_g16400	bHLH	TCACATGG
Ma01_g16400	WRKY	ATAGTCAAAG
Ma01_g16400	bZIP	CCATATGATGTGGCC
Ma01_g16400	HSF	TTATGAATTTTCTATAAAA
Ma01_g16400	BBR-BPC	TCCCATCACTCTTCCTCTCTC
Ma01_g16400	bHLH	ACGACGTCACGTGGTTCACAT
Ma01_g16400	C2H2	CAACTTTTTCCACATCTTC
Ma01_g16400	G2-like	CAAAGAATAATCTT
Ma01_g16400	WRKY	TCTTGACTTATGA
Ma01_g16400	HD-ZIP	TGTAATCATT
Ma01_g16400	MYB	AGATATTGTGTGTTTGGTACT
Ma01_g16400	G2-like	GGTATATTCGT
Ma01_g16400	C3H	AATGATAAAGCAAA
Ma01_g16400	HD-ZIP	ACATTTAATGT
Ma01_g16400	MYB	CATCCTCACCTAATATTGC
Ma01_g16400	HD-ZIP	TAATCATT
Ma01_g16400	MIKC_MADS	CATCATC
Ma01_g16400	MIKC_MADS	CATCATC
Ma01_g16400	MIKC_MADS	CATCATC
Ma01_g16400	Dof	GTCCAAATGATAAAGCAAATT
Ma01_g16400	MYB	ATGGTAGAAGTGGGTCCACCA
Ma01_g16400	HD-ZIP	GCAATAATTGC
Ma01_g16400	TCP	AGTGGGTCCACCA
Ma01_g16400	MYB	GCTTGGGTTGGTGTA
Ma01_g16400	HSF	AGAAAGACCAAGAAG
Ma01_g16400	TCP	GGGTCCAC
Ma01_g16400	G2-like	AGTACATTATTCCTT
Ma01_g16400	HD-ZIP	TGGTCAATTATTGTTTAGTGT

Ma01_g16400	bHLH	TGGCATGTGA
Ma01_g16400	AP2	ATGGAGAAAGATGGAAGAAG
Ma01_g16400	TCP	GTGGGTCCAC
Ma01_g16400	MYB_related	GAGATATTG
Ma01_g16400	MYB	TGGGTTGGTGTAT
Ma01_g16400	C2H2	TCATTTTTTTTTGTTTTGCA
Ma01_g16400	bHLH	CACGTGGTTCACAT
Ma01_g16400	bHLH	CACGTGGTTCACAT
Ma01_g16400	Dof	TTATTTTTATGAATTTTCTAT
Ma01_g16400	GATA	ATCTAATATGATCGTTCTAATGTTCAA
Ma01_g16400	TCP	GGGACCAA
Ma01_g16400	MIKC_MADS	CTTTTTCCACATCTTCTTTTA
Ma01_g16400	C2H2	TGAAACAAAGAA
Ma01_g16400	bHLH	CCAACTTGT
Ma01_g16400	BBR-BPC	AATGAGGGTTAAGTTAGAGAGAGA
Ma01_g16400	ERF	TGCGTCGCCTCCAACATGGCA
Ma01_g16400	C2H2	TCCTCTCTTCCTCTCTCGC
Ma01_g16400	TCP	GTGGGTCCAC
Ma01_g16400	WRKY	TATCTTGACTTATGA
Ma01_g16400	C2H2	AAGAGCAGAGAG
Ma01_g16400	HD-ZIP	ATTGCAATAATTGCTAAGGGA
Ma01_g16400	BBR-BPC	GAAGAGCAGAGAGATGGATAAGAA
Ma01_g16400	HD-ZIP	CAATAATT
Ma01_g16400	Dof	GAGAAAGATGGAAGAAGATAT
Ma01_g16400	Dof	GAGAAAGATGGAAGAAGATAT
Ma01_g18020	BBR-BPC	GAGAGAGAGACAGAAGTAATGGTG
Ma01_g18020	BBR-BPC	AGGAGAGAGAGACAGAAGTAATGG
Ma01_g18020	CPP	CGAAATTTCAAAC
Ma01_g18020	Dof	AAAGAAATAAAGCAAGAGCAT
Ma01_g18020	MYB_related	AAGATATTT
Ma01_g18020	AP2	TATAACAGGAGAGAGAGACA
Ma01_g18020	HD-ZIP	GAACAAATAATTTAAACTATC
Ma01_g18020	CPP	TCAAATTTGAAAA
Ma01_g18020	TCP	GGGGCCCAT
Ma01_g18020	C2H2	TGCGAAACAACAAAACA
Ma01_g18020	bHLH	CACGTGATATTTTT
Ma01_g18020	bHLH	CACGTGATATTTTT
Ma01_g18020	B3	CTAGGCATGCATTTA
Ma01_g18020	HD-ZIP	ATAATAATTTA
Ma01_g18020	bHLH	GCACATGC
Ma01_g18020	MYB	TGATTGAAATTAGGC
Ma01_g18020	AP2	TAACAGGAGAGAGAGACAGA

Ma01_g18020	HD-ZIP	ATAATAATTTA
Ma01_g18020	WRKY	TTTATTGACTTTGCA
Ma01_g18020	HD-ZIP	TAATAATT
Ma01_g18020	WRKY	TTTATTGACTTTG
Ma01_g18020	Dof	TGAAAAGAAATAAAGCAAGAG
Ma01_g18020	Dof	TGAAAAGAAATAAAGCAAGAG
Ma01_g18020	WRKY	TATTGACTTTGCA
Ma01_g18020	HD-ZIP	CAAATAATTTA
Ma01_g18020	C2H2	TGCGAAACAACAAAACAT
Ma01_g18020	Dof	TGAAAAGAAATAAAGCAAGAG
Ma01_g18020	TCP	AGGGGCCCAT
Ma01_g18020	BES1	CACACGTGATA
Ma01_g18020	MIKC_MADS	TTTAATCTACTTTTAGATA
Ma01_g18020	MIKC_MADS	TTTAATCTACTTTTAGATA
Ma01_g18020	MYB	GATTGAAATTAGGCC
Ma01_g18020	C2H2	TACCCAAAGCACATA
Ma01_g18020	TCP	AGGGGGGCCAACAGAGCATCGCAGACTCTA
Ma01_g18020	MYB_related	AAGATATTT
Ma01_g18020	C2H2	AGCTTCACCGTATAA
Ma01_g18020	BES1	ACACACGTGATATTTTTTAA
Ma01_g18020	AP2	CAAAGAATAAATACAAAACA
Ma01_g18020	HD-ZIP	CAAATAATTTA
Ma01_g18020	MYB	ATTGAAATTAGGCCA
Ma01_g18020	MYB	ATTGAAATTAGGCCA
Ma01_g18020	MIKC_MADS	ATCCCTTCATTTTTGGCAA
Ma01_g18020	MIKC_MADS	ATCCCTTCATTTTTGGCAA
Ma01_g18020	Trihelix	GAATGACGGAGTCA
Ma01_g18020	NAC	CAATGGCGTATAAAA
Ma01_g18020	C2H2	CACAAACACTA
Ma01_g18020	Dof	AATAAAGCAAGAGCATACAAG
Ma01_g18020	Dof	AATAAAGCAAGAGCATACAAG
Ma01_g18020	BBR-BPC	CGTATAACAGGAGAGAGAGACAGA
Ma01_g18020	bHLH	GCACATGC
Ma01_g18020	TCP	TAGGGGCCCATTA
Ma01_g18020	C3H	GAACGAAAAGGAAC
Ma01_g18020	BES1	ACACACGTGATATTT
Ma01_g18020	HD-ZIP	GATATAATAATTTATAGATAT
Ma01_g18020	MYB	TCCATCCACCCACATCTGG
Ma01_g18020	Dof	AAATTTGAAAAGAAATAAAGC
Ma01_g18020	C2H2	ACACTAGAGTACAAA
Ma01_g18020	WRKY	TTTATTGACTTTGC
Ma01_g18020	G2-like	AGAATCTA

Ma01_g18020	MYB	AGAGTTATAGATAATTTGATA
Ma01_g18020	LFY	GGATGACCACTAGACAAAA
Ma01_g18020	CPP	ATCAATTTTAAAT
Ma01_g18020	bHLH	ACACGTGA
Ma01_g18020	Dof	CAGAACGAAAAGGAACAGTTT
Ma01_g18020	Dof	AAATGGAATAAAGAGTAAT
Ma01_g18060	ERF	CGGTGCCGCC
Ma01_g18060	BBR-BPC	CTTTTTTTTTCTCTATGACTC
Ma01_g18060	GATA	ACGGTGGCGCTGATG
Ma01_g18060	BBR-BPC	ATTTTTTTCTTTCTTTCTCAT
Ma01_g18060	MIKC_MADS	TTTTTTTCTTTCTTTCTCATC
Ma01_g18060	ERF	CGCCGCCG
Ma01_g18060	Dof	TAAATTTTTTTCTTTCTTTCT
Ma01_g18060	ERF	CCGCCGCCGA
Ma01_g18060	MIKC_MADS	TTTCTTTCTTTCTCATCCTTA
Ma01_g18060	ERF	GTCGCCGACGGTGGC
Ma01_g18060	ERF	CGACGGTGGCGCTGA
Ma01_g18060	ERF	CCCGCCGCCGACGATGTGACT
Ma01_g18060	BBR-BPC	TTAAATTTTTTTCTTTCTTTC
Ma01_g18060	BBR-BPC	TTTTCTTTCTTTCTCATCCTT
Ma01_g18060	bZIP	TTCAGAACACGTGTCCGT
Ma01_g18060	NAC	TATTGTCTTGATTCCCACG
Ma01_g18060	ERF	GTCGATGACGGTGCC
Ma01_g18060	Dof	AAAAGATGAAAAGGGTGAA
Ma01_g18060	bZIP	TCAGAACACGTGTCC
Ma01_g18060	ERF	GGTCGCCGACGGTGGCGCT
Ma01_g18060	MYB_related	AAGATATTT
Ma01_g18060	Dof	TTTTCTTTCTTTCTCATCCTT
Ma01_g18060	BBR-BPC	GAGGAAGATTGAGATAAAAGATGA
Ma01_g18060	HD-ZIP	TTAATAATTAT
Ma01_g18060	HD-ZIP	TTAATAATTAT
Ma01_g18060	Dof	CATAAAGTTAC
Ma01_g18060	Dof	ATATCCTTTTTTTTTCTCTAT
Ma01_g18060	RAV	TATGTTGC
Ma01_g18060	NAC	CTTGATTCCCACG
Ma01_g18060	bHLH	TCACGTGC
Ma01_g18060	TCP	GGGACCAT
Ma01_g18060	Dof	CTTTCTTTCTCATCCTTATTT
Ma01_g18060	ERF	CCGACGGTGGCGCTG
Ma01_g18060	ERF	CCGACGGTGGCGCTG
Ma01_g18060	G2-like	GAATATTC
Ma01_g18060	G2-like	GAATATTC

Ma01_g18060	bHLH	TCACGTGC
Ma01_g18060	ERF	GACCCGCCGCCGACG
Ma01_g18060	BBR-BPC	GGGTCAAAGAGGAAGATTGAGATA
Ma01_g18060	ERF	CGATGACGGTGCCGCCG
Ma01_g18060	ERF	TGCGACCCGCCGCCGACGATG
Ma01_g18060	ERF	GACCCGCCGCCGACGATGTGA
Ma01_g18060	ERF	GACCCGCCGCCGACGATGTGA
Ma01_g18060	Dof	TATCCTTTTTTTTTCTCTATG
Ma01_g18060	ARF	TTTGTCGGG
Ma01_g18060	NAC	CGATTACGTGAAGAA
Ma01_g18060	ERF	CCATGCGACCCGCCGCCGAC
Ma01_g18060	bHLH	ACCCATGTGA
Ma01_g18060	C2H2	TTTCTTTCTCATCCTTATT
Ma01_g18060	ERF	TGACGGTGCCGCCGA
Ma01_g18060	ERF	GTCGCCGACGGTGGC
Ma01_g18060	MYB	TGAGAAGATAAGA
Ma01_g18060	MIKC_MADS	CCTTTTTTTTTCTCTATGACT
Ma01_g18060	ERF	CGCCGACGGTGGCGCTGAT
Ma01_g18060	Dof	GAATACAGCATAAAGTTACAT
Ma01_g18060	Dof	CATAATAAAAAGCAGTAATAT
Ma01_g18060	HD-ZIP	TAATAATT
Ma01_g18060	HD-ZIP	TAATAATT
Ma01_g18060	Dof	TAAAAAGATGAAAAGGGTGAA
Ma01_g18060	Dof	TAAAAAGATGAAAAGGGTGAA
Ma01_g18060	ERF	GACCCGCCGCCGACG
Ma01_g18060	AP2	TCATCGTACA
Ma01_g18060	ERF	GATGACGGTGCCGC
Ma01_g18060	ERF	GATGACGGTGCCGC
Ma01_g18060	C3H	CAATAAAAAGGTAC
Ma01_g18060	ERF	TGCGACCCGCCGCCGACGATGTGACTCA
Ma01_g18060	HD-ZIP	TTAATAATTAT
Ma01_g18060	HD-ZIP	TTAATAATTAT
Ma01_g18060	ARF	TTGTCGGGTC
Ma01_g18060	ARF	TTGTCGGGTC
Ma01_g18060	G2-like	GCTAGATTCGATCG
Ma01_g18060	C3H	CATAAAGTTAC
Ma01_g18060	SBP	CTTGTACGTAA
Ma01_g18060	bHLH	TCACGTGC
Ma01_g18060	MIKC_MADS	TAACATAAAATGGGAAAAA
Ma01_g18060	BBR-BPC	CAAAGAGGAAGATTGAGATAAAAG
Ma01_g18060	Dof	ACACATAATAAAAAGCAGTAA
Ma01_g18060	ERF	TGTCGATGACGGTGCCGCC

Ma01_g18060	BBR-BPC	AAATTTTTTTCTTTCTTTCTC
Ma01_g18060	bZIP	ATAGGCTGGCA
Ma01_g18060	bZIP	ATAGGCTGGCA
Ma01_g18060	TCP	AAGGGGACCATGA
Ma01_g18060	WOX	TCATTCATGTA
Ma01_g18060	MYB	GTGTGTGGTGG
Ma01_g18060	bHLH	TCACGTGC
Ma01_g18060	C3H	TAAAAAGGTAC
Ma01_g18060	bHLH	ACACGTGT
Ma01_g18060	bZIP	TCAGAACACGTGTCC
Ma01_g18060	NAC	CTTGATTCCCACGCC
Ma01_g18060	bHLH	CACGTGCTTTGTTT
Ma01_g18060	bHLH	CACGTGCTTTGTTT
Ma01_g18060	bZIP	ATCGATTACGTGAAG
Ma01_g18060	MYB_related	AAGATATTT
Ma01_g18060	YABBY	TATGATAA
Ma01_g18060	YABBY	TATGATAA
Ma01_g18060	Dof	TTGAGATAAAAGATGAAAAAA
Ma01_g18060	NAC	ATTGTCTTGATTCCCACGCCG
Ma01_g18060	ERF	AGGGTCGCCGACGGTGGCGCT
Ma01_g18060	Dof	TAAGGCGGTAAAAAGATGAAA
Ma01_g18060	Dof	CATAAAGTTACATG
Ma01_g18060	bHLH	ACACGTGT
Ma01_g18060	Dof	ATAAAAGATGAAAAAAATTCT
Ma01_g18060	Dof	ATAAAAGATGAAAAAAATTCT
Ma01_g18060	ERF	CGACCCGCCGCCGACGATGTG
Ma01_g18060	bZIP	ACACGTGTCC
Ma01_g18060	TCP	AGGGGACCAT
Ma01_g18060	MYB	AAGATATTTAGGTTA
Ma01_g18060	MYB	AAGATATTTAGGTTA
Ma01_g18060	bHLH	ACACGTGT
Ma01_g18060	C2H2	AGAGACAGTGG
Ma01_g18060	BBR-BPC	TTTTTTTTCTCTATGACTCTA
Ma01_g18060	Dof	TAAAAAGGTACATG
Ma01_g18060	MYB	CATTGAGAAGATAAGAGTCCA
Ma01_g18060	MIKC_MADS	ATTCTATTATTTTTGGTTA
Ma01_g18060	MIKC_MADS	ATTCTATTATTTTTGGTTA
Ma01_g18060	ERF	TCGCCGACGGTGGCG
Ma01_g18060	ERF	TCGCCGACGGTGGCG
Ma01_g18060	Dof	TTAAATTTTTTTCTTTCTTTC
Ma01_g18060	Dof	AAAAAGATGAAAAGGGTGAAT
Ma01_g18060	bHLH	CACGTGTCCGTTCA

Ma01_g18060	bHLH	CACGTGTCCGTTCA
Ma01_g18060	bHLH	CACGTGCTTTGTTTA
Ma01_g18060	Trihelix	TGATGTAGAGACCCTAAT
Ma01_g18060	C3H	CAGCATAAAGTTAC
Ma01_g18060	Dof	TAAAAAGGTACATGTGGGAAT
Ma01_g18060	Dof	TAAAAAGGTACATGTGGGAAT
Ma01_g18060	NAC	TACGTAATGCAGTCTCTCCCTT
Ma01_g18060	bHLH	TTCAGAACACGTGT
Ma01_g18060	WOX	TTTCAATCAC
Ma01_g18060	ERF	CGTTGTCGATGACGGTGCCGC
Ma01_g18060	NAC	TACGTAAT
Ma01_g18060	bZIP	ATCGATTACGTGAAG
Ma01_g18060	HD-ZIP	GATTTAATAATTATATTTAAT
Ma01_g18060	MYB_related	AAGATACTT
Ma01_g18060	Dof	TAAAAAGGTAC
Ma01_g18060	ERF	TCGATGACGGTGCCG
Ma01_g18060	ERF	TCGATGACGGTGCCG
Ma01_g18060	Trihelix	ATATTTACTTTATA
Ma01_g18060	MYB_related	TCCCTTATCTTAAAC
Ma01_g18060	HD-ZIP	AAAATTAATTATTAT
Ma01_g18060	bHLH	ACACGTGT
Ma01_g18060	bHLH	TCACGTGC
Ma01_g18060	WOX	ATTCAATCAC
Ma01_g18060	WRKY	GGGTCAAA
Ma01_g18060	Dof	AAGATAGAAAAGGATTTAATA
Ma01_g18060	BBR-BPC	AAGAGGAAGATTGAGATAAAAGAT
Ma01_g18060	ERF	TGTGCTGAGGTGTGTGGTGGA
Ma01_g18060	bHLH	ATCACGTGCT
Ma01_g18060	HD-ZIP	AAATTAATTAT
Ma01_g18060	MIKC_MADS	GTCAAAGAGGAAGA
Ma01_g18060	BBR-BPC	TCCTTTTTTTTCTCTATGAC
Ma01_g18060	C2H2	CATTTATTTGTCATATATCA
Ma01_g18060	HD-ZIP	ATTAATTATTA
Ma01_g18060	G2-like	CGAATATTCAA
Ma01_g18060	ERF	CGGTGGCGCTGATGA
Ma02_g07800	MIKC_MADS	TCTCTTTTTTTTTTTTTTTG
Ma02_g07800	Dof	CTCTCTTTTTTTTTTTTTTT
Ma02_g07800	MIKC_MADS	GCTCTCTTTTTTTTTTTTTT
Ma02_g07800	MIKC_MADS	CTTCTTCTTCTTCTTCTTCTT
Ma02_g07800	MIKC_MADS	CTCTTTTTTTTTTTTTTTGA
Ma02_g07800	C2H2	CTTCTTCTTCTTCTTCTTC
Ma02_g07800	MIKC_MADS	CTTCTCCTTCTTCTTCTTCTT

Ma02_g07800	MIKC_MADS	CGCTCTCTTTTTTTTTTTTTT
Ma02_g07800	MIKC_MADS	CTCCTTCTTCTTCTTCTTCTT
Ma02_g07800	C2H2	CTCCTTCTTCTTCTTCTTC
Ma02_g07800	TCP	ATGCGGGCCCCAC
Ma02_g07800	C2H2	CTTCTCCTTCTTCTTCTTC
Ma02_g07800	Dof	TCTCTTTTTTTTTTTTTTTTG
Ma02_g07800	MIKC_MADS	TCTCTCGCTCTCTTTTTTTTT
Ma02_g07800	BBR-BPC	ATCTCTCGCTCTCTTTTTTTT
Ma02_g07800	MIKC_MADS	TCTTCTTCTTCTTCTTCTTCT
Ma02_g07800	Dof	TCTTTTTTTTTTTTTTTTGAT
Ma02_g07800	MIKC_MADS	TCTCCTTCTTCTTCTTCTTCT
Ma02_g07800	G2-like	AGAATCTTCTA
Ma02_g07800	TCP	GGGCCCCAC
Ma02_g07800	TCP	TGCGGGCCCCACCATGATATTTTATCCACG
Ma02_g07800	MIKC_MADS	CCTTCTTCTTCTTCTTCTTCT
Ma02_g07800	G2-like	ATTAGAATCTTCTA
Ma02_g07800	TCP	CGGGCCCCAC
Ma02_g07800	G2-like	ATTAGAATCTTCTA
Ma02_g07800	Dof	CTCTTTTTTTTTTTTTTTTGA
Ma02_g07800	TCP	GCGGGCCCCAC
Ma02_g07800	HSF	ATTAGAATCTTCTATAATA
Ma02_g07800	HSF	GAATCTTCTATA
Ma02_g07800	HSF	GAATCTTCTATA
Ma02_g07800	Dof	CTTTTTTTTTTTTTTTTGATA
Ma02_g07800	Dof	TTTTTTTTTTTTTTTTGATAA
Ma02_g07800	HD-ZIP	AAAGGTAATTATGAT
Ma02_g07800	MIKC_MADS	CTTTTTTTTTTTTTTTTGATA
Ma02_g07800	TCP	GGCCCCAC
Ma02_g07800	MIKC_MADS	TTATTGCTTCTCCTTCTTCTT
Ma02_g07800	BBR-BPC	TCATCTCTCGCTCTCTTTTTT
Ma02_g07800	G2-like	AGAATCTTCTA
Ma02_g07800	MIKC_MADS	GCTTCTCCTTCTTCTTCTTCT
Ma02_g07800	BBR-BPC	CTCTCGCTCTCTTTTTTTTTT
Ma02_g07800	C2H2	TCTCCTTCTTCTTCTTCTT
Ma02_g07800	C2H2	CCTTCTTCTTCTTCTTCTT
Ma02_g07800	Dof	GCTCTCTTTTTTTTTTTTTTT
Ma02_g07800	TCP	CGGGCCCCAC
Ma02_g07800	C3H	AAAAAAAAAGGGAT
Ma02_g07800	C2H2	AGGATAAAGCCAAAGAA
Ma02_g07800	C2H2	GAAAAATAGACAAAATT
Ma02_g07800	Dof	AAGCTAAAAAAAAAAGGGATGA
Ma02_g07800	G2-like	ATTAGAATCTTCT

Ma02_g07800	AP2	GATACAAAAGGAGAATAAA
Ma02_g07800	Dof	CTCGCTCTCTTTTTTTTTTT
Ma02_g07800	C2H2	AGGATAAAGCCAAAGAAG
Ma02_g07800	C2H2	GAAAAATAGACAAAATTG
Ma02_g07800	CPP	AAAAATTTTATTA
Ma02_g07800	B3	ATAAGCATGCAACGC
Ma02_g07800	Dof	CTACCTTTATCTTTACTCTTG
Ma02_g07800	TALE	CTGTTCATCTCTCGCTCTCT
Ma02_g07800	Dof	ACAAAAGGGAGAATAAACAGT
Ma02_g07800	Dof	ACAAAAGGGAGAATAAACAGT
Ma02_g07800	BES1	CCACGCGTGCAGGTGGAGTG
Ma02_g07800	BBR-BPC	CTCGCTCTCTTTTTTTTTTT
Ma02_g07800	C2H2	TTGCTTCTCCTTCTTCTTC
Ma02_g07800	BBR-BPC	GTTCATCTCTCGCTCTCTTTT
Ma02_g07800	AP2	ACAAAATTGAAGAAAAATAA
Ma02_g07800	YABBY	TATGATAA
Ma02_g07800	Dof	CCAAGGATAAAGCCAAAGAAG
Ma02_g07800	NAC	GATCGATTGTTCTTCAAGAAA
Ma02_g07800	Dof	AATTAAGCTAAAAAAAAAGGG
Ma02_g07800	Dof	AATTAAGCTAAAAAAAAAGGG
Ma02_g07800	Dof	AAAATAGACAAAATTGAAGAA
Ma02_g07800	Dof	AAAATAGACAAAATTGAAGAA
Ma02_g07800	MIKC_MADS	GCTGTTCATCTCTCGCTCTCT
Ma02_g07800	Dof	TGATACAAAAGGGAGAATAAA
Ma02_g07800	B3	TCTACTATTCTGGTGGAAATT
Ma02_g07800	B3	TCCAACATGCAATTA
Ma02_g07800	HSF	AGAATCTTCTATAAT
Ma02_g07800	Dof	AAAAAAGGGATGAT
Ma02_g07800	Dof	AAAAAAGGGAT
Ma02_g07800	MIKC_MADS	TGTTCATCTCTCGCTCTCTTT
Ma02_g07800	bHLH	CACATGACTCCCAA
Ma02_g07800	bHLH	CACATGACTCCCAA
Ma02_g07800	Dof	AAAAAAGGGA
Ma02_g07800	C2H2	GCAGACAGTGA
Ma02_g07800	NAC	GATCGATTGTTCTTCAAGAAA
Ma02_g07800	MYB	GATTAGGTCT
Ma02_g07800	BBR-BPC	CTGTTCATCTCTCGCTCTCTT
Ma02_g07800	C2H2	TCTTCTTCTTCTTCTTCTT
Ma02_g07800	CPP	AATTTAAATC
Ma02_g07800	HSF	TTAGAATCTTC
Ma03_g07770	ERF	GTGGCGGCGGCGGTG
Ma03_g07770	ERF	GTGGCGGCGGCGGTG

Ma03_g07770	ERF	TGGCGGCGGCGGTGT
Ma03_g07770	ERF	AGCACGTGGCGGCGGCGGT
Ma03_g07770	ERF	AGCACGTGGCGGCGGCGGTGT
Ma03_g07770	GATA	ATGATGTTGATGATG
Ma03_g07770	ERF	CGTGGCGGCGGCGGT
Ma03_g07770	ERF	CACGTGGCGGCGGCG
Ma03_g07770	ERF	CACGTGGCGGCGGCG
Ma03_g07770	ERF	ACGTGGCGGCGGCGGTGTT
Ma03_g07770	ERF	CACGTGGCGGCGGCG
Ma03_g07770	ERF	GGCGGCGGCGGTGTT
Ma03_g07770	ERF	CGTGGCGGCGGCGGT
Ma03_g07770	MYB_related	TACCTTATCTATTAA
Ma03_g07770	ERF	ACAAGCACGTGGCGGCGGCGG
Ma03_g07770	ERF	GGTTCGGATCGGCGGCGGCCG
Ma03_g07770	ERF	GCACGTGGCGGCGGC
Ma03_g07770	B3	TCATTTTTTTTCACAGAAAAA
Ma03_g07770	BBR-BPC	TCCTTTTTATCTCTTTACCTC
Ma03_g07770	ERF	CAAGCACGTGGCGGCGGCGGT
Ma03_g07770	GATA	GTGGCGGCGGCGGTG
Ma03_g07770	ERF	GCACGTGGCGGCGGC
Ma03_g07770	ERF	GGCGGCGGCGGTGTT
Ma03_g07770	bHLH	CCACGTGC
Ma03_g07770	bHLH	CCACGTGC
Ma03_g07770	AP2	AAAACAAAAGGAGGAAAACA
Ma03_g07770	BES1	TCACACGTGCTGAGA
Ma03_g07770	ERF	GGATCGGCGGCGGCC
Ma03_g07770	ERF	GGATCGGCGGCGGCC
Ma03_g07770	bHLH	GCCACGTGC
Ma03_g07770	Dof	TAAAAACAAAAGGAGGAAAAC
Ma03_g07770	AP2	GAGGAAATAAATAAAAATAA
Ma03_g07770	ERF	GCATTGCATTTTCCACCGTC
Ma03_g07770	ERF	CGGCGGCGGCCGGTT
Ma03_g07770	ERF	GTGGCGGCGGCGGTG
Ma03_g07770	ERF	GCACGTGGCGGCGGCGGTGTT
Ma03_g07770	ERF	ACGTGGCGGCGGCGG
Ma03_g07770	Dof	AAAAACAAAAGGAGGAAAACA
Ma03_g07770	bHLH	GCCACGTGCC
Ma03_g07770	ERF	CGGCGGCGGTGTTCC
Ma03_g07770	bHLH	ACACGTGC
Ma03_g07770	bHLH	ACACGTGC
Ma03_g07770	ERF	GGATCGGCGGCGGCC
Ma03_g07770	MIKC_MADS	CCTTTTTATCTCTTTACCTCC

Ma03_g07770	BES1	TCACACGTGCTGAGATGGTG
Ma03_g07770	bHLH	TTTGGGCCACGTGC
Ma03_g07770	bHLH	AGCACGTGGC
Ma03_g07770	G2-like	AGGAAAACA
Ma03_g07770	BES1	CACACGTGCTG
Ma03_g07770	bHLH	GGCCACGTGC
Ma03_g07770	MIKC_MADS	CAAAAGGAGGAAAA
Ma03_g07770	bHLH	CCACGTGC
Ma03_g07770	AP2	AAAAACAAAAGGAGGAAAAC
Ma03_g07770	bHLH	ACACGTGC
Ma03_g07770	bHLH	ACACGTGC
Ma03_g07770	MYB_related	GCTACCTTATCTATT
Ma03_g07770	HD-ZIP	TAAATAATAATGGATATATAT
Ma03_g07770	HD-ZIP	ATAAAAATAATTTAACTAAAA
Ma03_g07770	ERF	TGCTGAGATGGTGGA
Ma03_g07770	WRKY	AGTTGACTTGCTTGAGATT
Ma03_g07770	bHLH	CACGTGCCACGATT
Ma03_g07770	bHLH	CACGTGCCACGATT
Ma03_g07770	AP2	AATACTAAGAAAAATAATAA
Ma03_g07770	bHLH	CACACGTGCT
Ma03_g07770	bHLH	ACACGTGC
Ma03_g07770	bHLH	CCACGTGC
Ma03_g07770	HD-ZIP	AAAATAATTTA
Ma03_g07770	GATA	ATGTTGATGATGAAT
Ma03_g07770	ERF	TCGGATCGGCGGCGGCCGG
Ma03_g07770	WRKY	CTAGTTGACTTGCT
Ma03_g07770	bHLH	ACACGTGC
Ma03_g07770	WRKY	CTAGTTGACTTG
Ma03_g07770	bHLH	CACGTGGCGGCGGCG
Ma03_g07770	ERF	CGGATCGGCGGCGGC
Ma03_g07770	BBR-BPC	AAGAAAGAATTTGAAAGAGAAAAT
Ma03_g07770	bHLH	CACGTGCCACGATTA
Ma03_g07770	bHLH	CCACGTGC
Ma03_g07770	bHLH	GCACGTGG
Ma03_g07770	bHLH	CCACGTGC
Ma03_g07770	bHLH	GCACGTGG
Ma03_g07770	MYB_related	AAATATCC
Ma03_g07770	GATA	GCAAGATCTAG
Ma03_g07770	ERF	ACGTGGCGGCGGCGGTG
Ma03_g07770	C2H2	CCATAAAAACAAAAG
Ma03_g07770	AP2	AAGAATTTGAAAGAGAAAAT
Ma03_g07770	C2H2	ACAGTCAGTGT

Ma03_g07770	bHLH	ACACGTGC
Ma03_g07770	bHLH	CCACGTGC
Ma03_g07770	WRKY	AGTTGACTTGCTT
Ma03_g07770	Dof	ACTCCTTTTTATCTCTTTACC
Ma03_g07770	ARF	CTCACAATAAAGACGGGGAAA
Ma03_g07770	Dof	TATACTTTTACATCTTAATAA
Ma03_g07770	ERF	ACGTGGCGGCGGCGG
Ma03_g07770	bHLH	GCACGTGG
Ma03_g07770	ERF	GCGGCGGCGGTGTTC
Ma03_g07770	ERF	GCGGCGGCGGTGTTC
Ma03_g07770	bHLH	TTTGGGCCACGTGCCACGATT
Ma03_g07770	HD-ZIP	ATAATAATGGA
Ma03_g07770	Dof	ACAAAAGGAGGAAAACAATAT
Ma03_g07770	Dof	ACAAAAGGAGGAAAACAATAT
Ma03_g07770	CAMTA	CCGCGTTGA
Ma03_g07770	G2-like	GGAATATTAGT
Ma03_g07770	GATA	ATACCATCATAATCT
Ma03_g07770	HSF	CGATGCTTGTCGAAG
Ma03_g07770	MIKC_MADS	ACAAAAGGAGGAAA
Ma03_g07770	bHLH	AGCACGTGG
Ma03_g07770	ERF	CGGATCGGCGGCGGC
Ma03_g07770	Dof	TAAATAGAAAATAATAAATTA
Ma03_g07770	Dof	TAAATAGAAAATAATAAATTA
Ma03_g07770	HSF	ACGTGCAAGATCTAGAATA
Ma03_g07770	bHLH	GCACGTGG
Ma03_g07770	G2-like	GACGGAATATTAGT
Ma03_g07770	Dof	CCATAAAAACAAAAGGAGGAA
Ma03_g07770	HD-ZIP	GATCATAATTATGAA
Ma03_g07770	ERF	TGCTGAGATGGTGGA
Ma03_g07770	TALE	TACCTCCTCTCCGTTTCTGT
Ma03_g07770	WRKY	CTAGTTGACTTGCTT
Ma03_g07770	G2-like	AAGAAAACG
Ma03_g07770	HD-ZIP	AAAATAATTTA
Ma03_g07770	ERF	CGTCGCCA
Ma03_g07770	GATA	GATCATAATTATGAAAAAAATATTCAG
Ma03_g07770	bHLH	AAGCACGTGG
Ma03_g07770	HD-ZIP	GATGTTGATGATGAA
Ma03_g07770	ERF	GATCGGCGGCGGCCG
Ma03_g07770	G2-like	TAGAAAACT
Ma03_g07770	bZIP	TTGGGCCACGTGCCA
Ma03_g07770	WRKY	CTAGTTGACTTGCT
Ma03_g07770	YABBY	AAATAATAAT

Ma03_g07770	YABBY	AAATAATAAT
Ma03_g07770	ERF	GGTTCGGATCGGCGGCGGC
Ma03_g07770	HD-ZIP	ATCCCAATGATTGGGGAACAG
Ma03_g07770	BBR-BPC	GAAAGAATTTGAAAGAGAAAATAT
Ma03_g07770	WRKY	CTAGTTGACTTGC
Ma03_g07770	Dof	ATAAAAGATTAAACTAATAAA
Ma03_g07770	Dof	ATAAAAGATTAAACTAATAAA
Ma03_g11520	BBR-BPC	GAGAGAGAATAAAAGAGAGAGAGA
Ma03_g11520	BBR-BPC	GAGAGAATAAAAGAGAGAGAGATA
Ma03_g11520	BBR-BPC	AATAGAGAGAGAATAAAAGAGAGA
Ma03_g11520	BBR-BPC	TAGAGAGAGAATAAAAGAGAGAGA
Ma03_g11520	AP2	GAGAATAAAAGAGAGAGAGA
Ma03_g11520	AP2	GAGAGAATAAAAGAGAGAGA
Ma03_g11520	HD-ZIP	ATAAATAATGATGAC
Ma03_g11520	AP2	GAGAGAGAATAAAAGAGAGA
Ma03_g11520	AP2	TAGAGAGAGAATAAAAGAGA
Ma03_g11520	Dof	TTAAAAAAAAAAAAGGAACTT
Ma03_g11520	MIKC_MADS	ATAAAAGTGGAAAG
Ma03_g11520	GATA	TGGATCGGATCAC
Ma03_g11520	GATA	TGGATCGGATCAC
Ma03_g11520	Dof	AAAGAGATAAAGTTATGAAAA
Ma03_g11520	ERF	CGCCGCCG
Ma03_g11520	ERF	TACGCCGCCGACGACGTGGAC
Ma03_g11520	Dof	AAAAAAAAAAAAGGAACTT
Ma03_g11520	Dof	AAATAGAAAAAGATATTAGAA
Ma03_g11520	ERF	CCGATTACGCCGCCGACGACGTGGACAG
Ma03_g11520	BBR-BPC	GAATAAAAGAGAGAGAGAGATAATTG
Ma03_g11520	ERF	TACCGATTACGCCGCCGACG
Ma03_g11520	C2H2	TGTGACAGTGA
Ma03_g11520	Dof	GAAAAAGATATTAGAAAATAT
Ma03_g11520	Dof	GAAAAAGATATTAGAAAATAT
Ma03_g11520	WRKY	CGTTGACCATAAATACCTA
Ma03_g11520	BBR-BPC	GAGAATAAAAGAGAGAGAGATAAT
Ma03_g11520	ERF	CCGATTACGCCGCCGACGACG
Ma03_g11520	bZIP	GCCGACGACGTGGAC
Ma03_g11520	AP2	CAAGGAATGAAAGAGATAAA
Ma03_g11520	HD-ZIP	TATGCAATTATTGATTGCAAT
Ma03_g11520	AP2	GATGGGGAAGAGGAAAGAAA
Ma03_g11520	Dof	CATAAATAGAAAAAGATATTA
Ma03_g11520	WRKY	ACGTTGACCA
Ma03_g11520	bHLH	TCACGTGG
Ma03_g11520	bZIP	CGACGTGGAC

Ma03_g11520	YABBY	TAATAATAAT
Ma03_g11520	ERF	TTACCGATTACGCCGCCGAC
Ma03_g11520	C3H	AAAAAAAAAGGAAC
Ma03_g11520	Dof	ATGAAAGAGATAAAGTTATGA
Ma03_g11520	WRKY	CACGTTGACCATA
Ma03_g11520	Dof	AAAAAAAAAAAGGAACTTCAC
Ma03_g11520	C2H2	CCTCGACGTTCTCCTCCAC
Ma03_g11520	BBR-BPC	GCATAATAGAGAGAGAATAAAAGA
Ma03_g11520	NAC	AGATTGAAGTACAAGCA
Ma03_g11520	NAC	AGATTGAAGTACAAG
Ma03_g11520	AP2	ATAAAAGAGAGAGAGATAAT
Ma03_g11520	NAC	AAGATTGAAGTACAAGC
Ma03_g11520	MYB	TACAGTAGATTTGGTGAGCTA
Ma03_g11520	bZIP	GCCGACGACGTGGAC
Ma03_g11520	bZIP	CATGATGTCAT
Ma03_g11520	MIKC_MADS	ATAAAAGTGGAAA
Ma03_g11520	Trihelix	ACAACACCGGCGTCG
Ma03_g11520	ERF	CAACACCGGCGTCGCAACCA
Ma03_g11520	bZIP	TCACGTGG
Ma03_g11520	NAC	ATTGAAGTACAAGCA
Ma03_g11520	bZIP	GCCGACGACGTGGAC
Ma03_g11520	ERF	AGCCGCAGCGGTGAC
Ma03_g11520	MIKC_MADS	GCATAAATAGAAA
Ma03_g11520	C2H2	CACATGGAGAAGAAG
Ma03_g11520	ERF	ACGCCGCCGA
Ma03_g11520	CPP	CAAAATTTAAGAA
Ma03_g11520	TCP	TGGACCAC
Ma03_g11520	MIKC_MADS	GGATAAAAGTGGAAA
Ma03_g11520	bZIP	ATGCATGATGTCATT
Ma03_g11520	MYB	AGATTTGGTGAGC
Ma03_g11520	WRKY	CACGTTGACCAT
Ma03_g11520	BBR-BPC	GACAGGAGTAGAAATATAAGAAGA
Ma03_g11520	C3H	AGAGATAAAGTTAT
Ma03_g11520	C2H2	CCTCGTCCTTCGTCCCCTC
Ma03_g11520	ERF	ATTACGCCGCCGACGACGTGG
Ma03_g11520	ERF	ATTACGCCGCCGACGACGTGG
Ma03_g11520	ERF	ATTACGCCGCCGACG
Ma03_g11520	Dof	GATAAAGTTAT
Ma03_g11520	bZIP	GATTCACGTGGG
Ma03_g11520	bZIP	GATTCACGTGGG
Ma03_g11520	bZIP	GATTCACGTGGG
Ma03_g11520	bZIP	CGACGACGTGGA

Ma03_g11520	bZIP	CGACGACGTGGA
Ma03_g11520	bZIP	CGACGACGTGGA
Ma03_g11520	AP2	AAAAAAAAAAAGGAACTTCA
Ma03_g11520	bZIP	CCATCTGATGTGTCA
Ma03_g11520	TALE	TCCTCGTCCTTCGTCCCCTC
Ma03_g11520	HD-ZIP	GCAGCAATCATAGATGAATAT
Ma03_g11520	C2H2	CGTCCTTCGTCCCCTCCTT
Ma03_g11520	MYB	TAGATTTGGTGAGCT
Ma03_g11520	C2H2	CCTTCGTCCCCTCCTTTAC
Ma03_g11520	BBR-BPC	ATAATAGAGAGAGAATAAAAGAGA
Ma03_g11520	ERF	ACACCGGCGTCGCAA
Ma03_g11520	C2H2	TCCCCTCCTTTACTTCCAT
Ma03_g11520	ERF	CGATTACGCCGCCGACGACG
Ma03_g11520	ERF	ATAGGAGGAGGAAATGGTGAT
Ma03_g11520	G2-like	GGAATCTA
Ma03_g11520	C3H	GATAAAGTTAT
Ma03_g11520	bZIP	CATGATGTCATTTCT
Ma03_g11520	BBR-BPC	AAGAGATAAATTAATAGAAAAATA
Ma03_g11520	NAC	ATAAGATTGAAGTACAAGCAT
Ma03_g11520	TCP	AGTGGACCAC
Ma03_g11520	HD-ZIP	GCAATAATTCT
Ma03_g11520	C2H2	CGTTTCCCTCCTCCACATA
Ma03_g11520	C3H	ATAGAAAAAGATAT
Ma03_g11520	BBR-BPC	CAAGGAATGAAAGAGATAAAGTTA
Ma03_g11520	MYB_related	AAGATATTA
Ma03_g11520	Dof	GATAAAGTTATGAA
Ma03_g11520	ERF	ACACCGGCGTCGCAA
Ma03_g11520	Dof	AAAAAAGGAACTTC
Ma03_g11520	YABBY	GAATAATAAT
Ma03_g11520	bZIP	ATGCATGATGTCATT
Ma03_g11520	HD-ZIP	AGCAATCATAG
Ma03_g11520	ERF	CCCAGACAACACCGGCGTCG
Ma03_g11520	MIKC_MADS	GCATAAATAGAAAA
Ma03_g11520	bZIP	CGACGTGG
Ma03_g11520	WRKY	CACGTTGACCATAA
Ma03_g11520	bZIP	CGCCGACGACGTGGACAG
Ma03_g11520	HSF	GAACGCTCGAGA
Ma03_g11520	C3H	AAAAAAGGAAC
Ma03_g11520	MYB_related	AAGATATTA
Ma03_g11520	bZIP	TCACGTGGGA
Ma03_g11520	BBR-BPC	GAAAGAGATAAAGTTATGAAAAAA
Ma03_g11520	bHLH	CATGATTCACGTGG

Ma03_g11520	ERF	ATTACGCCGCCGACG
Ma03_g11520	bHLH	CATGATTCACGTGGGAACTGA
Ma03_g11520	NAC	ATAAGATTGAAGTACAAGCAT
Ma03_g11520	WRKY	CACGTTGACCATAA
Ma03_g11520	WOX	GCAATCAA
Ma03_g11520	WOX	GCAATCAA
Ma03_g11520	WOX	GCAATCAA
Ma03_g11520	WOX	GCAATCAA
Ma03_g11520	ERF	TTTTACCGATTACGCCGCCG
Ma03_g11520	HSF	GGAACGCTCGAGAGT
Ma03_g11520	Trihelix	TATTTTACCG
Ma03_g11520	Dof	TAAATAGAAAAAGATATTA
Ma03_g11520	HD-ZIP	TGCAATCAATT
Ma03_g11520	bZIP	GCCGACGACGTGGAC
Ma03_g11520	G2-like	AAGAAAACG
Ma03_g11520	Dof	GAAAGAGATAAAGTTATGA
Ma03_g11520	bZIP	GCCGACGACGTGGAC
Ma03_g11520	GATA	TAACCATCACCTTAA
Ma03_g11520	HD-ZIP	GCAATAATTCT
Ma03_g11520	MYB	GATCTAACCATCACC
Ma03_g11520	G2-like	GGAATCTA
Ma03_g11520	Trihelix	TATTTTACCGATTA
Ma03_g11520	bZIP	GTGCAAGCTGGT
Ma03_g11520	BBR-BPC	ATAAAAGAGAGAGAGATAATTGAG
Ma03_g11520	NAC	AGATTGAAGTACAAG
Ma03_g11520	MIKC_MADS	GGGGAAGAGGAAAG
Ma03_g11520	HD-ZIP	AAAGCAATAATTCTAATTTGG
Ma03_g11520	bZIP	TGACGTGT
Ma03_g11520	HD-ZIP	CAATAATT
Ma03_g11520	HSF	CGAGTAGCTTC
Ma03_g11520	C2H2	CACATATCACCTTTACCC
Ma03_g11520	MYB	AGTAGATTTGGTGAG
Ma03_g11520	Dof	AAAAAAGGAAC
Ma03_g11520	BBR-BPC	AAAAGAGAGAGAGATAATTGAGTC
Ma03_g11520	YABBY	TAATAATAAA
Ma03_g11520	AP2	AGGAATGAAAGAGATAAAGT
Ma03_g11520	bZIP	CCATCTGATGTGTCA
Ma03_g15580	BBR-BPC	AAGAGAAAGAGAAAAAGAAACATA
Ma03_g15580	AP2	AAGAGAAAGAGAAAAAGAAA
Ma03_g15580	AP2	GAGAAAGAGAAAAAGAAACA
Ma03_g15580	AP2	GAAGACAAGAGAAAGAGAAA
Ma03_g15580	ARF	TTTGTAGTTTTTGTTGGAAAA

Ma03_g15580	Dof	AAAGAGAAAAAGAAACATAAA
Ma03_g15580	B3	TTTGTAGTTTTTGTTGGAAAA
Ma03_g15580	BBR-BPC	GAGAAAGAGAAAAAGAAACATAAA
Ma03_g15580	AP2	AGACAAGAGAAAGAGAAAAA
Ma03_g15580	Dof	GAGAAAGAGAAAAAGAAACAT
Ma03_g15580	BBR-BPC	ACAAGAGAAAGAGAAAAAGAAACA
Ma03_g15580	B3	TTGTAGTTTTTGTTGGAAAAT
Ma03_g15580	Dof	TTTTCTTTTTTCATCTTTGCA
Ma03_g15580	BBR-BPC	GTGAAGACAAGAGAAAGAGAAAAA
Ma03_g15580	AP2	AAAAATAAAAATAAAAATAA
Ma03_g15580	NAC	CTTGAGCAATAAGCA
Ma03_g15580	AP2	ACAAGAGAAAGAGAAAAAGA
Ma03_g15580	AP2	TCAAAAAAGGAAGGGAGAAG
Ma03_g15580	Dof	AAAAAATAAAAATAAAAATAA
Ma03_g15580	Dof	AAAAAATAAAAATAAAAATAA
Ma03_g15580	bZIP	GATGACATCAC
Ma03_g15580	BBR-BPC	CTGAGGTGGAGAGGGGGAGGAGAA
Ma03_g15580	ERF	AGCCGAGGAGGTGGA
Ma03_g15580	MYB	CAATAAGATAAGG
Ma03_g15580	HD-ZIP	CCAATAATTCA
Ma03_g15580	MIKC_MADS	TCAAAAAAGGAAGG
Ma03_g15580	bZIP	GGGGATGACATCACA
Ma03_g15580	AP2	CAGGAATAGGATAAAAGAGG
Ma03_g15580	BBR-BPC	GAAAGAGAAAAAGAAACATAAATT
Ma03_g15580	G2-like	AAAGGAATATATCA
Ma03_g15580	NAC	CTTGAGCAATAAGCA
Ma03_g15580	HD-ZIP	CCAATAATTCA
Ma03_g15580	Dof	GAAAAAATAAAAATAAAAATA
Ma03_g15580	Dof	GAAAAAATAAAAATAAAAATA
Ma03_g15580	BBR-BPC	GAAGACAAGAGAAAGAGAAAAAGA
Ma03_g15580	Dof	GAGAAAGAGAAAAAGAAACAT
Ma03_g15580	Dof	GAGAAAGAGAAAAAGAAACAT
Ma03_g15580	Dof	TAAAAAGGAATATA
Ma03_g15580	Dof	AAAAAAGGAAGGGAGAAGACC
Ma03_g15580	Dof	AAAAAAGGAAGGGAGAAGACC
Ma03_g15580	Dof	TTTCTTTTTTCATCTTTGCAT
Ma03_g15580	BBR-BPC	GAGGTGGAGAGGGGGAGGAGAATA
Ma03_g15580	ERF	TGAGGATGACGGAGGTGGG
Ma03_g15580	AP2	GGGGAGGAGAATAAGATAGG
Ma03_g15580	NAC	CTTGAGCAATAAGCAT
Ma03_g15580	Dof	AGAAAGGTCAAAAAAGGAAGG
Ma03_g15580	Dof	AGAAAGGTCAAAAAAGGAAGG

Ma03_g15580	C2H2	TGATCTCATCTTCCTTCTC
Ma03_g15580	YABBY	TATGATTA
Ma03_g15580	ERF	TGTGAGGATGACGGAGGTGGG
Ma03_g15580	C2H2	CTTCCTCTACCTTCTTCAA
Ma03_g15580	G2-like	AAAGGAATATATC
Ma03_g15580	MYB	TTCACCAAACTCCAT
Ma03_g15580	Dof	GAAAAAGAAACATAAATTGCA
Ma03_g15580	Dof	GAAAAAGAAACATAAATTGCA
Ma03_g15580	AP2	AAAGAGAAAAAGAAACATAA
Ma03_g15580	G2-like	AAAGGAATATA
Ma03_g15580	MYB	CAGGAATAGGATAAAAGAGGT
Ma03_g15580	EIL	TCATGTACCTA
Ma03_g15580	ERF	GCGGAGACCCGGTCACCGAC
Ma03_g15580	HD-ZIP	ATAATAATTCA
Ma03_g15580	MYB	CATTAACTAGATAAGCTTTGT
Ma03_g15580	ERF	GATGACGGAGGTGG
Ma03_g15580	ERF	GATGACGGAGGTGG
Ma03_g15580	bZIP	GGGGATGACATCACA
Ma03_g15580	HD-ZIP	TAATAATT
Ma03_g15580	NAC	CTTGAGCAATAAGCAT
Ma03_g15580	NAC	CTTGAGCAATAAGCAT
Ma03_g15580	BBR-BPC	TTGTGAAGACAAGAGAAAGAGAAA
Ma03_g15580	BBR-BPC	AAGAAAGGTCAAAAAAGGAAGGGA
Ma03_g15580	C2H2	TAGATGTTTGTCATCGTGCG
Ma03_g15580	NAC	TTCTTGAGCAATAAGCA
Ma03_g15580	bHLH	TCACATGG
Ma03_g15580	ERF	GATGGCAGAAGAAGCGGAGAC
Ma03_g15580	GATA	TTATCTTCATAATAA
Ma03_g15580	Dof	GAGAAAGAAAGGTCAAAAAAG
Ma03_g15580	Dof	GAGAAAGAAAGGTCAAAAAAG
Ma03_g15580	G2-like	GGAATCTA
Ma03_g15580	G2-like	AAAGGAATATATCA
Ma03_g15580	BBR-BPC	GAAAGGTCAAAAAAGGAAGGGAGA
Ma03_g15580	MIKC_MADS	ATTCATGAAATAGAAAAAT
Ma03_g15580	AP2	TGGACAGTGAAAAAATAAAA
Ma03_g15580	C2H2	TGTTTGTCATCGTGCG
Ma03_g15580	ERF	GGGCCGCCGA
Ma03_g15580	WOX	TACCAATCAG
Ma03_g15580	ERF	GATGACGGAGGTGGG
Ma03_g15580	GATA	AGGATGACGGAGGTG
Ma03_g15580	MIKC_MADS	AGAGAAAAAGAAAC
Ma03_g15580	HD-ZIP	ATAATAATTCA

Ma03_g15580	MIKC_MADS	AGAGAAAGAGAAAA
Ma03_g15580	ERF	GAGGATGACGGAGGT
Ma03_g15580	WRKY	AAAGGTCAAAAAA
Ma03_g15580	MIKC_MADS	ATTTTTCTTTTTTCATCTT
Ma03_g15580	MIKC_MADS	ATTTTTCTTTTTTCATCTT
Ma03_g15580	Dof	GAAAGGTCAAAAAAGGAAGGG
Ma03_g15580	AP2	GTGAAAAAATAAAAATAAAA
Ma03_g15580	ERF	GAAGGTGCCGATGGC
Ma03_g15580	ERF	TTGTGAGGATGACGGAGGTGG
Ma03_g15580	NAC	TTCTTGAGCAATAAG
Ma03_g15580	Dof	GAAAGAGAAAAAGAAACAT
Ma03_g15580	MYB	TTTGTAGTTTTTGTTGGAAAA
Ma03_g15580	MIKC_MADS	CATGAAATAGAAAA
Ma03_g15580	MIKC_MADS	ATTCCCTACTGGGTA
Ma03_g15580	GATA	TTATTATCTTCATAA
Ma03_g15580	GATA	ATTTTTAATGATCTTGATCTTGATCCA
Ma03_g15580	RAV	TATGTTGT
Ma03_g15580	MIKC_MADS	TACTAAAAATGATAA
Ma03_g15580	BBR-BPC	AGACAAGAGAAAGAGAAAAAGAAA
Ma03_g15580	B3	ATAAGGGTTTTGGCGGATATC
Ma03_g15580	MIKC_MADS	TCCCTTCTATTTGTAGTTT
Ma03_g15580	MIKC_MADS	TCCCTTCTATTTGTAGTTT
Ma03_g15580	WRKY	AAAGGTCAAAAAA
Ma03_g15580	NAC	CTTCTTGAGCAATAAGCAT
Ma03_g15580	Dof	TAAATTTTTCTTTTTTCATCT
Ma03_g15580	C3H	TAAAAAGGAAT
Ma03_g15580	YABBY	CTATCATGAT
Ma03_g15580	MYB	GTTTTTGTTGG
Ma03_g15580	C2H2	AGAGAAAGAGAAAAAA
Ma03_g15580	MIKC_MADS	ATAAAAGAGGTAAT
Ma03_g15580	G2-like	GGAATCTA
Ma03_g15580	C3H	AGAGAAAAAGAAAC
Ma03_g15580	GATA	ATGACGGAGGTGGGC
Ma03_g15580	Dof	TTTATTTTTATGATTATTTAT
Ma03_g15580	C2H2	AAGAAAGGTCAAAAA
Ma03_g15580	Dof	ACCTCTTTTCTATTCTGTGTT
Ma03_g15580	MYB	TAACTAGATAAGC
Ma03_g15580	HD-ZIP	CAATAATT
Ma03_g15580	Dof	GAAATAGAAAAATGTGTATAG
Ma03_g15580	Dof	GAAATAGAAAAATGTGTATAG
Ma03_g15580	ERF	GATGACGGAGGTGGG
Ma03_g15580	NAC	CTTGAGCAATAAG

Ma03_g15580	C2H2	TCTCATCTTCCTTCTCAGG
Ma03_g15580	LBD	GCAGAAGAAGCGGAG
Ma03_g15580	Dof	ATAAAAGAGGTAATAAATAAA
Ma03_g15580	Dof	ATAAAAGAGGTAATAAATAAA
Ma03_g15580	C2H2	AAAGAAAGGTCAAAAAAG
Ma03_g15580	G2-like	GGCATCTA
Ma03_g15580	Dof	TTTATTCTTATTTATTTTTAT
Ma03_g15580	AP2	AAAAAGGAAGGGAGAAGACC
Ma03_g15580	BBR-BPC	AAGAGAAAGAGAAAAAGAAACATA
Ma03_g15580	AP2	AAGAGAAAGAGAAAAAGAAA
Ma03_g15580	AP2	GAGAAAGAGAAAAAGAAACA
Ma03_g15580	AP2	GAAGACAAGAGAAAGAGAAA
Ma03_g15580	ARF	TTTGTAGTTTTTGTTGGAAAA
Ma03_g15580	Dof	AAAGAGAAAAAGAAACATAAA
Ma03_g15580	B3	TTTGTAGTTTTTGTTGGAAAA
Ma03_g15580	BBR-BPC	GAGAAAGAGAAAAAGAAACATAAA
Ma03_g15580	AP2	AGACAAGAGAAAGAGAAAAA
Ma03_g15580	Dof	GAGAAAGAGAAAAAGAAACAT
Ma03_g15580	BBR-BPC	ACAAGAGAAAGAGAAAAAGAAACA
Ma03_g15580	B3	TTGTAGTTTTTGTTGGAAAAT
Ma03_g15580	Dof	TTTTCTTTTTTCATCTTTGCA
Ma03_g15580	BBR-BPC	GTGAAGACAAGAGAAAGAGAAAAA
Ma03_g15580	AP2	AAAAATAAAAATAAAAATAA
Ma03_g15580	NAC	CTTGAGCAATAAGCA
Ma03_g15580	AP2	ACAAGAGAAAGAGAAAAAGA
Ma03_g15580	AP2	TCAAAAAAGGAAGGGAGAAG
Ma03_g15580	Dof	AAAAAATAAAAATAAAAATAA
Ma03_g15580	Dof	AAAAAATAAAAATAAAAATAA
Ma03_g15580	bZIP	GATGACATCAC
Ma03_g15580	BBR-BPC	CTGAGGTGGAGAGGGGGAGGAGAA
Ma03_g15580	ERF	AGCCGAGGAGGTGGA
Ma03_g15580	MYB	CAATAAGATAAGG
Ma03_g15580	HD-ZIP	CCAATAATTCA
Ma03_g15580	MIKC_MADS	TCAAAAAAGGAAGG
Ma03_g15580	bZIP	GGGGATGACATCACA
Ma03_g15580	AP2	CAGGAATAGGATAAAAGAGG
Ma03_g15580	BBR-BPC	GAAAGAGAAAAAGAAACATAAATT
Ma03_g15580	G2-like	AAAGGAATATATCA
Ma03_g15580	NAC	CTTGAGCAATAAGCA
Ma03_g15580	HD-ZIP	CCAATAATTCA
Ma03_g15580	Dof	GAAAAAATAAAAATAAAAATA
Ma03_g15580	Dof	GAAAAAATAAAAATAAAAATA

Ma03_g15580	BBR-BPC	GAAGACAAGAGAAAGAGAAAAAGA
Ma03_g15580	Dof	GAGAAAGAGAAAAAGAAACAT
Ma03_g15580	Dof	GAGAAAGAGAAAAAGAAACAT
Ma03_g15580	Dof	TAAAAAGGAATATA
Ma03_g15580	Dof	AAAAAAGGAAGGGAGAAGACC
Ma03_g15580	Dof	AAAAAAGGAAGGGAGAAGACC
Ma03_g15580	Dof	TTTCTTTTTCATCTTTGCAT
Ma03_g15580	BBR-BPC	GAGGTGGAGAGGGGGAGGAGAATA
Ma03_g15580	ERF	TGAGGATGACGGAGGTGGG
Ma03_g15580	AP2	GGGGAGGAGAATAAGATAGG
Ma03_g15580	NAC	CTTGAGCAATAAGCAT
Ma03_g15580	Dof	AGAAAGGTCAAAAAAGGAAGG
Ma03_g15580	Dof	AGAAAGGTCAAAAAAGGAAGG
Ma03_g15580	C2H2	TGATCTCATCTTCCTTCTC
Ma03_g15580	YABBY	TATGATTA
Ma03_g15580	ERF	TGTGAGGATGACGGAGGTGGG
Ma03_g15580	C2H2	CTTCCTCTACCTTCTTCAA
Ma03_g15580	G2-like	AAAGGAATATATC
Ma03_g15580	MYB	TTCACCAAACTCCAT
Ma03_g15580	Dof	GAAAAAGAAACATAAATTGCA
Ma03_g15580	Dof	GAAAAAGAAACATAAATTGCA
Ma03_g15580	AP2	AAAGAGAAAAAGAAACATAA
Ma03_g15580	G2-like	AAAGGAATATA
Ma03_g15580	MYB	CAGGAATAGGATAAAAGAGGT
Ma03_g15580	EIL	TCATGTACCTA
Ma03_g15580	ERF	GCGGAGACCCGGTCACCGAC
Ma03_g15580	HD-ZIP	ATAATAATTCA
Ma03_g15580	MYB	CATTAACTAGATAAGCTTTGT
Ma03_g15580	ERF	GATGACGGAGGTGG
Ma03_g15580	ERF	GATGACGGAGGTGG
Ma03_g15580	bZIP	GGGGATGACATCACA
Ma03_g15580	HD-ZIP	TAATAATT
Ma03_g15580	NAC	CTTGAGCAATAAGCAT
Ma03_g15580	NAC	CTTGAGCAATAAGCAT
Ma03_g15580	BBR-BPC	TTGTGAAGACAAGAGAAAGAGAAA
Ma03_g15580	BBR-BPC	AAGAAAGGTCAAAAAAGGAAGGGA
Ma03_g15580	C2H2	TAGATGTTTGTCATCGTGCG
Ma03_g15580	NAC	TTCTTGAGCAATAAGCA
Ma03_g15580	bHLH	TCACATGG
Ma03_g15580	ERF	GATGGCAGAAGAAGCGGAGAC
Ma03_g15580	GATA	TTATCTTCATAATAA
Ma03_g15580	Dof	GAGAAAGAAAGGTCAAAAAAG

Ma03_g15580	Dof	GAGAAAGAAAGGTCAAAAAAG
Ma03_g15580	G2-like	GGAATCTA
Ma03_g15580	G2-like	AAAGGAATATATCA
Ma03_g15580	BBR-BPC	GAAAGGTCAAAAAAGGAAGGGAGA
Ma03_g15580	MIKC_MADS	ATTCATGAAATAGAAAAAT
Ma03_g15580	AP2	TGGACAGTGAAAAAATAAAA
Ma03_g15580	C2H2	TGTTTGTCATCGTGCG
Ma03_g15580	ERF	GGGCCGCCGA
Ma03_g15580	WOX	TACCAATCAG
Ma03_g15580	ERF	GATGACGGAGGTGGG
Ma03_g15580	GATA	AGGATGACGGAGGTG
Ma03_g15580	MIKC_MADS	AGAGAAAAAGAAAC
Ma03_g15580	HD-ZIP	ATAATAATTCA
Ma03_g15580	MIKC_MADS	AGAGAAAGAGAAAA
Ma03_g15580	ERF	GAGGATGACGGAGGT
Ma03_g15580	WRKY	AAAGGTCAAAAAA
Ma03_g15580	MIKC_MADS	ATTTTTCTTTTTTCATCTT
Ma03_g15580	MIKC_MADS	ATTTTTCTTTTTTCATCTT
Ma03_g15580	Dof	GAAAGGTCAAAAAAGGAAGGG
Ma03_g15580	AP2	GTGAAAAAATAAAAATAAAA
Ma03_g15580	ERF	GAAGGTGCCGATGGC
Ma03_g15580	ERF	TTGTGAGGATGACGGAGGTGG
Ma03_g15580	NAC	TTCTTGAGCAATAAG
Ma03_g15580	Dof	GAAAGAGAAAAAGAAACAT
Ma03_g15580	MYB	TTTGTAGTTTTTGTTGGAAAA
Ma03_g15580	MIKC_MADS	CATGAAATAGAAAA
Ma03_g15580	MIKC_MADS	ATTCCCTACTGGGTA
Ma03_g15580	GATA	TTATTATCTTCATAA
Ma03_g15580	GATA	ATTTTTAATGATCTTGATCTTGATCCA
Ma03_g15580	RAV	TATGTTGT
Ma03_g15580	MIKC_MADS	TACTAAAAATGATAA
Ma03_g15580	BBR-BPC	AGACAAGAGAAAGAGAAAAAGAAA
Ma03_g15580	B3	ATAAGGGTTTTGGCGGATATC
Ma03_g15580	MIKC_MADS	TCCCTTCTATTTGTAGTTT
Ma03_g15580	MIKC_MADS	TCCCTTCTATTTGTAGTTT
Ma03_g15580	WRKY	AAAGGTCAAAAAA
Ma03_g15580	NAC	CTTCTTGAGCAATAAGCAT
Ma03_g15580	Dof	TAAATTTTTCTTTTTTCATCT
Ma03_g15580	C3H	TAAAAAGGAAT
Ma03_g15580	YABBY	CTATCATGAT
Ma03_g15580	MYB	GTTTTTGTTGG
Ma03_g15580	C2H2	AGAGAAAGAGAAAAA

Ma03_g15580	MIKC_MADS	ATAAAAGAGGTAAT
Ma03_g15580	G2-like	GGAATCTA
Ma03_g15580	C3H	AGAGAAAAAGAAAC
Ma03_g15580	GATA	ATGACGGAGGTGGGC
Ma03_g15580	Dof	TTTATTTTTATGATTATTTAT
Ma03_g15580	C2H2	AAGAAAGGTCAAAAA
Ma03_g15580	Dof	ACCTCTTTTCTATTCTGTGTT
Ma03_g15580	MYB	TAACTAGATAAGC
Ma03_g15580	HD-ZIP	CAATAATT
Ma03_g15580	Dof	GAAATAGAAAAATGTGTATAG
Ma03_g15580	Dof	GAAATAGAAAAATGTGTATAG
Ma03_g15580	ERF	GATGACGGAGGTGGG
Ma03_g15580	NAC	CTTGAGCAATAAG
Ma03_g15580	C2H2	TCTCATCTTCCTTCTCAGG
Ma03_g15580	LBD	GCAGAAGAAGCGGAG
Ma03_g15580	Dof	ATAAAAGAGGTAATAAATAAA
Ma03_g15580	Dof	ATAAAAGAGGTAATAAATAAA
Ma03_g15580	C2H2	AAAGAAAGGTCAAAAAAG
Ma03_g15580	G2-like	GGCATCTA
Ma03_g15580	Dof	TTTATTCTTATTTATTTTTAT
Ma03_g15580	AP2	AAAAAGGAAGGGAGAAGACC
Ma06_g26840	HD-ZIP	TGACCAATAATTGATCATAAT
Ma06_g26840	bZIP	GGTGACGTCAGCGCA
Ma06_g26840	MIKC_MADS	TGTTTATTTCTTTTTATTTTC
Ma06_g26840	HD-ZIP	CCAATAATTGA
Ma06_g26840	HD-ZIP	CCAATAATTGA
Ma06_g26840	Dof	TTTTTTTTTTAATTTTATTTA
Ma06_g26840	bZIP	GGTGACGTCAG
Ma06_g26840	TCP	AGTGGGTCCCAACAAAACACCTATTTAATT
Ma06_g26840	Dof	GAAAAAGGGAA
Ma06_g26840	bZIP	GCGGGTGACGTCAGC
Ma06_g26840	WOX	AATCAATCAA
Ma06_g26840	C2H2	CGTCGTTCTCCTCTGCCTC
Ma06_g26840	TCP	GTGGGTCCCAA
Ma06_g26840	Dof	ATTTCTTTTTATTTTCATCTG
Ma06_g26840	C2H2	TAAATACCACCTCCGCTG
Ma06_g26840	bZIP	GCGGGTGACGTCAGC
Ma06_g26840	G2-like	AAGAAAACA
Ma06_g26840	C2H2	TAATAAAGACAATAA
Ma06_g26840	C2H2	GTGATATTTGTCATTTTTTA
Ma06_g26840	C3H	GAAAAAGGGAA
Ma06_g26840	TCP	GAGTGGGTCCCAA

Ma06_g26840	C2H2	TATTTGTCATTTTTTA
Ma06_g26840	HD-ZIP	AATAATAATAATTAT
Ma06_g26840	YABBY	TAATAATAAT
Ma06_g26840	YABBY	TAATAATAAT
Ma06_g26840	YABBY	TAATAATAAT
Ma06_g26840	YABBY	TAATAATAAT
Ma06_g26840	YABBY	TAATAATAAT
Ma06_g26840	YABBY	TAATAATAAT
Ma06_g26840	YABBY	TAATAATAAT
Ma06_g26840	YABBY	TAATAATAAT
Ma06_g26840	YABBY	TAATAATAAT
Ma06_g26840	YABBY	TAATAATAAT
Ma06_g26840	YABBY	TAATAATAAT
Ma06_g26840	YABBY	TAATAATAAT
Ma06_g26840	C3H	CAGGAAAAAGGGAA
Ma06_g26840	TALE	GGTGACGTCA
Ma06_g26840	Dof	ATTTTTTTTTTAATTTTATTT
Ma06_g26840	HD-ZIP	AATAATAATTATTAT
Ma06_g26840	bHLH	GGCCATGTGA
Ma06_g26840	TALE	TCGTTCTCCTCTGCCTCGGC
Ma06_g26840	TCP	GGGACCAT
Ma06_g26840	WOX	TCAATCAA
Ma06_g26840	WOX	TCAATCAA
Ma06_g26840	YABBY	CTATAATAAT
Ma06_g26840	TCP	CAGTGGACCCAAC
Ma06_g26840	ERF	ACCACCTCCGCTGCAATCGAT
Ma06_g26840	Dof	TTTTTTTTTAATTTTATTTAA
Ma06_g26840	HD-ZIP	TCATTTAATAT
Ma06_g26840	BBR-BPC	TAAGGAGGGAGATGGATAAAGATT
Ma06_g26840	ERF	TACCACCTCCGCTGCAATCG
Ma06_g26840	TCP	GGGACCAG
Ma06_g26840	TCP	GGGACCAG
Ma06_g26840	HD-ZIP	AAATATAATGATAAA
Ma06_g26840	ARF	ATTTTGATTTCAACGTGAAAT
Ma06_g26840	TCP	ATTGGGACCAGGA
Ma06_g26840	TCP	ATTGGGACCAGGA
Ma06_g26840	HD-ZIP	TAATAATT
Ma06_g26840	HD-ZIP	TAATAATT
Ma06_g26840	HD-ZIP	ATAATAATTAT
Ma06_g26840	HD-ZIP	AATCAATCAA
Ma06_g26840	Dof	TTACAGGAAAAAGGGAACC
Ma06_g26840	HD-ZIP	ATAATAATTAT

Ma06_g26840	MYB_related	GTACTTATCCACATC
Ma06_g26840	Dof	ATAATAATAATAAAGACAATA
Ma06_g26840	bZIP	GCGGGTGACGTCAGC
Ma06_g26840	C2H2	CAATCAAGACAATAA
Ma06_g26840	TCP	GGGACCAG
Ma06_g26840	TCP	GGGACCAG
Ma06_g26840	MYB	GGGACGTTACA
Ma06_g26840	MYB	GGGACGTTACA
Ma06_g26840	SBP	ATCATTGTACCCAATT
Ma06_g26840	SRS	AGCCTAGGGG
Ma06_g26840	HD-ZIP	TCATTTAATAT
Ma06_g26840	GATA	GGGGATAATAATAATAATAATAATTAT
Ma06_g26840	MIKC_MADS	ATTCCCAATTGGGAC
Ma06_g26840	ERF	ATACCACCTCCGCTGCAATCG
Ma06_g26840	ERF	ATACCACCTCCGCTGCAATCG
Ma06_g26840	B3	ATATTTATTTTGTGTGGAAAA
Ma06_g26840	MYB_related	GTGTACTTATCCACA
Ma06_g26840	ERF	CCACCTCCGCTGCAATCGATC
Ma06_g26840	ERF	CCACCTCCGCTGCAATCGATC
Ma06_g26840	Dof	TCATTTTTTTTTTAATTTTAT
Ma06_g26840	YABBY	TATGATAA
Ma06_g26840	GATA	ATTGATCATAATAATAATAAAGACAAT
Ma06_g26840	Dof	ATCATTTTTTTTTTAATTTTA
Ma06_g26840	B3	ATTTTGATTTCAACGTGAAAT
Ma06_g26840	SBP	TGGTACGGC
Ma06_g26840	GATA	GATCATAATAATAATAAAGACAATAAA
Ma06_g26840	Dof	TACAGGAAAAAGGGAACCGAA
Ma06_g26840	BBR-BPC	ACTAAGGAGGGAGATGGATAAAGA
Ma06_g26840	TALE	CGGTCGTCGTTCTCCTCTGC
Ma06_g26840	HD-ZIP	ATAATAATTTG
Ma06_g26840	MIKC_MADS	CTATTCCCAATTGGG
Ma06_g26840	SRS	GCCGTAGGGC
Ma06_g26840	AP2	AATAATAAAGACAATAAAGA
Ma06_g26840	SBP	AGTGTACTTAT
Ma06_g26840	C2H2	CATTTAACACCTTTTTTA
Ma06_g26840	MYB	TCTACCAACTGAACC
Ma06_g26840	MYB_related	AATATAATCCATATG
Ma06_g26840	Dof	GAAAAAGGGAACCG
Ma06_g26840	C2H2	CAGATGACACATAAATCA
Ma06_g26840	C2H2	CGTTCTCCTCTGCCTCGGC
Ma06_g26840	Dof	ATAATAATAAAGACAATAAAG
Ma06_g26840	MYB_related	TAATATCT

Ma06_g26840	SBP	TGGTACGGCG
Ma06_g26840	C2H2	ATAATAAAGACAATAAA
Ma06_g26840	Dof	TAATGATAAAAAGAACACA
Ma06_g26840	HD-ZIP	ATAATAATTTG
Ma06_g26840	SBP	TGGTACGGCG
Ma06_g26840	AP2	TATGAAAGAGATGAAAAACA
Ma06_g26840	MYB_related	ATAATATAATCCATA
Ma06_g26840	GATA	ATTAATAATAATAATAATAATAATAAT
Ma06_g26840	GATA	TTCAGATCTAT
Ma06_g26840	HD-ZIP	ATAATAATAATTATTATTAGT
Ma06_g26840	HD-ZIP	CAATAATT
Ma06_g26840	TCP	ATGGGACCAT
Ma06_g26840	YABBY	TAATAATAAA
Ma06_g26840	GATA	TCATTATTACCCTCA
Ma06_g26840	TCP	TATGGGACCATAA
Ma06_g26850	HD-ZIP	TGACCAATAATTGATCATTAT
Ma06_g26850	NAC	CTTGTTCGACACGCA
Ma06_g26850	NAC	TGCTTGTTCGACACGCA
Ma06_g26850	MIKC_MADS	TGTTTATTTCTTTTTATTTTC
Ma06_g26850	HD-ZIP	CCAATAATTGA
Ma06_g26850	HD-ZIP	CCAATAATTGA
Ma06_g26850	bZIP	GGTGACGTCAG
Ma06_g26850	WOX	AATCAATCAA
Ma06_g26850	C2H2	CGTCGTTCTCCTCTGCCTC
Ma06_g26850	bZIP	GGTGACGTCAGTGCA
Ma06_g26850	bZIP	GCGGGTGACGTCAGT
Ma06_g26850	Dof	ATTTCTTTTTATTTTCATCTG
Ma06_g26850	C2H2	TAAATACCACCTCCGCTG
Ma06_g26850	BBR-BPC	TAAAGAGGGAGATGGATAAAGATT
Ma06_g26850	YABBY	CAATAATAAT
Ma06_g26850	NAC	GGTAGAGTGTCATCCAAGCAT
Ma06_g26850	G2-like	AAGAAAACA
Ma06_g26850	NAC	GGTAGAGTGTCATCCAAGCAT
Ma06_g26850	bZIP	GCGGGTGACGTCAGT
Ma06_g26850	C2H2	TAATAAAGACAATAA
Ma06_g26850	C2H2	GTGATATTTGTCATTTTTTA
Ma06_g26850	C2H2	TATTTGTCATTTTTTA
Ma06_g26850	TCP	AGTGGGTCCCGAGAAAACACCTATTTAATT
Ma06_g26850	YABBY	TAATAATAAT
Ma06_g26850	YABBY	TAATAATAAT
Ma06_g26850	YABBY	TAATAATAAT
Ma06_g26850	YABBY	TAATAATAAT

Ma06_g26850	YABBY	TAATAATAAT
Ma06_g26850	YABBY	TAATAATAAT
Ma06_g26850	YABBY	TAATAATAAT
Ma06_g26850	TCP	GTGGGTCCCGA
Ma06_g26850	MIKC_MADS	TATTGTCCTCTTTGGGGTA
Ma06_g26850	MIKC_MADS	TATTGTCCTCTTTGGGGTA
Ma06_g26850	TALE	GGTGACGTCA
Ma06_g26850	GATA	ATTGATCATTATAATAATAAAGACAAT
Ma06_g26850	MIKC_MADS	ATTGTCCTCTTTGGG
Ma06_g26850	bHLH	GGCCATGTGA
Ma06_g26850	NAC	CTTGTTCGACACGCAG
Ma06_g26850	TALE	TCGTTCTCCTCTGCCTCGGC
Ma06_g26850	TCP	GGGACCAT
Ma06_g26850	WOX	TCAATCAA
Ma06_g26850	WOX	TCAATCAA
Ma06_g26850	BBR-BPC	ACTAAAGAGGGAGATGGATAAAGA
Ma06_g26850	TCP	CAGTGGACCCAAC
Ma06_g26850	ERF	ACCACCTCCGCTGCAATCGAT
Ma06_g26850	HD-ZIP	TCATCAATAATAATAATAATA
Ma06_g26850	HD-ZIP	TCATTTAATAT
Ma06_g26850	NAC	TGCTTGTTCGACACG
Ma06_g26850	ERF	TACCACCTCCGCTGCAATCG
Ma06_g26850	TCP	GGGACCAG
Ma06_g26850	TCP	GGGACCAG
Ma06_g26850	G2-like	GAGAAAACA
Ma06_g26850	HD-ZIP	AAATATAATGATAAA
Ma06_g26850	NAC	CTTGTTCGACACGCA
Ma06_g26850	bZIP	ACTGACGTCGT
Ma06_g26850	ARF	ATTTTGATTTCAACGTGAAAT
Ma06_g26850	TCP	ATTGGGACCAGGA
Ma06_g26850	TCP	ATTGGGACCAGGA
Ma06_g26850	NAC	CTTGTTCGACACG
Ma06_g26850	TCP	GAGTGGGTCCCGA
Ma06_g26850	HD-ZIP	ACATCTAATGATTGC
Ma06_g26850	HD-ZIP	TAATAATT
Ma06_g26850	NAC	TAGAGTGTCATCCAAGC
Ma06_g26850	G2-like	TTCAGATTCGATTA
Ma06_g26850	HD-ZIP	AATCAATCAA
Ma06_g26850	MYB_related	GTACTTATCCACATC
Ma06_g26850	YABBY	TTATAATAAT
Ma06_g26850	C2H2	CAATCAAGACAATAA
Ma06_g26850	TCP	GGGACCAG

Ma06_g26850	TCP	GGGACCAG
Ma06_g26850	MYB	GGGACGTTACA
Ma06_g26850	MYB	GGGACGTTACA
Ma06_g26850	SBP	ATCATTGTACCCAATT
Ma06_g26850	SRS	AGCCTAGGGG
Ma06_g26850	HD-ZIP	TCATTTAATAT
Ma06_g26850	MIKC_MADS	ATTCCCAATTGGGAC
Ma06_g26850	ERF	ATACCACCTCCGCTGCAATCG
Ma06_g26850	ERF	ATACCACCTCCGCTGCAATCG
Ma06_g26850	B3	ATATTTATTTTGTGTGGAAAA
Ma06_g26850	NAC	AGAGTGTCATCCAAGCA
Ma06_g26850	MYB_related	GTGTACTTATCCACA
Ma06_g26850	ERF	CCACCTCCGCTGCAATCGATC
Ma06_g26850	ERF	CCACCTCCGCTGCAATCGATC
Ma06_g26850	YABBY	TATGATAA
Ma06_g26850	GATA	GGGGATAATTATTATTATTATTATTAT
Ma06_g26850	bZIP	GACTGACGTCGT
Ma06_g26850	bZIP	GACTGACGTCGT
Ma06_g26850	bZIP	GACTGACGTCGT
Ma06_g26850	B3	ATTTTGATTTCAACGTGAAAT
Ma06_g26850	TALE	CGGTCGTCGTTCTCCTCTGC
Ma06_g26850	TALE	ACTGACGTCG
Ma06_g26850	HD-ZIP	ATAATAATTTG
Ma06_g26850	MIKC_MADS	CTATTCCCAATTGGG
Ma06_g26850	SRS	GCCGTAGGGC
Ma06_g26850	AP2	AATAATAAAGACAATAAAGA
Ma06_g26850	SBP	AGTGTACTTAT
Ma06_g26850	C2H2	CATTTAACACCTTTTTTA
Ma06_g26850	bZIP	ATGACTGACGTCGTG
Ma06_g26850	NAC	GTGCTTGTTCGACACGCA
Ma06_g26850	MYB	TCTACCAACTGAACC
Ma06_g26850	C2H2	CAGATGACACATAAATCA
Ma06_g26850	GATA	TGATCATTATAATAA
Ma06_g26850	bZIP	GCGGGTGACGTCAGT
Ma06_g26850	C2H2	CGTTCTCCTCTGCCTCGGC
Ma06_g26850	bZIP	TCTGACATCGTCATT
Ma06_g26850	Dof	ATAATAATAAAGACAATAAAG
Ma06_g26850	MYB_related	TAATATCT
Ma06_g26850	Dof	TACAGGAAAAAGAGAACCGAA
Ma06_g26850	bZIP	ATGACTGACGTCGTG
Ma06_g26850	GATA	TGTCGTCATTATTACCCTCATCAATAA
Ma06_g26850	C2H2	ATAATAAAGACAATAAA

Ma06_g26850	Dof	TAATGATAAAAAGAACACA
Ma06_g26850	HD-ZIP	ATAATAATTTG
Ma06_g26850	bZIP	ACTGACGTCGTGGCG
Ma06_g26850	AP2	TATGAAAGAGATGAAAAACA
Ma06_g26850	C2H2	AAAAAGAGAACC
Ma06_g26850	Dof	ATTATAATAATAAAGACAATA
Ma06_g26850	HD-ZIP	CAATAATT
Ma06_g26850	TCP	ATGGGACCAT
Ma06_g26850	GATA	CCAATAATTGATCATTATAATAATAAA
Ma06_g26850	MYB_related	TTTGTGTTATCCGCA
Ma06_g26850	YABBY	TAATAATAAA
Ma06_g26850	GATA	TCATTATTACCCTCA
Ma06_g26850	TCP	TATGGGACCATAA
Ma06_g26870	MIKC_MADS	CTTTATTTTTTTCTCTCCTA
Ma06_g26870	Dof	ATAGCTTTATTTTTTTTCTCT
Ma06_g26870	SBP	AATTTTGTACCATTAA
Ma06_g26870	MIKC_MADS	ATAAAAAAAGAAAA
Ma06_g26870	Dof	CAGAAATAAAAAAAGAAAATG
Ma06_g26870	C2H2	TAAATACCACCTCCGCTG
Ma06_g26870	G2-like	AAGAAAACA
Ma06_g26870	Dof	TTTATTTTTTTCTCTCCTAT
Ma06_g26870	C2H2	GTATTAAGACAAAAA
Ma06_g26870	AP2	CAGAAATAAAAAAAGAAAAT
Ma06_g26870	HD-ZIP	CAAATAATTGA
Ma06_g26870	C2H2	AACAAAATAACAAAAGT
Ma06_g26870	C2H2	TGTATTAAGACAAAAAAA
Ma06_g26870	MIKC_MADS	ATAAAAAAAGAAA
Ma06_g26870	HD-ZIP	CCAATAATTCA
Ma06_g26870	Dof	AAAATAACAAAAGTGCATC
Ma06_g26870	Dof	AAATAAAAAAAGAAAATGTTC
Ma06_g26870	HD-ZIP	ACATTAATTAA
Ma06_g26870	GATA	CAGATCTGATCTA
Ma06_g26870	GATA	CAGATCTGATCTA
Ma06_g26870	MIKC_MADS	AAATAAAAAAAGAAA
Ma06_g26870	HD-ZIP	CCAATAATTCA
Ma06_g26870	bHLH	CCCACTTGG
Ma06_g26870	MYB_related	GTTCTTATCCTAATC
Ma06_g26870	Dof	TAAAATAAAAAAGGACCCA
Ma06_g26870	C2H2	TGTATTAAGACAAAAAA
Ma06_g26870	ERF	ACCACCTCCGCTGCAATCGAT
Ma06_g26870	C2H2	CCTCGTTCTTATCCTAATC
Ma06_g26870	ERF	TACCACCTCCGCTGCAATCG

Ma06_g26870	YABBY	TATGATTA
Ma06_g26870	YABBY	TATGATTA
Ma06_g26870	ARF	CCAGACAAAA
Ma06_g26870	Dof	TTTATTTTTTAGGATTTTTTA
Ma06_g26870	SBP	TTTTCTGTACAATTAC
Ma06_g26870	HD-ZIP	AATCCAATAATTCACTTAGTT
Ma06_g26870	ERF	ACGCAGGCGGCTGCT
Ma06_g26870	ERF	ACGCAGGCGGCTGCT
Ma06_g26870	C2H2	GATAAAAAACAAATA
Ma06_g26870	Trihelix	TTTTTTACTGAAAT
Ma06_g26870	ARF	ATGTCGGAAT
Ma06_g26870	ARF	ATGTCGGAAT
Ma06_g26870	BBR-BPC	AACAAGCAGAAATAAAAAAAGAAA
Ma06_g26870	HD-ZIP	CAAATAATTGA
Ma06_g26870	MYB	ATGTTTGGTATGG
Ma06_g26870	C2H2	AACAAATAACAAAAT
Ma06_g26870	C2H2	TGATAAAAAACAAATAA
Ma06_g26870	MYB_related	TCGTTCTTATCCTAA
Ma06_g26870	bHLH	TCACATGG
Ma06_g26870	HD-ZIP	ACATTAATTAA
Ma06_g26870	GATA	CTCAGATCTGA
Ma06_g26870	MYB	TCATTAGATAAGG
Ma06_g26870	ERF	ATACCACCTCCGCTGCAATCG
Ma06_g26870	ERF	ATACCACCTCCGCTGCAATCG
Ma06_g26870	bHLH	TTCCATGTGA
Ma06_g26870	B3	GTTTACATGCATTAC
Ma06_g26870	ERF	CCACCTCCGCTGCAATCGATC
Ma06_g26870	ERF	CCACCTCCGCTGCAATCGATC
Ma06_g26870	YABBY	TATGATAA
Ma06_g26870	C2H2	CAGTCCAGACAAAAA
Ma06_g26870	TCP	AGGACCCAC
Ma06_g26870	MIKC_MADS	TTATTTTTTTTCTCTCCTATG
Ma06_g26870	TCP	AAGGACCCAC
Ma06_g26870	Dof	GAAATAAAAAAAGAAAATG
Ma06_g26870	MYB_related	TATATTATCCAAGTC
Ma06_g26870	C2H2	AACAAAATAACAAAAGTG
Ma06_g26870	bHLH	CATGTGACATGCGCA
Ma06_g26870	HD-ZIP	AAAATAATTTT
Ma06_g26870	BBR-BPC	AGCAGAAATAAAAAAAGAAAATGT
Ma06_g26870	TCP	GGACCCAC
Ma06_g26870	C2H2	ACAAAATAACAAAAG
Ma06_g26870	bHLH	CCACATGG

Ma06_g26870	C2H2	AAAAAGAGAACC
Ma06_g26870	MIKC_MADS	ATAAAAAAGGACC
Ma06_g26870	HD-ZIP	CAATAATT
Ma06_g26870	HD-ZIP	TGCCAAATAATTGATACATAG
Ma06_g26870	HD-ZIP	AGAGATTATGATTAC
Ma06_g26870	NAC	AGTTTACATGCATTACAAG
Ma06_g26890	Dof	AAGAGCAAGAAAAAGGGAAAA
Ma06_g26890	Dof	GAAAAAGGGAAAAA
Ma06_g26890	Dof	AGCAAGAAAAAGGGAAAAAAA
Ma06_g26890	NAC	CTTGTTCGACACGCA
Ma06_g26890	NAC	TGCTTGTTCGACACGCA
Ma06_g26890	HD-ZIP	ACAATAATTGA
Ma06_g26890	ARF	CCCGACAAAA
Ma06_g26890	Dof	GAGCAAGAAAAAGGGAAAA
Ma06_g26890	AP2	AAGAAAAAGGGAAAAAAAGT
Ma06_g26890	AP2	CAAGAAAAAGGGAAAAAAAG
Ma06_g26890	SBP	AATTTTGTACCATTAA
Ma06_g26890	Dof	GAAAAAGGGAA
Ma06_g26890	Dof	AAAAAGGGAAAAAAAGTGGAG
Ma06_g26890	Dof	AAAAAGGGAAAAAAAGTGGAG
Ma06_g26890	HD-ZIP	ACAATAATTGA
Ma06_g26890	Dof	AAAAGGGAAAAAAAGTGGAGT
Ma06_g26890	C2H2	TAAATACCACCTCCGCTG
Ma06_g26890	Dof	GAAAAAGGGAAAAAAAGTGGA
Ma06_g26890	Dof	GAAAAAGGGAAAAAAAGTGGA
Ma06_g26890	HD-ZIP	CCTACAATAATTGAGAACGTA
Ma06_g26890	C2H2	GTATTAAGACAAAAA
Ma06_g26890	C3H	CAAGAAAAAGGGAA
Ma06_g26890	C3H	GAAAAAGGGAA
Ma06_g26890	TCP	AGTGGGTCCCGACAAAACACCTATTTAATT
Ma06_g26890	MIKC_MADS	AGAAAAAGGGAAAA
Ma06_g26890	C2H2	TGTATTAAGACAAAAAAA
Ma06_g26890	AP2	GAGCAAGAAAAAGGGAAAAA
Ma06_g26890	TCP	GTGGGTCCCGA
Ma06_g26890	MIKC_MADS	TTAGTTTAAATGGAAACAA
Ma06_g26890	AP2	GAAAAAGGGAAAAAAAGTGG
Ma06_g26890	CPP	ATAAATTTAAATA
Ma06_g26890	CPP	CATTTGAATG
Ma06_g26890	AP2	GCAAGAAAAAGGGAAAAAAA
Ma06_g26890	G2-like	TTCTGCATATTCTCT
Ma06_g26890	bHLH	GGCCATGTGA
Ma06_g26890	NAC	CTTGTTCGACACGCAG

Ma06_g26890	MYB_related	AACCCTAGCA
Ma06_g26890	BBR-BPC	AAGAGCAAGAAAAAGGGAAAAAAA
Ma06_g26890	Dof	GCAAGAAAAAGGGAAAAAAAG
Ma06_g26890	bZIP	AATAGCTGGCA
Ma06_g26890	bZIP	AATAGCTGGCA
Ma06_g26890	TCP	CAGTGGACCCAAC
Ma06_g26890	WRKY	GAAGGTCAAAGTC
Ma06_g26890	C2H2	TGTATTAAGACAAAAAA
Ma06_g26890	ERF	ACCACCTCCGCTGCAATCGAT
Ma06_g26890	NAC	TGCTTGTTCGACACG
Ma06_g26890	ERF	TACCACCTCCGCTGCAATCG
Ma06_g26890	Trihelix	TCAATCTCTTACCCTAAA
Ma06_g26890	WRKY	AAGGTCAAAG
Ma06_g26890	C3H	AAAAAAGTGGA
Ma06_g26890	MYB	TAATTAGGGTTAGGT
Ma06_g26890	Dof	AAAAAAGTGG
Ma06_g26890	BBR-BPC	GAGCAAGAAAAAGGGAAAAAAAGT
Ma06_g26890	NAC	CTTGTTCGACACGCA
Ma06_g26890	MYB	GTTAGGTTAA
Ma06_g26890	BBR-BPC	AGAAGAGCAAGAAAAAGGGAAAAA
Ma06_g26890	Nin-like	AAATGTCTCTTGATG
Ma06_g26890	NAC	CTTGTTCGACACG
Ma06_g26890	TCP	GAGTGGGTCCCGA
Ma06_g26890	BBR-BPC	TAGTGTCTTTCACTATGTTTC
Ma06_g26890	BBR-BPC	AAGAAAAAGGGAAAAAAAGTGGAG
Ma06_g26890	Dof	AAAAAAGTGGAGTCTATAGAG
Ma06_g26890	Dof	AAAAAAGTGGAGTCTATAGAG
Ma06_g26890	Dof	AAGGGAAAAAAAGTGGAGT
Ma06_g26890	B3	AAAGCTTTTTTGATCAGAAAA
Ma06_g26890	ARF	AAAGCTTTTTTGATCAGAAAA
Ma06_g26890	Dof	AAAAAAGTGGA
Ma06_g26890	MYB_related	TACCCTAAAC
Ma06_g26890	WRKY	GAAGGTCAAAGTC
Ma06_g26890	ERF	ATACCACCTCCGCTGCAATCG
Ma06_g26890	ERF	ATACCACCTCCGCTGCAATCG
Ma06_g26890	B3	ATATTTATTTTGTGTGGAAAA
Ma06_g26890	BBR-BPC	TAAGAAGAGCAAGAAAAAGGGAAA
Ma06_g26890	HD-ZIP	TCATTTAATTC
Ma06_g26890	ERF	CCACCTCCGCTGCAATCGATC
Ma06_g26890	ERF	CCACCTCCGCTGCAATCGATC
Ma06_g26890	Dof	AGGGAAAAAAAGTGGAGTCTA
Ma06_g26890	MYB_related	TTTGCGTTATCCGCA

Ma06_g26890	Dof	AGCAAGAAAAAGGAAAAA
Ma06_g26890	BBR-BPC	GACAGATGATGAGTGGAAGAGGTT
Ma06_g26890	BBR-BPC	CCTAAGAAGAGCAAGAAAAAGGGA
Ma06_g26890	ARF	CCAGACAAAT
Ma06_g26890	BBR-BPC	AAATCTCAATCTCTTACCCTA
Ma06_g26890	HSF	CTAGTAGTTTC
Ma06_g26890	MYB	ATTAGGGTTAGGTTA
Ma06_g26890	MYB	ATTAGGGTTAGGTTA
Ma06_g26890	WRKY	GAAGGTCAAAGTC
Ma06_g26890	C2H2	TGGGTCCCGACAAAACAC
Ma06_g26890	C3H	GGAAAAAAAGTGGA
Ma06_g26890	BBR-BPC	GATATAGAAACCAAGAATGGAAGA
Ma06_g26890	HD-ZIP	TGAAAAATAATTAGATTTATT
Ma06_g26890	BBR-BPC	GCAAGAAAAAGGGAAAAAAAGTGG
Ma06_g26890	NAC	GTGCTTGTTCGACACGCA
Ma06_g26890	Dof	AAAAATTGAAAAGTCTCTA
Ma06_g26890	MYB	GGTTAGGTTA
Ma06_g26890	MYB	AATTAGGGTTAGGTT
Ma06_g26890	C2H2	CAGATGACACATAAATCA
Ma06_g26890	HD-ZIP	CAAATAATTTT
Ma06_g26890	CPP	TATTTGAACA
Ma06_g26890	WRKY	AAGGTCAAAGT
Ma06_g26890	Dof	TACAGGAAAAAGAGAACCGAA
Ma06_g26890	HD-ZIP	AAAATAATTAG
Ma06_g26890	HD-ZIP	GCATTTATGGA
Ma06_g26890	C2H2	GAGAACAAAGGA
Ma06_g26890	MYB	TAATTAGGGTTAGGTTAAGAG
Ma06_g26890	BBR-BPC	GAAAAAGGGAAAAAAAGTGGAGTC
Ma06_g26890	C2H2	AAAAAGAGAACC
Ma06_g26890	HD-ZIP	CAATAATT
Ma06_g26890	WOX	AATCAATTAA
Ma06_g26890	AP2	GAGAACAAAGGAAGGTCAAA
Ma06_g26890	ARF	TCCCGACAAA
Ma06_g30170	GATA	ATGAAGATGATGATG
Ma06_g30170	Dof	AGAAAAATCAAAAAGGAGGAA
Ma06_g30170	bZIP	AAGTGCCACGTGGCA
Ma06_g30170	bZIP	ATAAGTGCCACGTGGCAT
Ma06_g30170	bZIP	AAGTGCCACGTGGCA
Ma06_g30170	Dof	AAAATCAAAAAGGAGGAAGCA
Ma06_g30170	bHLH	GGCACGTGCG
Ma06_g30170	bZIP	TAAGTGCCACGTGGCATA
Ma06_g30170	BBR-BPC	GCGGAACAGAGAGAGAGATGGCGA

Ma06_g30170	bZIP	TGCCACGTGGCATAT
Ma06_g30170	GATA	ATGAAGATGAAGATG
Ma06_g30170	GATA	ATGAAGATGAAGATG
Ma06_g30170	bHLH	AAAAAGGCACGTGCGTAAGAC
Ma06_g30170	bZIP	CCACGTGGCA
Ma06_g30170	Dof	CAAAAAGGAGGAAGCAGAAGA
Ma06_g30170	Dof	CAAAAAGGAGGAAGCAGAAGA
Ma06_g30170	GATA	AAGATGATGATGAGG
Ma06_g30170	BES1	TGCACGTGTAG
Ma06_g30170	bHLH	TGCCACGTGG
Ma06_g30170	GATA	AAGATGAAGATGATG
Ma06_g30170	bHLH	TCCCACGTGA
Ma06_g30170	GATA	GATGAAGATGAAGATGAAGATGATGAT
Ma06_g30170	GATA	AAGACGATGAAGATG
Ma06_g30170	bHLH	GCCACTTGG
Ma06_g30170	bHLH	CACGTGACGTTTCA
Ma06_g30170	bHLH	CACGTGACGTTTCA
Ma06_g30170	bHLH	GCACGTGC
Ma06_g30170	bHLH	GCACGTGC
Ma06_g30170	bHLH	GCACGTGC
Ma06_g30170	bHLH	GCACGTGC
Ma06_g30170	bHLH	GCACGTGC
Ma06_g30170	bHLH	CCCACGTGA
Ma06_g30170	BBR-BPC	GGAACAGAGAGAGAGATGGCGATC
Ma06_g30170	Dof	TAAAAAGGAATAAAGGTAGGA
Ma06_g30170	Dof	TAAAAAGGAATAAAGGTAGGA
Ma06_g30170	GATA	ACGATGAAGATGAAG
Ma06_g30170	ERF	ATGTGTCGGTCGAGT
Ma06_g30170	Dof	TAAAAAGGAATAAA
Ma06_g30170	bHLH	TAAGTGCCACGTGG
Ma06_g30170	C3H	AATCAAAAAGGAGG
Ma06_g30170	HD-ZIP	GAAGATGATGATGAG
Ma06_g30170	bHLH	GCCACGTGG
Ma06_g30170	MIKC_MADS	TTTTTTTCTCATTGATTTTCT
Ma06_g30170	Dof	AAAAATCAAAAAGGAGGAA
Ma06_g30170	Dof	TTTATTTTTTTCTCATTGAT
Ma06_g30170	bHLH	TAAGTGCCACGTGGCATATAT
Ma06_g30170	bHLH	CCACGTGA
Ma06_g30170	bHLH	AAAAAGGCACGTGC
Ma06_g30170	Dof	CATATTAAAAAGGAATAAAGG
Ma06_g30170	GATA	GAGATTGTTCATTGTGGTCATCATCTC
Ma06_g30170	bHLH	CACGTGGCATATATG

Ma06_g30170	HD-ZIP	ATAACAATAATAGGTAGGAAA
Ma06_g30170	BES1	TTGCACGTGTAGACACATCT
Ma06_g30170	HD-ZIP	AAAATAATTAA
Ma06_g30170	HD-ZIP	TCATTAAATAA
Ma06_g30170	MYB	GAAGATGAAGATGATGATGAG
Ma06_g30170	HD-ZIP	TCATTAAATAA
Ma06_g30170	YABBY	TATGATTA
Ma06_g30170	bHLH	CCCACTTGA
Ma06_g30170	bHLH	GCACGTGC
Ma06_g30170	MYB	GAAGATGAAGATGAAGATGAT
Ma06_g30170	BES1	TTGCACGTGTAGACA
Ma06_g30170	Dof	TAAAAAGGAATAAAGGTAGGA
Ma06_g30170	bHLH	GCACGTGT
Ma06_g30170	bHLH	GCACGTGT
Ma06_g30170	bHLH	GCACGTGT
Ma06_g30170	bHLH	CCACGTGG
Ma06_g30170	BES1	TCCCACGTGACGTTT
Ma06_g30170	TCP	TGGACCCAC
Ma06_g30170	B3	TTGCATGCT
Ma06_g30170	GATA	GAAGATGAAGATGAAGATGATGATGAG
Ma06_g30170	ARF	TTATTATTTATAGGTGGAAAA
Ma06_g30170	bHLH	CACGTGCGTAAGACT
Ma06_g30170	bHLH	GCACGTGT
Ma06_g30170	HD-ZIP	ATGTCTAATGATTGC
Ma06_g30170	GATA	AAGATGAAGATGAAG
Ma06_g30170	BES1	GGCACGTGCGT
Ma06_g30170	BES1	AGGCACGTGCGTAAG
Ma06_g30170	ARF	TATATATTTGTAGCAGGACAG
Ma06_g30170	ERF	ACAGCAGCGCCGTTG
Ma06_g30170	bHLH	GGCACGTGC
Ma06_g30170	bHLH	CACGTGCGTAAGAC
Ma06_g30170	bHLH	CACGTGCGTAAGAC
Ma06_g30170	TCP	TGGGACCCGC
Ma06_g30170	MYB	GACGATGAAGATGAAGATGAA
Ma06_g30170	MYB	ATTTTGAGTTAGGCT
Ma06_g30170	HD-ZIP	TAATTAATTAC
Ma06_g30170	ERF	ATGTGTCGGTCGAGT
Ma06_g30170	bHLH	GCACGTGT
Ma06_g30170	TCP	ATGGACCCAC
Ma06_g30170	BES1	AGGCACGTGCGTAAGACTAT
Ma06_g30170	bHLH	TGCACGTGTA
Ma06_g30170	C2H2	GGAAGCAGAAGA

Ma06_g30170	bHLH	GCCACGTGGC
Ma06_g30170	GATA	GACGATGAAGATGAAGATGAAGATGAT
Ma06_g30170	HD-ZIP	AAAATAATTAA
Ma06_g30170	TCP	CCATGGGACCCGC
Ma06_g30170	B3	CAGCATGCC
Ma06_g30170	Dof	CAAAAAGGAGGAAG
Ma06_g30170	bHLH	CACGTGACGTTTCAA
Ma06_g30170	bHLH	GCACGTGC
Ma06_g30170	bHLH	CCACGTGA
Ma06_g30170	bZIP	CCACGTGG
Ma06_g30170	MIKC_MADS	CATCATC
Ma06_g30170	BBR-BPC	AACAGAGAGAGAGATGGCGATCGC
Ma06_g30170	GATA	ACAAGATCTAG
Ma06_g30170	bHLH	CACGTGGCATATAT
Ma06_g30170	bHLH	CACGTGGCATATAT
Ma06_g30170	Dof	AAAAAGGAATAAAGGTAGGAC
Ma06_g30170	Dof	AAAAAGGAATAAAGGTAGGAC
Ma06_g30170	MYB	GATTTTGAGTTAGGC
Ma06_g30170	MYB_related	GAGATACTT
Ma06_g30170	C2H2	AAGTGAAGAAA
Ma06_g30170	ERF	ACAGCAGCGCCGTTG
Ma06_g30170	bHLH	AGGCACGTGC
Ma06_g30170	bHLH	CCACGTGG
Ma06_g30170	GATA	AATGCTGATAATGTTAATAACAGTAAT
Ma06_g30170	TCP	GGACCCAC
Ma06_g30170	ERF	ATGTGTCGGTCGA
Ma06_g30170	ERF	ATGTGTCGGTCGA
Ma06_g30170	MYB	TTTTGAGTTAGGCTA
Ma06_g30170	MYB	TTTTGAGTTAGGCTA
Ma06_g30170	B3	TATTATTTATAGGTGGAAAAA
Ma06_g30170	CPP	ATAATTTTTAAAA
Ma06_g30170	Dof	TGTCATATTAAAAAGGAATAA
Ma06_g30170	Dof	GAAAAATCAAAAAGGAGGAAG
Ma06_g30170	Dof	GAAAAATCAAAAAGGAGGAAG
Ma06_g30170	HD-ZIP	TAAATAATGGA
Ma06_g30170	C2H2	CGGAACAGAGAG
Ma06_g30170	HD-ZIP	TCATTTATTTT
Ma06_g30170	MYB	ATTAGGTGTA
Ma06_g30170	C3H	TAAAAAGGAAT
Ma06_g30170	BBR-BPC	AAGAAAAATCAAAAAGGAGGAAGC
Ma06_g30170	Dof	AAAAAGGAGGAAGCAGAAGAC
Ma06_g30170	Dof	AAAAAGGAGGAAGCAGAAGAC

Ma06_g30170	TCP	ATGGGACCCGC
Ma06_g30170	Dof	TCATATTAAAAAGGAATAA
Ma06_g30170	bZIP	GTGCCACGTGGC
Ma06_g30170	bZIP	GTGCCACGTGGC
Ma06_g30170	bZIP	GTGCCACGTGGC
Ma06_g30170	Dof	TTATTTTTTTCTCATTGATT
Ma06_g30170	Dof	TATTTTTTTCTCATTGATTT
Ma06_g30170	ERF	ATGTGTCGGTCGAGTGA
Ma06_g30170	BES1	TGCCACGTGGCATAT
Ma06_g30170	G2-like	ATACATATATTCTAA
Ma06_g30170	B3	TTATTATTTATAGGTGGAAAA
Ma06_g30170	Dof	AATATTTTATTTTTTTCTCA
Ma06_g30170	HSF	CGCGAAGGTTC
Ma06_g30170	NAC	GATGTGTAGTACAAG
Ma06_g30170	bHLH	CCCACGTGAC
Ma06_g30170	C2H2	AAGAAAAATCAAAAA
Ma06_g30170	CPP	GTTTTGAATT
Ma06_g30170	C3H	GTGGAAAAAGGCAC
Ma06_g30170	bHLH	TTGCACGTGT
Ma07_g09040	BBR-BPC	GAGAGAGAGAGAGAGAGAGAGAGC
Ma07_g09040	BBR-BPC	GAGAGAGAGAGAGAGAGAGCAA
Ma07_g09040	BBR-BPC	ACGAGAGAGAGAGAGAGAGAGAGA
Ma07_g09040	BBR-BPC	GAGAGAGAGAGAGAGAGAGCAAAA
Ma07_g09040	BBR-BPC	GAGAGAGAGAGAGAGAGCAAAAAA
Ma07_g09040	BBR-BPC	AGACGAGAGAGAGAGAGAGAGAGA
Ma07_g09040	BBR-BPC	GATTGGAGACGAGAGAGAGAGAGA
Ma07_g09040	MYB	TTGGAAGAGTTTGGTGAAAGA
Ma07_g09040	AP2	GAGAGAGAGAGAGAGAGAGA
Ma07_g09040	AP2	GAGAGAGAGAGAGAGAGAGA
Ma07_g09040	BBR-BPC	GAGAGAGAGAGAGAGCAAAAAAAG
Ma07_g09040	BBR-BPC	TTGGAGACGAGAGAGAGAGAGAGA
Ma07_g09040	SBP	TATTATGTACCACTTT
Ma07_g09040	BBR-BPC	GGAGACGAGAGAGAGAGAGAGAGA
Ma07_g09040	BBR-BPC	GAGAGAGAGAGCAAAAAAAGATAA
Ma07_g09040	AP2	GGAGACGAGAGAGAGAGAGA
Ma07_g09040	AP2	ACGAGAGAGAGAGAGAGAGA
Ma07_g09040	MYB	TTGGAAGAGTTTGGT
Ma07_g09040	MYB	AGAGTTTGGTGAAAG
Ma07_g09040	Dof	AAAAAAGATAATAGAAGGTAT
Ma07_g09040	Dof	AAAAAAGATAATAGAAGGTAT
Ma07_g09040	Trihelix	TTATTTACCATGTT
Ma07_g09040	AP2	GAGAGAGAGAGAGCAAAAAA

Ma07_g09040	Dof	AGAGAGCAAAAAAAGATAATA
Ma07_g09040	BBR-BPC	GAGAGAGAGAGAGCAAAAAAAGAT
Ma07_g09040	MYB	GAGTTTGGTGAAA
Ma07_g09040	AP2	AGACGAGAGAGAGAGAGAGA
Ma07_g09040	MYB	ATTATATTAGGTAAA
Ma07_g09040	MYB	TAATTGGAAGAGTTTGGTGAA
Ma07_g09040	HD-ZIP	GTAGGCAATGATTAA
Ma07_g09040	WRKY	AGTTGACTATACTTGACTA
Ma07_g09040	E2F/DP	AAGATAATTTTCCCGAAATAA
Ma07_g09040	Dof	GAGCAAAAAAAGATAATAGAA
Ma07_g09040	G2-like	GGAATCTTGAA
Ma07_g09040	MYB	GAAGAGTTTGGTGAA
Ma07_g09040	C2H2	AAGATAGCACCTCCGAGT
Ma07_g09040	AP2	GAGAGAGAGAGAGAGAGAGC
Ma07_g09040	AP2	GAGAGAGAGAGAGAGCAAAA
Ma07_g09040	AP2	GCAAAAAAAGATAATAGAAG
Ma07_g09040	G2-like	GGAATCTT
Ma07_g09040	MIKC_MADS	TGTTTCTTACTTCTTCCTTCC
Ma07_g09040	MIKC_MADS	GCAAAAAAAGATAA
Ma07_g09040	WRKY	GGAGTTGACTATAC
Ma07_g09040	WRKY	GGAGTTGACTAT
Ma07_g09040	BBR-BPC	GAGAGAGAGCAAAAAAAGATAATA
Ma07_g09040	MYB	GAGTTTGGTGA
Ma07_g09040	C3H	TAAGAAAAAGATAA
Ma07_g09040	EIL	ATTAAGATTCATTGA
Ma07_g09040	AP2	AGAGCAAAAAAAGATAATAG
Ma07_g09040	MYB	ATATTAGGTAAAT
Ma07_g09040	WRKY	GGAGTTGACTATA
Ma07_g09040	AP2	GAGAGAGAGAGCAAAAAAAG
Ma07_g09040	BBR-BPC	GAGAGAGCAAAAAAAGATAATAGA
Ma07_g09040	BBR-BPC	TATATGGAGAGAGAGAGTAGCGAG
Ma07_g09040	MYB	TATTAGGTAA
Ma07_g09040	G2-like	AGAGAAATATTCTTC
Ma07_g09040	EIL	GACTTGGTACATAGA
Ma07_g09040	EIL	AGGTAAATTCAATGT
Ma07_g09040	Dof	AGAGCAAAAAAAGATAATA
Ma07_g09040	Dof	TACCATAAGAAAAAGATAATT
Ma07_g09040	WRKY	GGAGTTGACTATAC
Ma07_g09040	MIKC_MADS	GCAAAAAAAGATA
Ma07_g09040	WRKY	AATTGACTTATTA
Ma07_g09040	MYB	GAAGAGTTTGGTGAA
Ma07_g09040	ARF	CTAATGGTTTAGACTCAACAA

Ma07_g09040	MYB	ATTAGGTAAA
Ma07_g09040	MYB	TTTAAGATTATATTAGGTAAA
Ma07_g09040	MYB	GATTATATTAGGTAA
Ma07_g09040	MYB	GATTATATTAGGTAA
Ma07_g09040	WRKY	GGAGTTGACTATA
Ma07_g09040	HSF	AGTATAATCTGGAAC
Ma07_g09040	MIKC_MADS	CATCATC
Ma07_g09040	GATA	TCTTGTTCATCTTCT
Ma07_g09040	MYB	AAGATTATATTAGGT
Ma07_g09040	bZIP	TCTACAGCTGAG
Ma07_g09040	WRKY	AGTTGACTATACT
Ma07_g09040	BBR-BPC	TGTATATGGAGAGAGAGAGTAGCG
Ma07_g09040	WRKY	ATAATTGACTTAT
Ma07_g09040	HD-ZIP	TAGGCAATGATTAACTCTAAT
Ma07_g09040	AP2	AGAGAGCAAAAAAAGATAAT
Ma07_g09040	NAC	TAGAGACGTACATAA
Ma07_g09040	C2H2	CACCTCCACTT
Ma07_g09040	Dof	GAAAAAGATAATTT
Ma07_g09040	BES1	ACACAAGTGTGAATT
Ma07_g09040	YABBY	AATGATTA
Ma07_g09040	MYB	GTTTGGTGAA
Ma07_g09040	MYB	AAAAAAGATAATA
Ma07_g09040	MYB	ATTATATTAGGTAAA
Ma07_g09040	ARF	TGGTCGGGAT
Ma07_g09040	ARF	TGGTCGGGAT
Ma07_g11670	NAC	TGCTTGTTGAAGAAGCA
Ma07_g11670	NAC	GGATGCTTGTTGAAGAAGCAA
Ma07_g11670	NAC	GCTTGTTGAAGAAGCAAC
Ma07_g11670	NAC	GGATGCTTGTTGAAGAAGCAA
Ma07_g11670	NAC	CTTGTTGAAGAAGCAACAT
Ma07_g11670	NAC	ATGCTTGTTGAAGAAGCAA
Ma07_g11670	NAC	TGCTTGTTGAAGAAG
Ma07_g11670	NAC	TGCTTGTTGAAGAAG
Ma07_g11670	MIKC_MADS	CGAAAAGAGGAAAT
Ma07_g11670	ARF	TTTGTCGGC
Ma07_g11670	G2-like	AAAAGAATGTA
Ma07_g11670	NAC	CTTGTTGAAGAAGCAA
Ma07_g11670	NAC	CTTGTTGAAGAAGCAA
Ma07_g11670	NAC	GGATGCTTGTTGAAGAAGCAA
Ma07_g11670	NAC	CTTGTTGAAGAAGCAA
Ma07_g11670	MYB	TTCCAAAACCGAAAC
Ma07_g11670	GATA	TCGATCCGATGAT

Ma07_g11670	GATA	TCGATCCGATGAT
Ma07_g11670	MIKC_MADS	CTCGAAGAGGAAAG
Ma07_g11670	Dof	CGAAAAGAGGAAATGAGAACA
Ma07_g11670	Dof	CGAAAAGAGGAAATGAGAACA
Ma07_g11670	NAC	ATGCTTGTTGAAGAAGCA
Ma07_g11670	GATA	CAATGTCAAGAGAACTAACATCTTGAT
Ma07_g11670	Dof	CAAAAAGAAGCAGGAGAGTCA
Ma07_g11670	Dof	CAAAAAGAAGCAGGAGAGTCA
Ma07_g11670	MYB	CTCACCTACTGCCAC
Ma07_g11670	HD-ZIP	GATCCAATGATTTATCAATTT
Ma07_g11670	bZIP	TAGGGGAGACGTGGATGC
Ma07_g11670	NAC	CTTGTTGAAGAAGCA
Ma07_g11670	NAC	CTTGTTGAAGAAGCA
Ma07_g11670	GATA	TCAAGATCTAA
Ma07_g11670	bZIP	AGGGGAGACGTGGAT
Ma07_g11670	G2-like	GAAAATCTGT
Ma07_g11670	TCP	TGGACCAC
Ma07_g11670	bZIP	GGGAGACGTGGA
Ma07_g11670	bZIP	GGGAGACGTGGA
Ma07_g11670	bZIP	GGGAGACGTGGA
Ma07_g11670	NAC	ATGCTTGTTGAAGAAGCAA
Ma07_g11670	ARR-B	GTAGATTCGG
Ma07_g11670	bHLH	TAGGGGAGACGTGG
Ma07_g11670	ERF	GTTTGTCGGCCGATT
Ma07_g11670	YABBY	AAATCATAAT
Ma07_g11670	TCP	GATGGGTCCAAACACCACAAGCCATTCTGC
Ma07_g11670	Dof	AATTGGATGAAAAAGAAAGCA
Ma07_g11670	NAC	ATGCTTGTTGAAGAAGC
Ma07_g11670	bZIP	AGACGTGG
Ma07_g11670	EIL	ATATAGATCCAATGA
Ma07_g11670	Dof	GAAAAAGAAAGCAGCCAGAAG
Ma07_g11670	Dof	GAAAAAGAAAGCAGCCAGAAG
Ma07_g11670	MIKC_MADS	AACTCGAAGAGGAAA
Ma07_g11670	MYB	ACAAAATTGGATGAAAAAGAA
Ma07_g11670	NAC	CGGATGCTTGTTGAAGAAG
Ma07_g11670	bZIP	AGGGGAGACGTGGAT
Ma07_g11670	MIKC_MADS	CAAAAAGAAGCAGG
Ma07_g11670	MIKC_MADS	ATGCCGAAAAGAGGAAATG
Ma07_g11670	NAC	CCACAAGCCATT
Ma07_g11670	bHLH	CACGTGAAACACAGT
Ma07_g11670	bHLH	CTACCAGCACGTGAAACACAG
Ma07_g11670	G2-like	AGAATCTACAG

Ma07_g11670	CPP	TTAAATTTAAATT
Ma07_g11670	ERF	GTTTGTCGGCCGA
Ma07_g11670	ERF	GTTTGTCGGCCGA
Ma07_g11670	NAC	AGACGTGGATGCACCGGCAT
Ma07_g11670	TCP	CTATGGACCACAT
Ma07_g11670	Dof	AAGAAAGCAGCCAGAAGATAA
Ma07_g11670	Dof	AAGAAAGCAGCCAGAAGATAA
Ma07_g11670	C2H2	GAAAGCAGAGAT
Ma07_g11670	G2-like	AGAATCTACAG
Ma07_g11670	bZIP	AGGGGAGACGTGGAT
Ma07_g11670	HSF	GAACGATCTAGG
Ma07_g11670	WRKY	AACCTTGACTCTGTT
Ma07_g11670	MYB	TCCAAAACCGAAA
Ma07_g11670	G2-like	CCACAAATATTCCCC
Ma07_g11670	CPP	AATTTAAATT
Ma07_g11670	C2H2	AAAAAGAAAGCA
Ma07_g11670	AP2	ACAAAATTGGATGAAAAAGA
Ma07_g11670	ARF	ACCGACCAAA
Ma07_g11670	MIKC_MADS	CTATATATGGAGA
Ma07_g11670	C2H2	CTTTGTTTTGTCTGCTATAG
Ma07_g11670	ARR-B	ATAGATATGC
Ma07_g11670	bHLH	AGCACGTGAA
Ma07_g11670	TCP	AGATGGGTCCAAA
Ma07_g11670	bHLH	GCACGTGA
Ma07_g11670	G2-like	AGAATCTA
Ma07_g11670	G2-like	GATAGATATGCCTA
Ma07_g11670	BES1	CCCCTCGTGTGGAAAACTTC
Ma07_g11670	WRKY	GGGTCAAA
Ma07_g11670	bZIP	ACTGACCTCACCAAA
Ma07_g11670	bZIP	AGACGTGGAT
Ma07_g11670	ERF	GTTTGTCGGCCGATT
Ma07_g11670	TCP	TGGGCAGCAC
Ma07_g11670	bHLH	AGCACGTGA
Ma07_g11670	bHLH	CACGTGAAACACAG
Ma07_g11670	bHLH	CACGTGAAACACAG
Ma07_g11670	HSF	GAACGATCTAGG
Ma07_g11670	HSF	GAACGATCTAGG
Ma07_g11670	bHLH	GCACGTGA
Ma08_g14590	BBR-BPC	GTGAGAGTGCGAGAAATAGAGAGA
Ma08_g14590	MIKC_MADS	GTTTTTTTTTTTGTTTTTTA
Ma08_g14590	ERF	TGTTGTCGGTGGTAGCA
Ma08_g14590	ERF	TGTTGTCGGTGGTAG

Ma08_g14590	BBR-BPC	TAGTGAGAGTGCGAGAAATAGAGA
Ma08_g14590	Dof	TTTTTTTTTTTTGTTTTTTAC
Ma08_g14590	MIKC_MADS	TTTTTTTTTTTGTTTTTTACA
Ma08_g14590	TALE	CCGTTCCCCCTCCCTTCTCC
Ma08_g14590	Dof	GTTTTTTTTTTTTGTTTTTTA
Ma08_g14590	MIKC_MADS	TTTTTTTTTTTTGTTTTTTAC
Ma08_g14590	ERF	GTTGTCGGTGGTAG
Ma08_g14590	ERF	GTTGTCGGTGGTAG
Ma08_g14590	TALE	TCGCCGTTCCCCCTCCCTTC
Ma08_g14590	BBR-BPC	GAGAGTGCGAGAAATAGAGAGATC
Ma08_g14590	bHLH	ATTGTGCCACGTGCCCAAATG
Ma08_g14590	MYB_related	GAGATATTT
Ma08_g14590	C2H2	TTCCCCCTCCCTTCTCCTC
Ma08_g14590	bHLH	CCACGTGC
Ma08_g14590	bHLH	CCACGTGC
Ma08_g14590	bHLH	GCCACGTGC
Ma08_g14590	Dof	TTTTTTTTTTGTTTTTTACAC
Ma08_g14590	ERF	ACTGTTGTCGGTGGT
Ma08_g14590	ERF	TGTTGTCGGTGGT
Ma08_g14590	ERF	TGTTGTCGGTGGT
Ma08_g14590	MYB_related	AAGATATTT
Ma08_g14590	ERF	TGTTGTCGGTGGTAG
Ma08_g14590	Dof	TTTTTTTTTTTGTTTTTTACA
Ma08_g14590	TALE	TTCCCCCTCCCTTCTCCTCG
Ma08_g14590	HD-ZIP	AAAAATAATAATTAA
Ma08_g14590	bHLH	GCCACGTGCC
Ma08_g14590	Dof	CATTTTTTTTTAATTTTTCTCT
Ma08_g14590	HD-ZIP	ATAATAATTAA
Ma08_g14590	TALE	CCCTCCCTTCTCCTCGCTAT
Ma08_g14590	MYB	CGATCAACCACCTTC
Ma08_g14590	MYB	GTCGATCAACCACCTTCTT
Ma08_g14590	HD-ZIP	ATAATAATTAA
Ma08_g14590	C2H2	AAACCAGAGAACATG
Ma08_g14590	bHLH	ATTGTGCCACGTGC
Ma08_g14590	WOX	TCATACAATCA
Ma08_g14590	MYB_related	GAGATATTT
Ma08_g14590	MIKC_MADS	TTTTTTTTTTGTTTTTTACAC
Ma08_g14590	Nin-like	ACATGACTTTTGAAT
Ma08_g14590	BBR-BPC	ATGAGATAAAGATTGGGAGATATT
Ma08_g14590	C2H2	TTATTTTTTGTAGGTGTCTT
Ma08_g14590	bHLH	CCACGTGC
Ma08_g14590	Dof	CAAGTTTTTTTTTTTTGTTTT

Ma08_g14590	MIKC_MADS	CTTCTTCCTTTCTGTCTGCAT
Ma08_g14590	G2-like	GGCATCTT
Ma08_g14590	bHLH	TGCCACGTGC
Ma08_g14590	Dof	AATAAAGAAACCAGGATAGGT
Ma08_g14590	Dof	AATAAAGAAACCAGGATAGGT
Ma08_g14590	HD-ZIP	AAAATAATAATTAACTATTTC
Ma08_g14590	Trihelix	GCACTTACAAACCCTTAA
Ma08_g14590	HD-ZIP	TAATAATT
Ma08_g14590	BES1	TGCCACGTGCCCAAA
Ma08_g14590	Dof	ATTTTTTTTAATTTTTCTCTT
Ma08_g14590	B3	CGGCATGCC
Ma08_g14590	Dof	TTTTTTTTAATTTTTCTCTTG
Ma08_g14590	bHLH	CCACGTGC
Ma08_g14590	C2H2	TTTTTTTTTGTTTTTTACAC
Ma08_g14590	G2-like	GAAAATCTGG
Ma08_g14590	BBR-BPC	TCGACATATAGTGAGAGTGCGAGA
Ma08_g14590	NAC	AAGCTTACTCCATAAGATA
Ma08_g14590	bZIP	GATTGTGCCACGTGCCCA
Ma08_g14590	G2-like	GAAAGAATATATTA
Ma08_g14590	EIL	GCTCACGTTCAGTGA
Ma08_g14590	Dof	CCAAGTTTTTTTTTTTTGTTT
Ma08_g14590	GATA	GCCAGATCTTC
Ma08_g14590	bHLH	CCACGTGC
Ma08_g14590	bHLH	CCACGTGC
Ma08_g14590	NAC	TGAAGCTTACTCCATAAGATA
Ma08_g14590	MYB_related	AAATATCC
Ma08_g14590	NAC	TGAAGCTTACTCCATAAGATA
Ma08_g14590	MIKC_MADS	TGCCAAGTTTTTTTTTTTTGT
Ma08_g14590	EIL	GAATGAATCTT
Ma08_g14590	bHLH	CCACGTGC
Ma08_g14590	C2H2	TTTTTTTTTTTTGTTTTTTA
Ma08_g14590	AP2	TATAGTGAGAGTGCGAGAAA
Ma08_g14590	MYB_related	AAGATATTT
Ma08_g14590	MYB_related	TAGATATTC
Ma08_g14590	G2-like	GAATGTTC
Ma08_g14590	G2-like	GAATGTTC
Ma08_g14590	BBR-BPC	CATATAGTGAGAGTGCGAGAAATA
Ma08_g14590	TALE	CCCCCTCCCTTCTCCTCGCT
Ma08_g14590	HD-ZIP	TAAGCAATAATAGCCAGATTG
Ma08_g14590	C2H2	CCCCTCCCTTCTCCTCGCT
Ma08_g14590	G2-like	GAAAGAATATATTA
Ma08_g14590	bHLH	CACGTGCCCAAATG

Ma08_g14590	bHLH	CACGTGCCCAAATG
Ma08_g14590	MYB	ATTGAAGAATTTGTTCAACAA
Ma08_g14590	bZIP	TAAGTTGACATCATG
Ma08_g14590	NAC	CTTACTCCATAAGATA
Ma08_g14590	bZIP	CCCCACGTCATCTGA
Ma08_g14590	G2-like	CAAAGAATAATCTA
Ma08_g14590	Dof	GATAAAGGAAT
Ma08_g14590	C2H2	CCCTTCCCCACATCG
Ma08_g14590	G2-like	GAAAGAATATA
Ma08_g14590	C2H2	GAGCTACAGCACAAA
Ma08_g14590	ARF	GTTGTCGGT
Ma08_g14590	C2H2	CCGTTCCCCCTCCCTTCTC
Ma08_g14590	Dof	GCATTTTTTTAATTTTTCTC
Ma08_g14590	HSF	GAATAATCTAAA
Ma08_g14590	HSF	GAATAATCTAAA
Ma08_g14590	YABBY	AAATAATAAT
Ma08_g14590	MIKC_MADS	GCTGCTTCTTCCTTTCTGTCT
Ma08_g14590	YABBY	TAATAATAAA
Ma08_g14590	G2-like	ATGAGCATATTTC
Ma08_g14590	G2-like	ATGAGCATATTTCG
Ma08_g23400	BBR-BPC	CTCTCTCTCTCTCTCTCTCTC
Ma08_g23400	BBR-BPC	CTCTCTCTCTCTCTCTCTCTC
Ma08_g23400	BBR-BPC	CTCTCTCTCTCTCTCTCTCTC
Ma08_g23400	BBR-BPC	CTCTCTCTCTCTCTCTCTCTC
Ma08_g23400	BBR-BPC	CTCTCTCTCTCTCTCTCTCTC
Ma08_g23400	BBR-BPC	CTCTCTCTCTCTCTCTCTCTC
Ma08_g23400	BBR-BPC	CTCTCTCTCTCTCTCTCTCTC
Ma08_g23400	BBR-BPC	CTCTCTCTCTCTCTCTCTCTC
Ma08_g23400	BBR-BPC	CTCTCTCTCTCTCTCTCTCTC
Ma08_g23400	BBR-BPC	CTCTCTCTCTCTCTCTCTCTC
Ma08_g23400	BBR-BPC	CTCTCTCTCTCTCTCTCTCTA
Ma08_g23400	BBR-BPC	ATCTCTCTCTCTCTCTCTCTC
Ma08_g23400	BBR-BPC	CTCTCTCTCTCTCTCTCTATA
Ma08_g23400	BBR-BPC	ACATCTCTCTCTCTCTCTCTC
Ma08_g23400	BBR-BPC	CTCTCTCTCTCTCTCTATATA
Ma08_g23400	BBR-BPC	CTCTCTCTCTCTCTATATATA
Ma08_g23400	BBR-BPC	CAACATCTCTCTCTCTCTCTC
Ma08_g23400	MIKC_MADS	TCTCTCTCTCTCTCTCTCTCT
Ma08_g23400	MIKC_MADS	TCTCTCTCTCTCTCTCTCTCT
Ma08_g23400	MIKC_MADS	TCTCTCTCTCTCTCTCTCTCT
Ma08_g23400	MIKC_MADS	TCTCTCTCTCTCTCTCTCTCT
Ma08_g23400	MIKC_MADS	TCTCTCTCTCTCTCTCTCTCT

Ma08_g23400	MIKC_MADS	TCTCTCTCTCTCTCTCTCTCT
Ma08_g23400	MIKC_MADS	TCTCTCTCTCTCTCTCTCTCT
Ma08_g23400	MIKC_MADS	TCTCTCTCTCTCTCTCTCTCT
Ma08_g23400	MIKC_MADS	TCTCTCTCTCTCTCTCTCTCT
Ma08_g23400	MIKC_MADS	TCTCTCTCTCTCTCTCTCTCT
Ma08_g23400	MIKC_MADS	TCTCTCTCTCTCTCTCTCTCT
Ma08_g23400	TALE	CTCTCTCTCTCTCTCTCTCT
Ma08_g23400	TALE	CTCTCTCTCTCTCTCTCTCT
Ma08_g23400	TALE	CTCTCTCTCTCTCTCTCTCT
Ma08_g23400	TALE	CTCTCTCTCTCTCTCTCTCT
Ma08_g23400	TALE	CTCTCTCTCTCTCTCTCTCT
Ma08_g23400	TALE	CTCTCTCTCTCTCTCTCTCT
Ma08_g23400	TALE	CTCTCTCTCTCTCTCTCTCT
Ma08_g23400	TALE	CTCTCTCTCTCTCTCTCTCT
Ma08_g23400	TALE	CTCTCTCTCTCTCTCTCTCT
Ma08_g23400	TALE	CTCTCTCTCTCTCTCTCTCT
Ma08_g23400	TALE	CTCTCTCTCTCTCTCTCTCT
Ma08_g23400	BBR-BPC	CTCTCTCTCTCTATATATATA
Ma08_g23400	WRKY	TTTTGACTTTTTC
Ma08_g23400	MIKC_MADS	TCTCTCTCTCTCTCTCTCTAT
Ma08_g23400	WRKY	CCGGTCAACGC
Ma08_g23400	WRKY	GGTTTTGACTTTTTC
Ma08_g23400	TALE	CTCTCTCTCTCTCTCTCTAT
Ma08_g23400	BBR-BPC	ATCAACATCTCTCTCTCTCTC
Ma08_g23400	MYB	TTACCTACCT
Ma08_g23400	BBR-BPC	CTCTCTCTCTATATATATATA
Ma08_g23400	WRKY	GGTTTTGACTTTT
Ma08_g23400	MIKC_MADS	TCTCTCTCTCTCTCTCTATAT
Ma08_g23400	WRKY	GGTTTTGACTTTTT
Ma08_g23400	C2H2	CACCCCCCTCCCCCCCTTC
Ma08_g23400	G2-like	CGTAAAATATTCTTT
Ma08_g23400	C2H2	CCACACCCCCCTCCCCCCC
Ma08_g23400	WRKY	GGTTTTGACTTTTT
Ma08_g23400	WRKY	CGGTCAAC
Ma08_g23400	HD-ZIP	TTAATAATTTA
Ma08_g23400	TALE	CAGCCCTCCTCTGCTTCTCC
Ma08_g23400	MIKC_MADS	TTTGGTTTTGACTTTTTCTCT
Ma08_g23400	MIKC_MADS	TCTCTCTCTCTCTCTATATAT
Ma08_g23400	TCP	GGGACCAT
Ma08_g23400	GATA	GGGATCCGATCAA
Ma08_g23400	GATA	GGGATCCGATCAA
Ma08_g23400	MIKC_MADS	TTTGCATAAATGGTAGAAA

Ma08_g23400	B3	TATCGCGTTTTGGTGGAAAAG
Ma08_g23400	ERF	CGCAGCGAGGGCATCGGCGGT
Ma08_g23400	BBR-BPC	CTCTCTCTATATATATATATA
Ma08_g23400	C2H2	CCCCCTCCCCCCCTTCTAT
Ma08_g23400	HD-ZIP	TTAATAATTTA
Ma08_g23400	TALE	CCCCCCTCCCCCCCTTCTAT
Ma08_g23400	BBR-BPC	GATGAAGAATGATGGACTAAGAGA
Ma08_g23400	GATA	TCAACTCCATTATCA
Ma08_g23400	Dof	CAAAATCTAAAAGTTTAAA
Ma08_g23400	TCP	GGAGGGACCATTG
Ma08_g23400	HD-ZIP	TAATAATT
Ma08_g23400	B3	TTGCATGCT
Ma08_g23400	bZIP	CGTGATGTCATTGTA
Ma08_g23400	C2H2	ACTCATCCCTCTTCTCCCC
Ma08_g23400	C2H2	AGTGACAGTGG
Ma08_g23400	Dof	TTTATTTTTTGCTTTAATAAT
Ma08_g23400	ARF	ATATCGCGTTTTGGTGGAAAA
Ma08_g23400	bZIP	CGTGATGTCAT
Ma08_g23400	YABBY	CAATCATCAT
Ma08_g23400	TALE	CCTCCCCCCCTTCTATCGTC
Ma08_g23400	ERF	CGGCGCCC
Ma08_g23400	BBR-BPC	ACATCAACATCTCTCTCTCTC
Ma08_g23400	AP2	AGGCAAATGTAAAATAGAAA
Ma08_g23400	WRKY	CCGGTCAACG
Ma08_g23400	MIKC_MADS	TCCTATAAGAAGCAA
Ma08_g23400	MIKC_MADS	CCCCAAAAGGAAGG
Ma08_g23400	WRKY	GGTTTTGACTTTT
Ma08_g23400	YABBY	CAATAATAAG
Ma08_g23400	Dof	CCAAAAGGAAGGATAGAAGAC
Ma08_g23400	Dof	CCAAAAGGAAGGATAGAAGAC
Ma08_g23400	ERF	GAGGGCATCGGCGGT
Ma08_g23400	WOX	ATTCAATCAT
Ma08_g23400	C2H2	CCCAGCCCTCCTCTGCTTC
Ma08_g23400	HD-ZIP	TAATTAATTAT
Ma08_g23400	MIKC_MADS	CCCCAAAAGGAAG
Ma08_g23400	HD-ZIP	ATTCAATCAT
Ma08_g23400	ERF	GTGGCCGGCGCCCT
Ma08_g23400	ERF	GTGGCCGGCGCCCT
Ma08_g23400	bZIP	TGCCGTGATGTCATT
Ma08_g23400	MYB_related	GGGATATTT
Ma08_g23400	C2H2	CCCCCCTCCCCCCCTTCTA
Ma08_g23400	HD-ZIP	TTCAATCATCA

Ma08_g23400	ERF	CGGCGCCCTA
Ma08_g23400	ERF	AGCGAGGGCATCGGCGGTCAT
Ma08_g23400	ARF	TTTGCATAAATGGTAGAAAAT
Ma09_g12090	AP2	GAAAAAAAGAAAAAAAAAAA
Ma09_g12090	Dof	AAAAAAGAAAAAAAAAAAGTC
Ma09_g12090	Dof	AAAAAAGAAAAAAAAAAAGTC
Ma09_g12090	AP2	GGAAAAAAAGAAAAAAAAAA
Ma09_g12090	AP2	AAAAAAGAAAAAAAAAAAGT
Ma09_g12090	AP2	CGGAAAAAAAGAAAAAAAAA
Ma09_g12090	AP2	AAAAAAAGAAAAAAAAAAAG
Ma09_g12090	AP2	AAAAGAAAAAAAAAAAGTCA
Ma09_g12090	Dof	TCGGAAAAAAAGAAAAAAAAA
Ma09_g12090	AP2	AAGAAAAAAAAAAAGTCAGG
Ma09_g12090	AP2	TCGGAAAAAAAGAAAAAAAA
Ma09_g12090	MIKC_MADS	TTTCCTTAAACGGATATAT
Ma09_g12090	bZIP	TGTCGTGCCACGTCTTAT
Ma09_g12090	bZIP	TGCCACGTCTTATCG
Ma09_g12090	Dof	AAGAAAAAAAAAAAGTCAGGT
Ma09_g12090	Dof	AAAAAAAGAAAAAAAAAAAGT
Ma09_g12090	Dof	AAAAAAAGAAAAAAAAAAAGT
Ma09_g12090	GATA	CTAACATCATCATCT
Ma09_g12090	GATA	ACATCATCATCTTAT
Ma09_g12090	ERF	GAGGGCGGTGAAGG
Ma09_g12090	ERF	GAGGGCGGTGAAGG
Ma09_g12090	Dof	GAAAAAAAGAAAAAAAAAAAG
Ma09_g12090	Dof	GAAAAAAAGAAAAAAAAAAAG
Ma09_g12090	bZIP	CTACGTGGCA
Ma09_g12090	MIKC_MADS	GGAAAAAAAGAAAA
Ma09_g12090	BBR-BPC	CGGAAAAAAAGAAAAAAAAAAAGT
Ma09_g12090	BBR-BPC	GAAAAAAAGAAAAAAAAAAAGTCA
Ma09_g12090	Dof	GGAAAAAAAGAAAAAAAAAAA
Ma09_g12090	Dof	GGAAAAAAAGAAAAAAAAAAA
Ma09_g12090	ARF	TTTACTTGTATTATAAAAAAA
Ma09_g12090	bZIP	GCAAATTCCACGTGTAAA
Ma09_g12090	NAC	AGGTTACGTAAAGAT
Ma09_g12090	ARF	ACCGACAC
Ma09_g12090	NAC	TTACTTGTATTATAAAAA
Ma09_g12090	ERF	AAGAGAGGGCGGTGAAGGT
Ma09_g12090	MYB	TTTACGTTACA
Ma09_g12090	MYB	TTTACGTTACA
Ma09_g12090	NAC	TAGCTTAACCCATACGACA
Ma09_g12090	bHLH	CAAATTCCACGTGTAAAAGCC

Ma09_g12090	RAV	GATGTTGC
Ma09_g12090	NAC	TAGCTTAACCCATACGACA
Ma09_g12090	WOX	GATCAATCAT
Ma09_g12090	MYB_related	GAGATATTC
Ma09_g12090	CPP	AAAAATTTTGACA
Ma09_g12090	bHLH	TTCCACGTGT
Ma09_g12090	bZIP	ACAAACTACGTGGCA
Ma09_g12090	bZIP	ACAAACTACGTGGCA
Ma09_g12090	C2H2	AGTAAGAAACAAATA
Ma09_g12090	BES1	TTCCACGTGTAAAAG
Ma09_g12090	HD-ZIP	TCAACAATAATATTATAGTAA
Ma09_g12090	ERF	AGAGAGGGCGGTGAA
Ma09_g12090	bHLH	CATATGTCACGTGTTCTCAAA
Ma09_g12090	ERF	AGAGGGCGGTGAAGGTT
Ma09_g12090	B3	TAACACATGCAGATA
Ma09_g12090	HD-ZIP	ATTTATGATGATTGT
Ma09_g12090	YABBY	CAATCATCAT
Ma09_g12090	Dof	AAAAAAGAAAAAAA
Ma09_g12090	bHLH	CATATGTCACGTGT
Ma09_g12090	bZIP	AAATTCCACGTGTAA
Ma09_g12090	Dof	TTTATTTTTAAAATTATTTAT
Ma09_g12090	bZIP	ACAAACTACGTGGCA
Ma09_g12090	SBP	CCGTACGGTT
Ma09_g12090	MYB_related	TTTATTATCTAAAAT
Ma09_g12090	bHLH	TCCACGTGT
Ma09_g12090	MYB	ATAGTTGGTATGG
Ma09_g12090	BES1	TTCCACGTGTAAAAGCCTTC
Ma09_g12090	BBR-BPC	AAAAAAGAAAAAAAAAAAGTCAGG
Ma09_g12090	NAC	CTTAACCCATACGACATAT
Ma09_g12090	bHLH	CCACGTGT
Ma09_g12090	HD-ZIP	GATCAATCAT
Ma09_g12090	bZIP	AAATTCCACGTGTAA
Ma09_g12090	C3H	AAAAAAAAAGTCAG
Ma09_g12090	HD-ZIP	CCTAATCATG
Ma09_g12090	bZIP	TTCCACGTGTAAAAG
Ma09_g12090	C2H2	TAGTAAGAAACAAATAA
Ma09_g12090	bZIP	CACAAACTACGTGGCATG
Ma09_g12090	MIKC_MADS	TATTTCCTTAAACGG
Ma09_g12090	SBP	CCGTACGGT
Ma09_g12090	bHLH	CCACGTGT
Ma09_g12090	MYB_related	TATTTATTATCTAAA
Ma09_g12090	MIKC_MADS	CATCATC

Ma09_g12090	MIKC_MADS	CATCATC
Ma09_g12090	Trihelix	GTTTGTCCGGTGAGA
Ma09_g12090	MYB	TCTAAAGATAAGA
Ma09_g12090	bZIP	CAAATTCCACGTGTAAAA
Ma09_g12090	BES1	TCCACGTGTAA
Ma09_g12090	HD-ZIP	ATCAATCATCA
Ma09_g12090	C2H2	CAAATAATACTTACATAT
Ma09_g12090	bZIP	CCACGTGTAA
Ma09_g12090	ERF	AGAGAGGGCGGTGAA
Ma09_g12090	bHLH	CCACGTGT
Ma09_g12090	MYB_related	GAGATATTC
Ma09_g12090	C2H2	AATTTAATGACAGAAAA
Ma09_g12090	GATA	ATGATGATTGTGAAC
Ma09_g12090	bHLH	CACGTGTTCTCAAA
Ma09_g12090	bHLH	CACGTGTTCTCAAA
Ma09_g12090	HD-ZIP	AACCAATCAC
Ma09_g12090	HD-ZIP	ATCAATCATC
Ma09_g12090	bZIP	ACAAACTACGTGGCA
Ma09_g12090	NAC	CTTAACCCATACGACA
Ma09_g12090	WRKY	ACGGTTGACAATTT
Ma09_g12090	NAC	CTTAGCTTAACCCATACGACA
Ma09_g12090	MYB	TGGATAGTTGGTATG
Ma09_g12090	SBP	TCCGTACGGTT
Ma09_g12090	WOX	AACCAATCAC
Ma09_g12090	bHLH	CTCCGTACGGTT
Ma09_g12090	Dof	AAAAAAAAAAAGTCAGGTTTG
Ma09_g12090	bZIP	TCACGTGT
Ma09_g12090	Dof	TCATCTTTTTTTTAATACAA
Ma09_g12090	G2-like	CACGAGATATTCTTT
Ma09_g12090	bHLH	TCACGTGT
Ma09_g12090	bHLH	CCACGTGT
Ma09_g15420	ERF	GGAGGCGGAGGAGGAGGAGGA
Ma09_g15420	ERF	GGCGGAGGAGGAGGAGGAGGA
Ma09_g15420	bZIP	TGCTGACGTGGC
Ma09_g15420	bZIP	TGCTGACGTGGC
Ma09_g15420	bZIP	TGCTGACGTGGC
Ma09_g15420	bZIP	CGTGCTGACGTGGCC
Ma09_g15420	bZIP	CGTGCTGACGTGGCC
Ma09_g15420	ERF	AGAGGAGGCGGAGGAGGAGGA
Ma09_g15420	ERF	GAGAGGAGGCGGAGGAGGA
Ma09_g15420	Dof	AAAAAGAGGAAAAAGTATACA
Ma09_g15420	bZIP	CGTGCTGACGTGGCC

Ma09_g15420	Dof	AAAGAGGAAAAAGTATACA
Ma09_g15420	WRKY	AACGTTGACTTTAG
Ma09_g15420	WRKY	AACGTTGACTTT
Ma09_g15420	WRKY	AACGTTGACTTTA
Ma09_g15420	WRKY	AACGTTGACTTTAG
Ma09_g15420	BES1	CCACACGTGTGGTCAAACAC
Ma09_g15420	bZIP	TGACGTGGCC
Ma09_g15420	BES1	CACACGTGTGG
Ma09_g15420	ERF	CGGAGGAGGAGGAGGAGGA
Ma09_g15420	WRKY	AACGTTGACTTTA
Ma09_g15420	BES1	CCACACGTGTGGTCA
Ma09_g15420	ERF	AGAGGAGGCGGAGGA
Ma09_g15420	AP2	AGTACAGAAGAAGAAACAAA
Ma09_g15420	ERF	AGGCGGAGGAGGAGGAGGA
Ma09_g15420	Dof	AAGAGGAAAAAGTATACATAA
Ma09_g15420	bZIP	GGCCACGTCGCCTCT
Ma09_g15420	ERF	AGGAGGCGGAGGAGGAGGA
Ma09_g15420	bZIP	CCGTGCTGACGTGGCCAA
Ma09_g15420	AP2	AAGAAACAAAGTAAGAAAAG
Ma09_g15420	BBR-BPC	AACAAAGTAAGAAAAGAAGGGAGA
Ma09_g15420	bZIP	CGTGCTGACGTGGCC
Ma09_g15420	ERF	GAGGAGGCGGAGGAG
Ma09_g15420	ERF	GAGGAGGCGGAGGAG
Ma09_g15420	ERF	AGGAGGCGGAGGAGG
Ma09_g15420	MIKC_MADS	GAAAAAGAGGAAAA
Ma09_g15420	bZIP	ATGGTGGCCACGTCGCCT
Ma09_g15420	Dof	GAAAAAGAGGAAAAAGTATAC
Ma09_g15420	Dof	GAAAAAGAGGAAAAAGTATAC
Ma09_g15420	C2H2	CAAAACAGAACA
Ma09_g15420	bZIP	TTCCACGTAAGCAGT
Ma09_g15420	HD-ZIP	ATCAATCATT
Ma09_g15420	MIKC_MADS	ACAGAAGAAGAAAC
Ma09_g15420	bHLH	AGCCACGTGT
Ma09_g15420	WRKY	TTGGTCAACGC
Ma09_g15420	ERF	GGCGGAGGAGGAGGA
Ma09_g15420	WRKY	AACGTTGACTTTAGA
Ma09_g15420	bHLH	CCACGTGC
Ma09_g15420	bHLH	CCACGTGC
Ma09_g15420	WRKY	CGTTGACTTTAGA
Ma09_g15420	ERF	AAGAGAGGAGGCGGAGGAGGA
Ma09_g15420	HD-ZIP	ATCAATCATTG
Ma09_g15420	Dof	GCCAAGAAAAAGAGGAAAAAG

Ma09_g15420	bZIP	CGTGCTGACGTGGCC
Ma09_g15420	bHLH	AGGAAGCCACGTGT
Ma09_g15420	YABBY	TAATAATAAT
Ma09_g15420	YABBY	TAATAATAAT
Ma09_g15420	ERF	GGAGGAGGAGGAGGA
Ma09_g15420	ERF	GGAGGAGGAGGAGGA
Ma09_g15420	GATA	TAGATCGGATTTC
Ma09_g15420	GATA	TAGATCGGATTTC
Ma09_g15420	BES1	CTCCACGTGCGAATGGTTTT
Ma09_g15420	bHLH	TCCACGTGCG
Ma09_g15420	BBR-BPC	CAGAAGAAGAAACAAAGTAAGAAA
Ma09_g15420	WOX	AATCAATCAT
Ma09_g15420	WRKY	CGTTGACTTTAGAACTTGA
Ma09_g15420	C2H2	AGAAACAAAGTA
Ma09_g15420	Dof	AACAAAGTAAGAAAAGAAGGG
Ma09_g15420	Dof	AACAAAGTAAGAAAAGAAGGG
Ma09_g15420	bZIP	GAATATTCCACGTAAGCA
Ma09_g15420	ERF	CGCCACACCGTCCTCCGACG
Ma09_g15420	BBR-BPC	AAGAAACAAAGTAAGAAAAGAAGG
Ma09_g15420	bZIP	CGTGCTGACGTGGCC
Ma09_g15420	HD-ZIP	ATAATAATTAA
Ma09_g15420	ERF	GAAGAGAGGAGGCGGAGGA
Ma09_g15420	HD-ZIP	ATAATAATTAA
Ma09_g15420	ERF	GGAGGCGGAGGAGGA
Ma09_g15420	HD-ZIP	AATAATAATAATTAA
Ma09_g15420	C2H2	AAAATAAGACAACGA
Ma09_g15420	bHLH	GCCACGTGT
Ma09_g15420	G2-like	GAATATTC
Ma09_g15420	G2-like	GAATATTC
Ma09_g15420	bZIP	TGACGTGG
Ma09_g15420	AP2	CAAGAAAAAGAGGAAAAAGT
Ma09_g15420	ERF	AGAGGAGGCGGAGGA
Ma09_g15420	bHLH	TCCACGTGC
Ma09_g15420	BES1	CTCCACGTGCGAATG
Ma09_g15420	ERF	GAAGAGAGGAGGCGGAGGAGG
Ma09_g15420	BBR-BPC	AAGAGAGGAGGCGGAGGAGGAGGA
Ma09_g15420	AP2	AGGGCAAAAGAAGCTAAAAA
Ma09_g15420	G2-like	TGAATATTCCA
Ma09_g15420	CPP	AGTTTGAATT
Ma09_g15420	bHLH	CCACGTGC
Ma09_g15420	ERF	ACCAATGCAGCAGCACCGCC
Ma09_g15420	CPP	AGAAATTTAAACT

Ma09_g15420	RAV	GATGTTGC
Ma09_g15420	GATA	TCGATCTGATGCC
Ma09_g15420	GATA	TCGATCTGATGCC
Ma09_g15420	WRKY	ATTGGTCAACGCA
Ma09_g15420	AP2	GAAGAAGAAACAAAGTAAGA
Ma09_g15420	HD-ZIP	TAATAATT
Ma09_g15420	WRKY	TTGGTCAACG
Ma09_g15420	TCP	GTGGGGTCCGA
Ma09_g15420	WRKY	TGGTCAAC
Ma09_g15420	bZIP	GATTCCGACGTCATA
Ma09_g15420	ERF	GGAGGCGGAGGAGGA
Ma09_g15420	MYB	AACCCAAACGGATTC
Ma09_g15420	bHLH	CCACGTGC
Ma09_g15420	bZIP	GCTGACGTGGC
Ma09_g15420	EIL	CTATGTATCTA
Ma09_g15420	NAC	CCGTAAGCATGACGGAAC
Ma09_g15420	WRKY	ACGTTGACTT
Ma09_g15420	MYB_related	CAGATATTC
Ma09_g15420	WRKY	ATTGGTCAACGCA
Ma09_g15420	ERF	GCGTGTGATGGTGGC
Ma09_g15420	CPP	AATTTGAACG
Ma09_g15420	WRKY	GTGGTCAAACA
Ma09_g15420	GATA	GTGATGGTGGCCACG
Ma09_g15420	bHLH	CCACGTGC
Ma09_g15420	bHLH	CCACGTGC
Ma09_g15420	Dof	GAAAAAGTAT
Ma09_g15420	C2H2	AAGAAGAGAGGA
Ma09_g15420	bZIP	TAGGAAGCCACGTGTATC
Ma09_g15420	ERF	GACCCACATCCGCCACACGTGTGGTCAA
Ma09_g15420	ERF	AGGAGGAGGAGGAGGAAGG
Ma09_g15420	bHLH	CCACGTGT
Ma09_g15420	bHLH	ACACGTGT
Ma09_g15420	bHLH	CCACGTGC
Ma09_g15420	Dof	CAAGGGCAAAAGAAGCTAAAA
Ma09_g15420	BES1	TCCACGTGCGA
Ma09_g15420	Dof	GTGGCCAAGAAAAAGAGGAAA
Ma09_g15420	C3H	GAGGAAAAAGTATA
Ma09_g15420	C2H2	CTTTCGTCCCTTACTG
Ma09_g15420	NAC	CGTAAGCATGACGGAACAC
Ma09_g15420	ERF	TCGCCACACCGTCCTCCGAC
Ma09_g15420	bHLH	CCACGTGT
Ma09_g15420	bHLH	ACACGTGT

Ma09_g15420	bHLH	AGGAAGCCACGTGTATCGAAC
Ma09_g15420	BBR-BPC	GAGAGGAGGCGGAGGAGGAGGAGG
Ma09_g15420	ERF	CACCGCCCTC
Ma09_g15420	C2H2	AAGAAGAAACAAAGT
Ma09_g15420	HD-ZIP	TAATCAATCATTGCTGGTGTT
Ma09_g15420	bHLH	ACACGTGT
Ma09_g15420	Dof	GAAAAAGTATACAT
Ma09_g15420	C2H2	TGGATCAAACAAAAT
Ma09_g15420	HD-ZIP	AATCAATCAT
Ma09_g15420	bZIP	GGAAGCCACGTGTAT
Ma09_g15420	bHLH	CCACGTGT
Ma09_g15420	BBR-BPC	GAAACAAAGTAAGAAAAGAAGGGA
Ma09_g15420	bHLH	ACTGCTCCACGTGCGAATGGT
Ma09_g15420	MYB_related	CAGATATTC
Ma09_g15420	C2H2	GATCCTTTCGTCCCTTACTG
Ma09_g15420	WRKY	ATTGGTCAACGCA
Ma09_g15420	ERF	GGCGGAGGAGGAGGA
Ma09_g15420	bHLH	CACGTGCGAATGGT
Ma09_g15420	bHLH	CACGTGCGAATGGT
Ma09_g15420	bZIP	AGGAAGCCACGTGTATCG
Ma09_g15420	NAC	CGTAAGCATGACGGAACAC
Ma09_g15420	G2-like	TCCTGAATATTCCAC
Ma09_g15420	Dof	GAAAAAGAGGAAAA
Ma09_g15420	ERF	GAGGCGGAGGAGGAG
Ma09_g15420	ERF	GAGGCGGAGGAGGAG
Ma09_g15420	HD-ZIP	TCAATCATTGC
Ma09_g15420	bHLH	ACACGTGT
Ma09_g15420	ERF	CCACATCCGCCACAC
Ma09_g15420	NAC	CGCGTGTTCTACCTGTCTTCTA
Ma09_g15420	ERF	CCACATCCGCCACAC
Ma09_g15420	bHLH	CACGTGTATCGAACT
Ma09_g15420	bHLH	CACACGTGTG
Ma09_g15420	C3H	GAAAAAGTATA
Ma09_g15420	ERF	TTTCATCCGCCATGGACGACC
Ma09_g15420	ERF	TTTCATCCGCCATGGACGACC
Ma09_g15420	BBR-BPC	AGGAGGCGGAGGAGGAGGAGGAGG
Ma09_g15420	MIKC_MADS	GCAAAAGAAGCTAA
Ma09_g15420	bHLH	CCACGTGT
Ma09_g15420	bHLH	GCCACGTGTA
Ma10_g17560	HD-ZIP	TAAAATAATGATGAA
Ma10_g17560	C2H2	TTTCTTCTTCTTCCTGCTC
Ma10_g17560	WOX	TCAATCATTTA

Ma10_g17560	WRKY	CAAAGTCAACCGT
Ma10_g17560	WRKY	CAAAGTCAACCGT
Ma10_g17560	MIKC_MADS	TTTTTCTTATTCTGTTCTTCT
Ma10_g17560	NAC	CGTGACGTTCAAGTAAAAA
Ma10_g17560	HD-ZIP	ATAATAATTGA
Ma10_g17560	MIKC_MADS	TCCCTCCCTTCTTTTCTTCCT
Ma10_g17560	WRKY	CAAAGTCAACCGT
Ma10_g17560	WRKY	AAAGTCAACC
Ma10_g17560	C2H2	AAAAACAGAAAA
Ma10_g17560	bZIP	ATGACTGACGTGTAA
Ma10_g17560	MYB	ATCTCAACCACAAAC
Ma10_g17560	MYB_related	TTTTTTTTATCCAAT
Ma10_g17560	NAC	CGTGACGTTCAAGTAA
Ma10_g17560	NAC	CGTGACGTTCAAGTAA
Ma10_g17560	NAC	CGTGACGTTCAAGTAA
Ma10_g17560	NAC	ACATTTCTTGTATGACAAT
Ma10_g17560	C2H2	CGATTCAGACAAAAT
Ma10_g17560	HD-ZIP	TTCAATCATTT
Ma10_g17560	C2H2	CTCCCTTCTTTTCTTCCTA
Ma10_g17560	GATA	ACTAGATCTAT
Ma10_g17560	NAC	TCGTGACGTTCAAGTAAA
Ma10_g17560	MIKC_MADS	TCTTATTCTGTTCTTCTCTGG
Ma10_g17560	bZIP	ATGACTGACGTGTAA
Ma10_g17560	GATA	ATGATGGGGATGATT
Ma10_g17560	HD-ZIP	ATAATAATTGA
Ma10_g17560	B3	TTACACATGCAATCT
Ma10_g17560	HD-ZIP	ACAACAATTATTGCGGTGTGA
Ma10_g17560	NAC	CGTGACGTTCAAGTA
Ma10_g17560	C2H2	CTTCGTCTTCGTCTTCGTC
Ma10_g17560	C2H2	CTTCGTCTTCGTCTTCGTC
Ma10_g17560	HD-ZIP	AAAATAATTGT
Ma10_g17560	NAC	GATCTCGTGACGTTCAAGTAA
Ma10_g17560	NAC	TCTCGTGACGTTCAAGTAA
Ma10_g17560	MYB	TTATCTCAACCACAAACTA
Ma10_g17560	YABBY	CTATAATAAT
Ma10_g17560	MIKC_MADS	GCTCTCTTTGTTTTTTTGTAA
Ma10_g17560	HD-ZIP	TCATTTATTAT
Ma10_g17560	Dof	ATAAAAGGTGAATGCAAATAT
Ma10_g17560	Dof	ATAAAAGGTGAATGCAAATAT
Ma10_g17560	YABBY	TATGATTA
Ma10_g17560	Dof	ATTATTTTTTCTTATTCTGTT
Ma10_g17560	MIKC_MADS	CGTCTTCGTCTTCGTCTTCGT

Ma10_g17560	MIKC_MADS	CGTCTTCGTCTTCGTCTTCGT
Ma10_g17560	bZIP	GACTGACGTGTA
Ma10_g17560	bZIP	GACTGACGTGTA
Ma10_g17560	bZIP	GACTGACGTGTA
Ma10_g17560	HD-ZIP	TCATTTATTAT
Ma10_g17560	C2H2	AATTATTTTTCTTATTCTG
Ma10_g17560	Dof	TTTATTTTATAATTTATATT
Ma10_g17560	HD-ZIP	TAATAATT
Ma10_g17560	HD-ZIP	TCTATAATAATTGAAGTCATC
Ma10_g17560	HD-ZIP	TTCAATCATT
Ma10_g17560	ERF	CTTCGTCTTCGTCTTCGTCG
Ma10_g17560	HD-ZIP	TCAATCATTTA
Ma10_g17560	BBR-BPC	AGGAGAAAGAAGGGGGGGGGGGG
Ma10_g17560	B3	CGGCATGCC
Ma10_g17560	C2H2	GCTCTCTTTGTTTTTTTGTA
Ma10_g17560	NAC	CGTGACGTTCAAG
Ma10_g17560	MYB_related	AATCTCTTATCCTTT
Ma10_g17560	HD-ZIP	ATTTCTAATGATTGA
Ma10_g17560	WRKY	AGTGTTGACTAAA
Ma10_g17560	bHLH	ACCACTTGT
Ma10_g17560	NAC	GATCTCGTGACGTTCAAGTAA
Ma10_g17560	MYB_related	TCTCTTATCCTTTAA
Ma10_g17560	BBR-BPC	GAGAAAGAAGGGGGGGGGGGGAA
Ma10_g17560	NAC	CGTGACGTTCAAGTA
Ma10_g17560	NAC	TTTCTTGTATGACAATGA
Ma10_g17560	ARF	TCAGACAAAA
Ma10_g17560	HSF	TAGGAACCCTTCTAGATGT
Ma10_g17560	bZIP	TATGACTGACGTGTAATT
Ma10_g17560	NAC	TCTCGTGACGTTCAAGTAA
Ma10_g17560	MIKC_MADS	CTTCTTCCTGCTCTGTTTTGT
Ma10_g17560	bZIP	ATGACTGACGTGTAA
Ma10_g17560	HD-ZIP	TTTTATAATCATGAG
Ma10_g17560	HD-ZIP	GCACAAATAATAAAAGCTTAA
Ma10_g17560	WRKY	TGTTGACTAAAATAATTGT
Ma10_g17560	C2H2	TAAAAAAAAACAGAAAA
Ma10_g17560	MYB_related	TTTTTTATCCAATCT
Ma10_g17560	bZIP	ATGACTGACGTGTAA
Ma10_g17560	MIKC_MADS	CTTCTTCTTCCTGCTCTGTTT
Ma10_g17560	C2H2	TCCCTCCCTTCTTTTCTTC
Ma10_g17560	WOX	TTTCAATCAT
Ma10_g17560	bZIP	TGACGTGTAA
Ma10_g17560	NAC	GACTGACGTGTAATT

Ma10_g17560	bZIP	ATGACTGACGTGTAA
Ma10_g17560	GATA	TCGTCTTCGTCTTCG
Ma10_g17560	GATA	TCGTCTTCGTCTTCG
Ma10_g17560	GATA	TCGTCTTCGTCTTCG
Ma10_g17560	MIKC_MADS	AAAAAAACAGAAAA
Ma10_g17560	Dof	TTATTTTTCTTATTCTGTTC
Ma10_g17560	G2-like	CAAAAAATATTCCAT
Ma10_g17560	WRKY	AGTGTTGACTAA
Ma10_g17560	NAC	CGATCTCGTGACGTTCAAG
Ma10_g17560	GATA	ATTCGTCTTCGTCTTCGTCTTCGTCTT
Ma10_g17560	C2H2	ATTTTTTCTTATTCTG
Ma10_g17560	Dof	TCTACTTTTCTACCTCGTGCA
Ma10_g17560	C2H2	GGAAACAAAGCT
Ma10_g17560	AP2	TCAACCGTGAGGAAAAGAAG
Ma10_g17560	C2H2	TGATTTCTTGGCGTTTATAG
Ma10_g17560	Dof	CTTACTTTTCATATAATCCTA
Ma10_g17560	C2H2	CGTCTTCGTCTTCGTCTTC
Ma10_g17560	C2H2	CGTCTTCGTCTTCGTCTTC
Ma10_g17560	ERF	CTTCGTCTTCGTCTTCGTCT
Ma10_g17560	HD-ZIP	AAAATAATTGT
Ma10_g17560	Dof	TCTTCTTTTCAATCATTTAAA
Ma10_g17560	MIKC_MADS	CCAATAAAGGGAC
Ma10_g17560	HD-ZIP	ACTAAAATAATTGTTCATAGA
Ma10_g17560	MIKC_MADS	TCTTCGTCTTCGTCTTCGTCT
Ma10_g17560	C2H2	TATTCGTCTTCGTCTT
Ma10_g17560	Dof	AATAAAGGGAC
Ma10_g17560	ARF	TTCAGACAAA
Ma10_g17560	AP2	TAAAAAAAAACAGAAAATAT
Ma10_g17560	bZIP	TGACGTGT
Ma10_g17560	C2H2	CTTCTTCTTCCTGCTCTGT
Ma10_g17560	TCP	GTGGGGTTCATTT
Ma10_g17560	MIKC_MADS	TTTTTTCTTATTCTGTTCT
Ma10_g17560	MIKC_MADS	TTTTTTCTTATTCTGTTCT
Ma10_g17560	MYB	AAAAAAAACAGAAAA
Ma10_g17560	Dof	GATGAACTATAAAAGGTGAAT
Ma10_g25490	BBR-BPC	CTCTCTCTCTCTCTCTCTCTC
Ma10_g25490	BBR-BPC	ATCTCTCTCTCTCTCTCTCTC
Ma10_g25490	BBR-BPC	CTCTCTCTCTCTCTCTCTCAT
Ma10_g25490	BBR-BPC	CTCTCTCTCTCTCTCTCATGC
Ma10_g25490	BBR-BPC	CAAGATCTCTCTCTCTCTCTC
Ma10_g25490	C2H2	TATTGTTTTGTCGTTTTCCT
Ma10_g25490	AP2	CAAAAAATAAAAAAGAGAAA

Ma10_g25490	BBR-BPC	AGATCTCTCTCTCTCTCTCTC
Ma10_g25490	AP2	AAAAATAAAAAAGAGAAAAA
Ma10_g25490	MIKC_MADS	TCTCTCTCTCTCTCTCTCTCT
Ma10_g25490	C2H2	GTTTTGTCGTTTTCCT
Ma10_g25490	TALE	CTCTCTCTCTCTCTCTCTCT
Ma10_g25490	Dof	AAAAATAAAAAAGAGAAAA
Ma10_g25490	Dof	CAAAAAATAAAAAAGAGAAAA
Ma10_g25490	HD-ZIP	ATACCAATAATTAAACTCAAT
Ma10_g25490	MIKC_MADS	TCTCTCTCTCTCTCTCTCTCA
Ma10_g25490	Dof	AAAATAAAAAAGAGAAAAAAC
Ma10_g25490	BBR-BPC	CTCTCTCTCTCTCTCATGCTT
Ma10_g25490	ERF	TGGGGAGGAAACGGAGGTGGA
Ma10_g25490	HD-ZIP	CCAATAATTAA
Ma10_g25490	MIKC_MADS	GTTTTCCTTCTTCCGTTTTTG
Ma10_g25490	ERF	GGGAGGAAACGGAGGTGGA
Ma10_g25490	BBR-BPC	GTCAAGATCTCTCTCTCTCTC
Ma10_g25490	Dof	AAAAAATAAAAAAGAGAAAAA
Ma10_g25490	Dof	AAAAAATAAAAAAGAGAAAAA
Ma10_g25490	AP2	GGGGAAAAAAAAGGAACAAA
Ma10_g25490	Dof	AAAAAAGGAACAAA
Ma10_g25490	MIKC_MADS	TTTTGTCGTTTTCCTTCTTCC
Ma10_g25490	AP2	AATAAAAAAGAGAAAAAACT
Ma10_g25490	AP2	TGAGCAAAAAATAAAAAAGA
Ma10_g25490	ERF	AACGGAGGTGGAAGAGGAGGA
Ma10_g25490	HD-ZIP	CCAATAATTAA
Ma10_g25490	ERF	ATTCCACCGCCGTTG
Ma10_g25490	HD-ZIP	AACCAATCAA
Ma10_g25490	ERF	TCCACCGCCGTTGATTTCCTA
Ma10_g25490	MIKC_MADS	TCTCCTCCTTGCTTTCTTTTA
Ma10_g25490	ERF	CTTTCTTTTATTCCACCGCC
Ma10_g25490	Trihelix	CGATTTCCGGCGATC
Ma10_g25490	BBR-BPC	CTCTCTCTCTCTCATGCTTTC
Ma10_g25490	Dof	AAAAAAGAGAAAAAACTGGAT
Ma10_g25490	Dof	AAAAAAGAGAAAAAACTGGAT
Ma10_g25490	ERF	ATTCCACCGCCGTTG
Ma10_g25490	AP2	TAAAAGAAGAACGAAAGACA
Ma10_g25490	Dof	GGGAAAAAAAAGGAACAAATT
Ma10_g25490	bHLH	ACCACTTGC
Ma10_g25490	ARF	ACCGACAT
Ma10_g25490	ARF	ACCGACAT
Ma10_g25490	MIKC_MADS	TCTCTCTCTCTCTCTCTCATG
Ma10_g25490	Dof	CAAAAAATAAAAAAGAGAAAA

Ma10_g25490	Dof	CAAAAAATAAAAAAGAGAAAA
Ma10_g25490	ERF	GGTGGAAGAGGAGGA
Ma10_g25490	Dof	GTAAAAGAAGAACGAAAGACA
Ma10_g25490	Dof	GTAAAAGAAGAACGAAAGACA
Ma10_g25490	Dof	TGGGGGAAAAAAAAGGAACAA
Ma10_g25490	ERF	GCTTTCTTGATTCCACCGCC
Ma10_g25490	Dof	GGGGAAAAAAAAGGAACAA
Ma10_g25490	BBR-BPC	GCGAAGTAGAGCGAAGGAGACATT
Ma10_g25490	C2H2	GGAAACAGAGAT
Ma10_g25490	C2H2	TAAAAAAGAGAAAAA
Ma10_g25490	ERF	GGAAACGGAGGTGGA
Ma10_g25490	ERF	GGAGGAAACGGAGGT
Ma10_g25490	bHLH	CACGTGATGCATAA
Ma10_g25490	bHLH	CACGTGATGCATAA
Ma10_g25490	Trihelix	TATCAAACAAGCCCTAAC
Ma10_g25490	MIKC_MADS	AAAAAAAAGGAACA
Ma10_g25490	C3H	GAAAAAAAAGGAAC
Ma10_g25490	Trihelix	CCCTAACGGCGAAG
Ma10_g25490	bHLH	ACCACTTGG
Ma10_g25490	ARF	AACCGACATG
Ma10_g25490	ERF	AGGAAACGGAGGTGGAAGA
Ma10_g25490	MYB	GATTCGGTGGCAGTAGGTGAA
Ma10_g25490	ERF	ATTCCACCGCCGTTGATTTCC
Ma10_g25490	ERF	ATTCCACCGCCGTTGATTTCC
Ma10_g25490	WOX	CCAATCAA
Ma10_g25490	WOX	CCAATCAA
Ma10_g25490	ERF	TTGATTCCACCGCCGTTGATT
Ma10_g25490	MIKC_MADS	TTTGTCGTTTTCCTTCTTCCG
Ma10_g25490	C2H2	CAGATAAGACAATGA
Ma10_g25490	BBR-BPC	ATGTCAAGATCTCTCTCTCTC
Ma10_g25490	B3	ATATTCATGCAGTCA
Ma10_g25490	C2H2	CAGATCGGACAAAAT
Ma10_g25490	ERF	ATTCCACCGCCGTTGATTTCC
Ma10_g25490	MIKC_MADS	TCCTTGCTTTCTTTTATTCCA
Ma10_g25490	WOX	AACCAATCAA
Ma10_g25490	AP2	AAGAATTTGGGGGAAAAAAA
Ma10_g25490	MYB_related	AAATATCC
Ma10_g25490	ERF	TAACGGCGAAGTAGA
Ma10_g25490	MYB	GGCAGTAGGTGAATG
Ma10_g25490	C2H2	ACGATCAAGACAAGAAAT
Ma10_g25490	Dof	AAAAAAGAGAAAAA
Ma10_g25490	ERF	AAGTTGATTCGGTGGCAGT

Ma10_g25490	AP2	CGTAAAAGAAGAACGAAAGA
Ma10_g25490	ERF	GGAAACGGAGGTGGA
Ma10_g25490	WOX	TCAATCAG
Ma10_g25490	WOX	TCAATCAG
Ma10_g25490	MIKC_MADS	ATCTATCTTCTTTGAGACA
Ma10_g25490	MIKC_MADS	ATCTATCTTCTTTGAGACA
Ma10_g25490	MYB	GGGTAGGTTAT
Ma10_g25490	C2H2	CCTCATCTCTTGCCTTCTC
Ma10_g25490	NAC	GTTACGCAAA
Ma10_g25490	AP2	TAAAAAAGAGAAAAAACTGG
Ma10_g25490	AP2	GGGAAAAAAAAGGAACAAAT
Ma10_g25490	ERF	CGGCGCCT
Ma10_g25490	Trihelix	CTGTTTCCGTCGAAG
Ma10_g25490	C3H	AAAAAAGGAAC
Ma10_g25490	Nin-like	ACATGTCCCTTCATT
Ma10_g25490	bHLH	CACGTGATGCATAAA
Ma10_g25490	ERF	ATTCCACCGCCGTTG
Ma10_g25490	MYB	AGAACAGATAAGA
Ma10_g25490	MYB_related	AGCCCTAACG
Ma10_g25490	MIKC_MADS	CCTTGCTTTCTTTTATTCCAC
Ma10_g25490	MYB	ATTTTTGGTGG
Ma10_g25490	C2H2	CTTTCTTTTATTCCACCGC
Ma10_g25490	MYB	GTGGCAGTAGGTGAA
Ma10_g25490	C2H2	GGCTAAAGGCCAAGAAT
Ma10_g25490	C2H2	CGATCAAGACAAGAA
Ma10_g25490	EIL	CTCCAAGTTCAATCA
Ma10_g25490	NAC	CTTGGCTATCACG
Ma10_g25490	ARF	ACCGACATGT
Ma10_g25490	MYB	AGTAGGTGAA
Ma10_g25490	ERF	CCCTCCCATCCGCCGGACTCCTCATCTC
Ma10_g25490	MIKC_MADS	TCTCTCTCTCATGCTTTCTTG
Ma10_g25490	BBR-BPC	CTCTCTCTCTCATGCTTTCTT
Ma10_g25490	EIL	GGCAAAATACAAAGC
Ma10_g25490	BBR-BPC	AGCAAAAAATAAAAAAGAGAAAAA
Ma10_g25490	bHLH	TCACGTGA
Ma10_g25490	HD-ZIP	CAATAATT
Ma10_g25490	Dof	CCTACTTTTTGTTCGCTGTTT
Ma10_g25490	C3H	AATAAAAAAGAGAA
Ma10_g25490	C2H2	AACGAAAGACAAGAG
Ma10_g25490	Dof	AAAAAAGGAAC
Ma10_g25490	GATA	TTGATCTGCTCAT
Ma10_g25490	GATA	TTGATCTGCTCAT

Ma10_g25490	C2H2	AAAAAGAGAAAA
Ma10_g25490	C2H2	AAAAAGGAACAAATT
Ma10_g25490	MIKC_MADS	TAAAAAAGAGAAAA
Ma10_g25490	MIKC_MADS	CTTCATTTTTGGTGGTCCTTT
Ma10_g25490	ERF	AGGTGGAAGAGGAGGAAGA
Ma10_g25490	WRKY	GCAGTCAAAGT
